# Stabilization of p21 by mTORC1/4E-BP1 predicts clinical outcome of head and neck cancers

**DOI:** 10.1038/ncomms10438

**Published:** 2016-02-02

**Authors:** Susana Llanos, Juana M. García-Pedrero, Lucia Morgado-Palacin, Juan P. Rodrigo, Manuel Serrano

**Affiliations:** 1 Tumor Suppression Group, Department of Molecular Oncology, Spanish National Cancer Research Centre (CNIO), 3 Melchor Fernandez Almagro Street, Madrid 28029, Spain; 2 Department of Otolaryngology, Hospital Universitario Central de Asturias and Instituto Universitario de Oncología del Principado de Asturias, Universidad de Oviedo, Oviedo 33006, Spain

## Abstract

The levels, regulation and prognostic value of p21 in head and neck squamous cell carcinomas (HNSCC) has been puzzling for years. Here, we report a new mechanism of regulation of p21 by the mTORC1/4E-BP1 pathway. We find that non-phosphorylated 4E-BP1 interacts with p21 and induces its degradation. Accordingly, hyper-activation of mTORC1 results in phosphorylation of 4E-BP1 and stabilization of p21. In HNSCC, p21 levels strongly correlate with mTORC1 activity but not with p53 status. Finally, clinical data indicate that HNSCC patients with p21 and phospho-S6-double-positive tumours present a better disease-specific survival. We conclude that over-activation of the mTORC1/4E-BP1/p21 pathway is a frequent and clinically relevant alteration in HNSCC.

## Abstract (web summary)

The molecular pathways involving p21 and mTORC1 are frequently deregulated in head and neck squamous cell carcinomas (HNSCC). Here, Llanos *et al*. report a mechanism that regulates p21 stability through the mTORC1/4E-BP1 pathway independently of p53, and show that the mechanism is prevalent in HNSCC.

## Graphical abstract

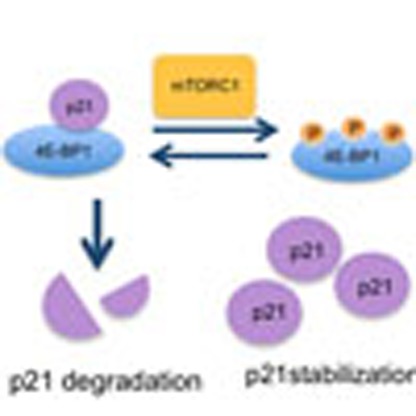

Progression through the cell cycle in eukaryotic cells is governed by a suite of cyclin-dependent kinases (CDKs) and CDK inhibitors. Protein CDKN1A (also known as p21), encoded by the *CDKN1A* gene, is a member of the CIP/KIP family of CDK inhibitors, together with CDKN1B (p27) and CDKN1C (p57). A main mechanism governing p21 levels is through *CDKN1A* transcriptional activation by the tumour-suppressor TP53 (p53). In response to DNA damage and many other cellular stressors, p21 levels increase in a p53-dependent manner and contribute to arrest cell proliferation. P21 also regulates multiple cellular processes, including apoptosis, differentiation and stem cell quiescence^1^,^2^. In addition to transcriptional upregulation by p53, other mechanisms have been described that regulate p21 levels^2^,^3^. Under normal growth conditions, p21 is an unstable protein with a relatively short half-life and several proteins involved in p21 degradation have been identified^2^.

The serine/threonine kinase MTOR (mTOR) promotes anabolic processes and cell growth in response to environmental cues. This is achieved through two distinct multiprotein complexes containing mTOR and known as mTORC1 and mTORC2 (refs 4, 5). The kinase activity of mTORC1 is positively regulated by two families of small GTPases, namely, RHEB and RRAGs (also known as RAGs)^6^. Of relevance for our current work, RHEB is negatively regulated by the GTPase-activating protein TSC2 (refs 6, 7, 8). The mTORC1 complex integrates inputs from at least four major signals: growth factors, energy status, oxygen and amino acids. When activated, mTORC1 promotes protein synthesis mainly by phosphorylating the kinases RPS6KB (also known as S6K) and the translational regulators EIF4EBP (also known as 4E-BP)^9^. Upon phosphorylation, 4E-BP releases the translation factor EIF4E (eIF4E) allowing it to promote the translation of a subset of mRNAs characterized by the presence of a terminal oligopyrimidine (TOP) track in the 5′-untranslated region (TOP mRNAs)^9^,^10^.

Head and neck squamous cell carcinoma (HNSCC) is the sixth leading cancer by incidence worldwide and is one of the most morbid, mortal and genetically diverse malignancies^11^. Despite improvements in treatment protocols, the survival rates remain disappointingly low. Insight in the molecular processes causing HNSCC may reveal the knowledge to improve and personalize treatments. The above-described molecular pathways involving p21 and mTORC1 are frequently deregulated in HNSCCs, but little is known about the possible connections between these two pathways. P21 is aberrantly expressed in the majority of HNSCC and its expression appears to be unrelated to p53 status^12^,^13^,^14^,^15^,^16^. On the other hand, alterations in the major components of the mTORC1 pathway are consistently observed in a large fraction of HNSCC cases^17^,^18^,^19^,^22^. Here, we have focused on the mechanistic connection between p21 and mTORC1. We dissect a mechanism that regulates p21 stability through the mTORC1/4E-BP1 pathway and independently of p53, and we show supportive evidence indicating that this mechanism is highly prevalent in HNSCC.

## Results

### Hyperactivation of mTORC1 elevates p21 independently of p53

We were initially motivated by the idea of studying mTOR-induced senescence in primary cells. Previous investigations had found that primary mouse embryo fibroblasts (MEFs) from knockout mice lacking the mTORC1 inhibitor TSC2 enter senescence prematurely in association with a remarkable upregulation of p21 (ref. 23). Cellular senescence is a tumour-suppressor mechanisms activated in response to multiple cellular stresses, prominently including oncogenic stimuli^24^. In this regard, cellular senescence can be considered a read-out of oncogenic stress in primary cells. In contrast, the senescence response is generally absent or impaired in cancer cells.

**Figure 1:**
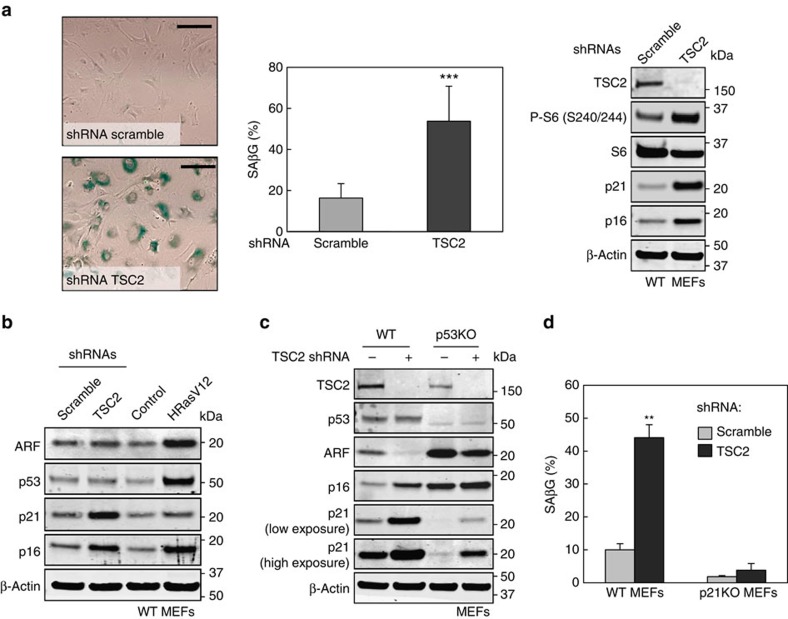
mTORC1 activation promotes senescence in MEFs concomitant with p53-independent induction of p21. (**a**) Representative images illustrating β-galactosidase activity in MEFs infected with scramble or TSC2 shRNAs (top left panel). Scale bar, 100 μm. Average quantification of four (*n*=4) independent experiments±s.d. (bottom left panel). Statistical *t*-test analysis was performed to calculate significance (***P≤0.005). Western blotting analysis of protein levels in scramble and TSC2-depleted MEFs (right panel). (**b**) Western blot depicting changes in protein levels in primary fibroblasts following infection with TSC2 shRNA, HRasV12 or control viruses. (**c**) Western blot analysis of protein expression levels in WT MEFs and p53KO MEFs infected with scramble or TSC2 shRNAs lentiviruses. (**d**) β-Galactosidase activity in WT and p21KO MEFs following infection with scramble or TSC2 shRNAs lentiviruses. Data corresponds to the average of two (*n*=2) independent infections±s.d. Statistical *t*-test analysis was performed to calculate significance (**P≤0.05). For each panel, all the western blots correspond to samples from the same experiment; in some cases, samples were distributed in several electrophoretic gels run in parallel.

In agreement with a previous report^23^, we confirmed that TSC2 depletion in primary MEFs, by infection with TSC2 short hairpin RNA (shRNA)-encoding lentiviruses, induced a robust senescence response (Fig. 1a). As expected, TSC2 depletion resulted in constitutive activation of mTORC1, as indicated by hyper-phosphorylation of ribosomal protein RPS6 (S6; Fig. 1a). In addition, and similar to other examples of oncogenic stimuli-induced senescence^24^, TSC2 depletion resulted in increased levels of the cell cycle inhibitors p21 and p16 (encoded by *CDKN2A*; Fig. 1a,b). Similar results were obtained with a different TSC2-targeting shRNA (Supplementary Fig. 1a). Interestingly, TSC2-depleted senescent MEFs did not display increased levels of alternative reading frame (ARF) (also encoded by *CDKN2A*) or p53 (Fig. 1b), which are critical mediators of oncogenic H-Ras (H-RasV12)-induced senescence^25^,^26^. Also, shTSC2-treated cells did not show evidence of DNA damage, as indicated by the absence of phosphorylated histone H2AFX (H2AX) and lack of Ser15-phosphorylated p53 (Supplementary Fig. 1b, c). These observations are in agreement with a previous study showing that mTORC1 hyper-activation via PTEN knockout results in cellular senescence independently of replicative damage and ARF^27^.

The cell cycle inhibitor p21 is a main transcriptional target of p53 and its cellular levels are usually associated with p53 activity. Despite our lack of evidence of p53 activation by shTSC2, we tested whether p21 upregulation was mediated by p53. For this, we silenced endogenous TSC2 in *p53*-null (p53KO) MEFs by lentiviral expression of TSC2 shRNAs and p21 upregulation was still clearly observed despite its lower basal levels (Fig. 1c). Similar results were obtained with a different TSC2 shRNAs (Supplementary Fig. 1d). TSC2 knockdown did not induce senescence in p53KO cells (Supplementary Fig. 1e) probably because p21 levels in these cells were not sufficiently high. To evaluate the relevance of p21 in mTORC1-induced senescence, we used *p21*-null (p21KO) MEFs and we observed that senescence was notably reduced compared with wt MEFs (Fig. 1d).

Taken together, these observations indicate that shTSC2-induced senescence in MEFs depends on p53 and p21. Also, p21 expression is determined by two factors: p53 sustains high basal levels of p21; and, independently of p53, shTSC2 upregulates p21. It is important to remark that the upregulation of p21 by mTORC1 is not secondary to senescence, as exemplified in p53KO MEFs. We hypothesize that there is a direct mechanistic link between mTORC1 and p21.

### mTORC1 stabilizes p21 protein

Next, we aimed to uncover the mechanisms responsible for mTORC1-mediated induction of p21. TSC2 depletion did not significantly upregulate the levels of *p21* mRNA in wild-type (WT) and p53KO MEFs (Supplementary Fig. 2). Therefore, although we cannot exclude a partial contribution of transcriptional regulation by p53 or by other factors, post-transcriptional mechanisms must be involved in the elevation of p21 protein levels upon mTORC1 hyperactivation.

**Figure 2:**
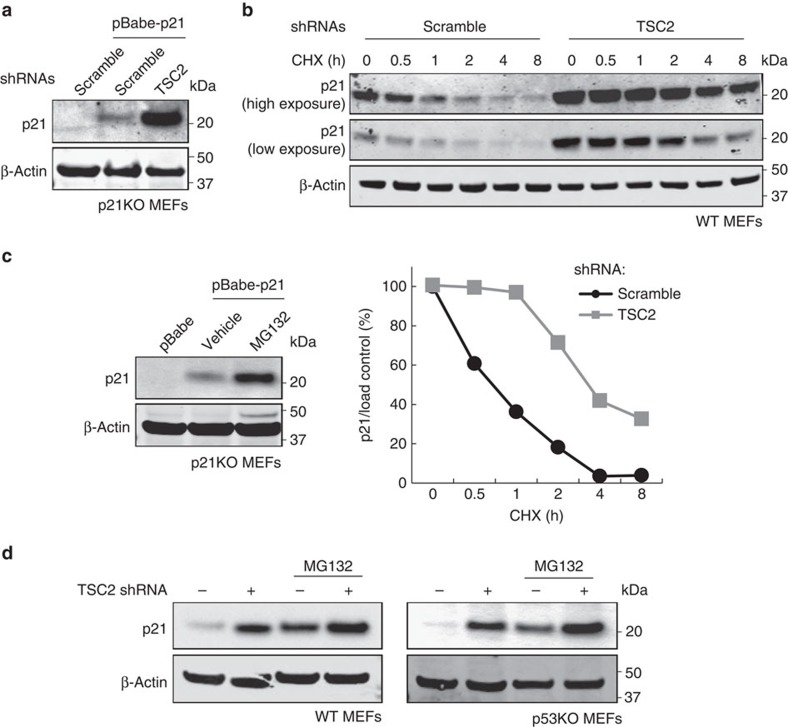
TSC2 depletion upregulates exogenous p21 levels and increases p21 half-life. (**a**) Representative western blot showing p21 levels in MEFs co-infected with pBabe-p21 retrovirus and either control or TSC2 shRNAs retroviruses. (**b**) Western blot analysis of p21 levels in scramble or TSC2 shRNA-expressing MEFs following incubation with 10 μM cyclohexamide (CHX) for the indicated times (top). Densitometric quantification of p21 levels corrected by loading control (bottom). (**c**) Western blot showing exogenous mouse p21 levels in the presence or absence of the proteasomal inhibitor MG132. (**d**) WT and p53KO MEFs were infected with either scramble or TSC2 shRNAs lentiviruses. Following selection, the cells were incubated for 8 h in the presence or absence of the proteasome inhibitor MG132. The p21 immunoblot of p53KO MEFs (right panel) is overexposed compared with WT MEFs (left panel). For each panel, all the western blots correspond to samples from the same experiment; in some cases, samples were distributed in several electrophoretic gels run in parallel.

It is well known that mTORC1 preferentially promotes the translation of a subset of mRNAs characterized by TOP motifs at the 5′-untranslated region (5′UTR)^10^. We could not identify any obvious TOP motifs in the human or mouse *p21* 5′UTR. Nonetheless, to unequivocally address the role of translation initiation, we ectopically expressed mouse p21 in p21KO MEFs using a plasmid that contained the *p21* open reading frame preceded by a heterologous 5′UTR. Importantly, the levels of ectopically expressed mouse p21 increased upon activation of mTORC1 by shTSC2 (Fig. 2a). These observations indicate that the 5′UTR of *p21* mRNA is not required for the effects of mTORC1 on p21 protein levels, and suggest that mTORC1 may regulate the stability of p21 protein. To test this possibility, we analysed the kinetics of p21 degradation in the presence of the translation inhibitor cycloheximide. We observed that TSC2 silencing significantly extended p21 half-life in WT MEFs (Fig. 2b). Also, the proteasome inhibitor MG132 stabilized the levels of ectopic mouse p21 (Fig. 2c). Finally, under conditions in which p21 protein does not undergo proteasomal degradation (that is, in the presence of MG132), p21 levels increased only modestly upon TSC2 knockdown (Fig. 2d). In conclusion, although we cannot exclude the participation of other mechanisms, such as transcription or translation, we conclude that protein stabilization accounts for most of the upregulation of p21 upon mTORC1 hyperactivation.

### Phosphorylation of 4E-BP1 stabilizes p21 protein

Constitutive activation of mTORC1 is known to shut down the PI3K/AKT signalling cascade through various negative feed-back loop mechanisms^4^ and we confirmed that this is the case in TSC2-depleted MEFs since phosphorylation of AKT and FOXO decreased upon TSC2 knockdown (Supplementary Fig. 3a). We wondered whether the inhibition of PI3K could be involved in the stabilization of p21. To test this, we treated cells with three different PI3K chemical inhibitors. All three inhibitors efficiently blunted AKT phosphorylation, but failed to increase p21 levels, thus indicating that p21 regulation was not a consequence of PI3K inhibition (Supplementary Fig. 3b).

**Figure 3:**
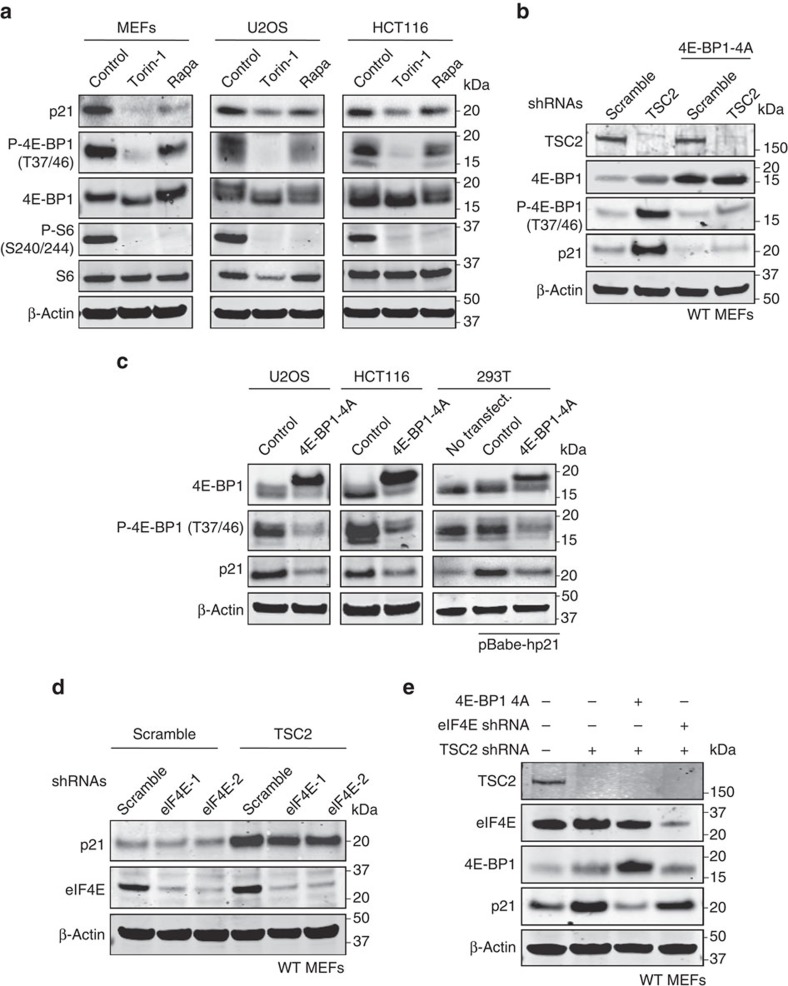
mTORC1-mediated upregulation of p21 requires 4E-BP1 phosphorylation. (**a**) Effect of mTOR inhibitors torin-1 (250 nM) and rapamycin (100 nM) on p21 levels in different cell lines. Cells were treated for 24 h with the indicated drugs and protein levels were subsequently analysed by western blot. (**b**) Protein levels in MEFs infected simultaneously with lentiviruses expressing either scramble or TSC2 shRNAs together with control or 4E-BP1-4A. (**c**) Protein levels in U2OS, HCT116 and 293T cells transfected with 4E-BP1-4A mutant alone (U2OS and HCT116 cells) or in combination with pBabe-p21 plasmid (293T cells). (**d**) Western blot depicting protein levels in MEFs following co-infection with lentiviruses expressing the indicated shRNAs. (**e**) Primary MEFs were infected with lentiviruses expressing scramble, TSC2 or eIF4E shRNAs in combination with control or 4E-BP1-4A. Protein levels were measured by immunoblotting. For each panel, all the western blots correspond to samples from the same experiment; in some cases, samples were distributed in several electrophoretic gels run in parallel.

Then, we focused on mTORC1 and its proximal downstream effectors and we obtained a clue from the differential effect of mTORC1 inhibitors torin-1 and rapamycin on p21 levels^28^,^29^. Torin-1, an ATP-competitive mTOR inhibitor, efficiently inhibited the phosphorylation of S6 (which reflects S6K activity) and 4E-BP1, and profoundly decreased p21 levels (Fig. 3a). In contrast, rapamycin, an mTORC1 allosteric inhibitor, efficiently inhibited S6 phosphorylation without affecting 4E-BP1 phosphorylation and, interestingly, the effect on p21 levels was modest (Fig. 3a). In light of these obervations, we hypothesized that p21 could be a downstream effector of 4E-BP1. In support of this, a 4E-BP1 phosphorylation-defective mutant (with mTORC1 phosphorylation sites Thr37, Thr46, Ser65 and Thr70 replaced by alanines, abbreviated as 4E-BP1-4A) blunted p21 upregulation by shTSC2 (Fig. 3b). Similar results were observed in p53KO MEFs (Supplementary Fig. 3c) and in p21KO MEFs expressing ectopic mouse p21 (Supplementary Fig. 3d). Furthermore, mutant 4E-BP1-4A downregulated endogenous p21 in human cancer cell lines U2OS and HCT116, and also downregulated ectopic human p21 in 293T cells (Fig. 3c). Finally, we directly confirmed that mutant 4E-BP1-4A decreases the half-life of endogenous p21 in HCT116 cells (Supplementary Fig. 3e). These observations imply that phosphorylation of 4E-BP1 by mTORC1 is key for the stabilization of p21.

Phosphorylation of 4E-BP1 disrupts the complex between 4E-BP1 and the translation factor eIF4E, releasing free active eIF4E^9^. Accordingly, phosphorylation-defective mutant 4E-BP1-4A associates efficiently to eIF4E (Supplementary Fig. 3f). To test the involvement of eIF4E in p21 regulation, we used two different shRNAs that profoundly downregulated eIF4E protein. Interestingly, inhibition of eIF4E only modestly affected the basal levels of p21 (Fig. 3d), and failed to prevent the upregulation of p21 by mTORC1 hyper-activation (Fig. 3e). We conclude that p21 stabilization by mTORC1 requires the phosphorylation of 4E-BP1 and it is independent of eIF4E.

### Non-phosphorylated 4E-BP1 interacts with p21

**Figure 4:**
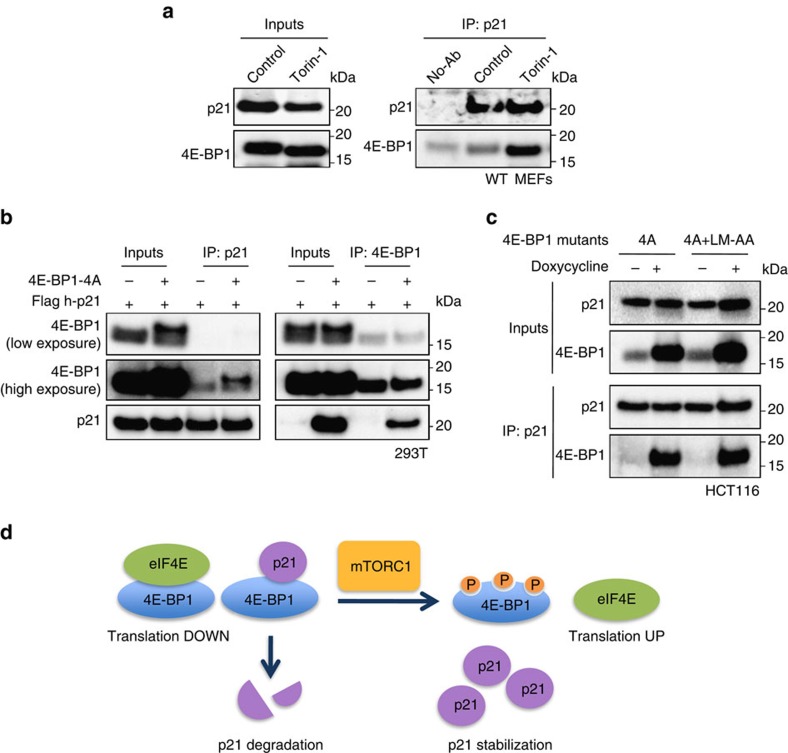
p21 interacts with non-phosphorylated 4E-BP1. (**a**) p21 was immunoprecipitated from WT MEFs untreated of treated with torin-1 (250 nM) for 6 hours in the presence of MG132 (25 μM). Inputs and co-inmunoprecipitated proteins were analysed by western blot. (**b**) 293T cells were transfected with 4E-BP1-4A and flag-p21 plasmids. Cell lysates were immunoprecipitated with either anti-4E-BP1 or anti-flag antibodies following incubation with MG132 (25 μM) for 5 h. Inputs and co-immunoprecipitated proteins were analysed by western blot. (**c**) HCT116-expressing Tet-On-inducible 4E-BP1 mutants were treated with doxycycline (1 μg ml^−1^) for 24 h. Cell lysates were immunoprecipitated with anti-p21 antibodies and subjected to immunoblot analysis. (**d**) Model for mTORC1/4E-BP1-mediated regulation of p21: Non-phosphorylated 4E-BP1 binds to p21 and promotes p21 degradation. 4E-BP1 phosphorylation by mTORC1 disrupt the 4E-BP1/p21 complex ensuing p21 accumulation. 4E-BP1 regulates p21 independently of its interaction with the the translation initiation factor eIF4E. For each panel, all the western blots correspond to samples from the same experiment; in some cases, samples were distributed in several electrophoretic gels run in parallel.

Having excluded a role for eIF4E, we wondered whether p21 regulation could be the result of the interaction between p21 and 4E-BP1. We envisaged two alternative possibilities: either binding to phosphorylated 4E-BP1 results in p21 stabilization or binding to non-phosphorylated 4E-BP1 promotes p21 degradation. We addressed these possibilities by a series of immunoprecipation assays, which were all carried out in the presence of the proteasomal inhibitor MG132 to prevent p21 degradation. Interestingly, we found that endogenous p21 co-immunoprecipitated with endogenous 4E-BP1 only in the presence of torin-1, when 4E-BP1 phosphorylation by mTORC1 is completely blunted (Fig. 4a). Similar results were obtained in human cell lines U2OS and HCT116 by co-immunoprecipitating endogenous proteins (Supplementary Fig. 4a), and in 293T cells expressing exogenous human p21 (Supplementary Fig. 4b). Of note, the mTORC1 inhibitor rapamycin, which failed to reduce phosphorylated 4E-BP1 efficiently, did not promote the 4E-BP1/p21 interaction (Supplementary Fig. 4a,b). To directly prove the interaction of p21 with non-phosphorylated 4E-BP1, we transfected cells with plasmids encoding 4E-BP1-4A and human p21. Importantly, complex formation was readily detected by immunoprecipitation of either one of the two proteins in 293T cells (Fig. 4b). The interaction between p21 and 4E-BP1-4A was also demonstrated in p53-defective HCT116 cells (Supplementary Fig. 4c) and in TSC2-depleted MEFs (Supplementary Fig. 4d). Interestingly, eIF4E efficiently bound to endogenous 4E-BP1 and mutant 4E-BP1-4A, but it did not associate with p21 even in the presence of 4E-BP1-4A (Supplementary Fig. 4e). We conclude that p21 and eIF4E form separate complexes with non-phosphorylated 4E-BP1.

To further prove that the 4E-BP1/p21 complex was independent of eIF4E, we generated an eIF4E-binding-defective 4E-BP1 mutant by substituting residues Leu59 and Met60 of 4E-BP1-4A to Ala^30^, and this mutant (4E-BP1-4A+LM-AA) was introduced into HCT116 cells under a doxycycline-inducible promoter. As expected, 4E-BP1-4A+LM-AA double mutant failed to associate to eIF4E (Supplementary Fig. 4f) but still retained the ability to interact with p21 (Fig. 4c).

Taken together, these results are consistent with a model in which, under basal conditions, non-phosphorylated 4E-BP1 associates with p21 and promotes its degradation. In contrast, upon mTORC1 activation, the 4E-BP1/p21 complex is disrupted, and p21 is stabilized (Fig. 4d).

### p21 expression correlates with mTORC1 activity in HNSCC

**Figure 5:**
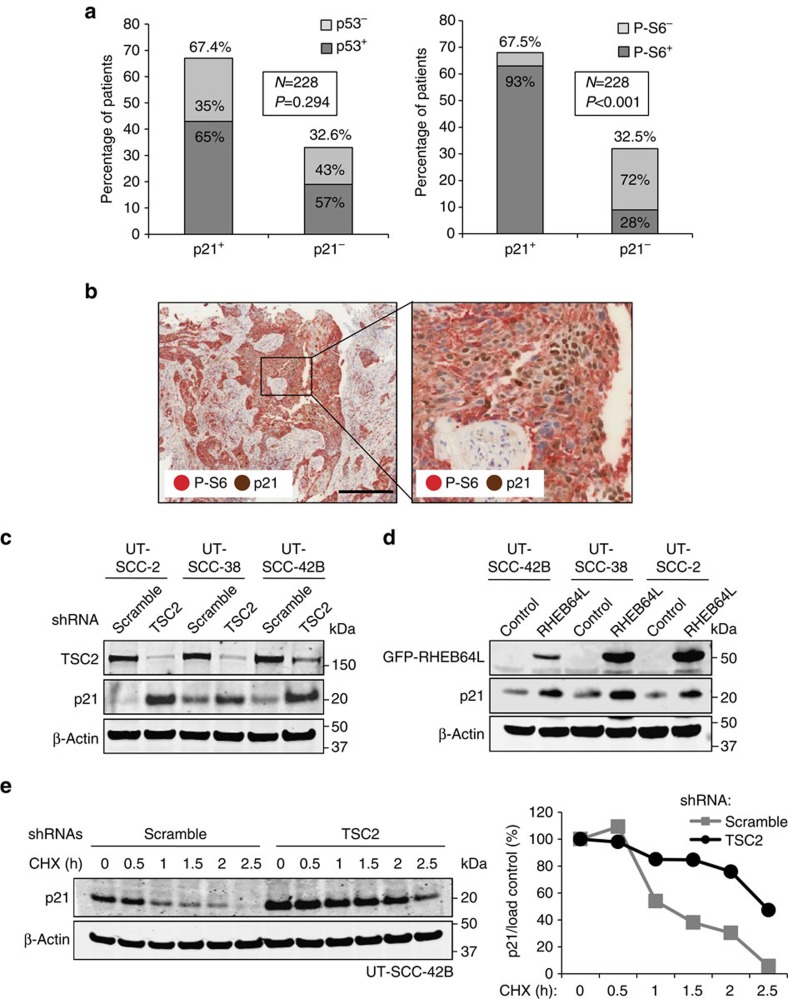
mTORC1/4E-BP1 regulates p21 expression in HNSCC. (**a**) Graphical representation of the IHC data to correlate p21 and p53 expression (left) or p21 and P-S6 expression (S240/244; right; see also Supplementary Tables 2 and 3). Statistical significance was calculated using the Fisher exact test (two-sided). (**b**) Representative sections from HNSCC tissue samples depicting the IHC detection of p21 and P-S6 (S240/244). Scale bar, 200 μm. (**c**) Western blot analysis of p21 and TSC2 protein levels in HNSCC cell lines infected with scramble or TSC2 shRNAs lentiviruses. (**d**) Western blot analysis of p21 protein levels in HCT116 cells co-transfected with p21-flag- and Rheb64L-GFP-expressing plasmids. (**e**) Western blot analysis of p21 levels in scramble or TSC2 shRNA-expressing UT-SCC-42B cells following incubation with 10 μM cyclohexamide (CHX) for the indicated times (left). Densitometric quantification of p21 levels corrected by the loading control (right). For each panel, all the western blots correspond to samples from the same experiment; in some cases, samples were distributed in several electrophoretic gels run in parallel.

The levels of p21 correlate well with p53 status in most cancer types. However, this is not the case in HNSCC where p21 levels seem unrelated to the status of p53 (refs 12, 13, 14, 15). On the other hand, the mTORC1 pathway is frequently hyperactive in HNSCC^11^,^17^,^18^,^19^,^20^,^21^,^22^. These two sets of observations, together with our current mechanistic findings, made us wonder whether p21 levels could be governed by the mTORC1 pathway in HNSCC. To test this hypothesis, we analysed p21, p53 and P-S6 protein levels by immunohistochemistry (IHC) in a large series (*n*=274) of paraffin-embedded tumour samples from surgically treated HNSCC patients. Of note, to decrease the heterogeneity of the tumour set, we only studied HPV-negative HNSCC all of them located in the oropharynx, larynx and hypopharynx (Supplementary Table 1). Positive p53 immunohistochemical staining was interpreted as evidence of mutant p53, and positive P-S6 was used as a surrogate for mTORC1 activity. In agreement with previous reports^12^,^13^,^14^,^15^, p21 expression showed no association with p53 functional status in this cohort (Fig. 5a and Supplementary Table 2). In contrast, a strong positive correlation was found between p21 and P-S6 (Fig. 5b and Supplementary Table 3). These observations are consistent with a causal relationship between mTORC1 activity and p21 levels in HNSCC. To further support this notion, TSC2 knockdown markedly induced endogenous p21 protein in three different human HNSCC cell lines derived from primary tumours (Fig. 5c). Moreover, overexpression of a constitutively active RHEB mutant also increased the levels of endogenous p21 (Fig. 5d) and ectopic human p21 (Supplementary Fig. 5a). Finally, TSC2 knockdown increased p21 protein half-life in UT-SCC-42B cells (Fig. 5e). Taken together, these observations indicate that the mTORC1/4E-BP1/p21 regulatory axis is active in HNSCC both *in vivo* and *in vitro*. mTORC1-mediated p21 upregulation, did not impact in the phosphorylation status of the retinoblastoma protein RB1 (Supplementary Fig. 5b), nor resulted in senescence in HNSCC cell lines (Supplementary Fig. 5c). Since *p53*-null MEFs failed to undergo senescence upon mTORC1 hyperactivation (Supplementary Fig. 1e), we wondered if the HNSCC cells used here could lack functional p53. Indeed, the three HNSCC cell lines were proven to have deleterious mutations in the *p53* gene, as it was previously reported for UT-SCC-2 (ref. 30) or as determined by us in the case of the UT-SCC-38 and UT-SCC-42B (Supplementary Fig. 5d). The absence of functional p53 protein was further confirmed by immunoblot and by the failure of the MDM2 inhibitor nutlin-3 to stabilize p53 (Supplementary Fig. 5e). In addition to p53 inactivation, other alterations, including CCND1 (cyclin D1) overexpression, or loss of p16, which are common events in HNSCC^11^,^32^, could explain the resistance of these cells to undergo senescence upon mTORC1 hyperactivation and p21 upregulation. Notwithstanding, p21 upregulation by mTORC1 in cancer cells may impinge in multiple other cellular processes of clinical relevance, including apoptosis, differentiation and stem cell quiescence^1^,^2^.

### p21 and P-S6 predict survival of HNSCC patients

**Figure 6:**
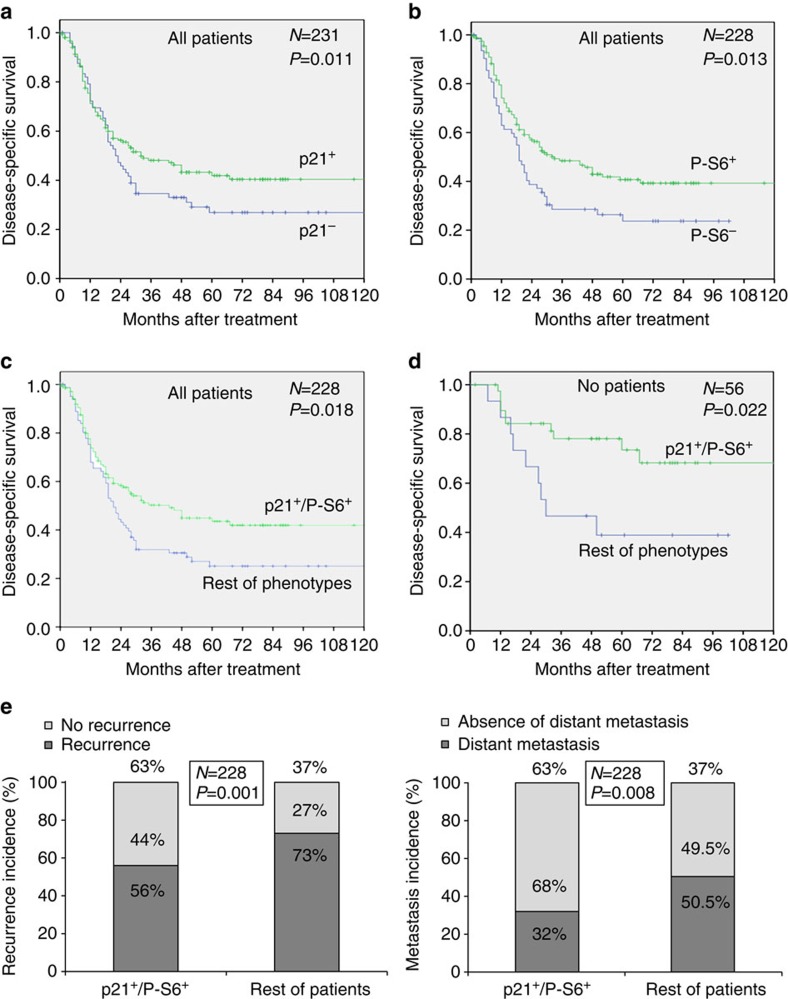
p21 and P-S6 protein expression correlates with improved survival in HNSCC patients. (**a**) Kaplan–Meier disease-specific survival curves categorized by p21. (**b**) Kaplan–Meier disease-specific survival curves categorized by P-S6 (240/244). (**c**) Kaplan–Meier disease-specific survival curves categorized by p21 and P-S6 (240/244). (**d**) Kaplan–Meier disease-specific survival curves of patients without regional lymph node affection at the time of diagnosis (N0) categorized by p21 and P-S6 (240/244). (**e**) Graphical representation of the IHC data to correlate p21^+^/P-S6^+^-double-positive phenotype and disease recurrence (left) or distant metastasis (right) (see also Supplementary Tables 6 and 7). For **a**–**d**, statistical significance was calculated using the log-rank test. For **e**, statistical significance was calculated using the Fisher exact test (two-sided).

Next we examined the impact of both p21 and P-S6 protein expression on the clinical outcome of HNSCC patients. Interestingly, patients harbouring tumours positive for either p21 or P-S6 showed a significantly improved disease-specific survival (Fig. 6a,b). In agreement with the strong association between p21 and P-S6 expression, the combination of both molecular markers also associated with a good prognosis (Fig. 6c). However, in multivariate Cox analysis (including localization of the tumour, T classification, lymph node metastasis, pathological grade, and p21^+^/P-S6^+^ phenotype), we found that only the presence of regional lymph node metastasis at the time of diagnosis was a significant independent predictor of reduced disease-specific survival (Supplementary Table 4). Given the dominant prognostic value of lymph node metastasis, we examined the prognostic significance of p21^+^/P-S6^+^ phenotype in the subgroup of patients with no regional lymph node affection at the time of diagnosis (N0 patients). Indeed, the p21^+^/P-S6^+^ phenotype strongly associated with improved survival in N0 patients (Fig. 6d), but not in patients with affected lymph nodes (N+ patients; Supplementary Fig. 6). Therefore, hyper-activation of the mTORC1/4E-BP1/p21 axis could help to predict the prognosis of HNSCC patients with no lymph node affectation at the time of diagnosis.

In agreement with previous reports indicating that p21 expression in HNSCC is compatible with proliferation^13^,^14^,^32^, we did not find a statistical significant correlation between Ki67 staining and p21 expression (Supplementary Table 5) suggesting that these tumours carry alterations that override the cell cycle inhibitory activity of p21. Therefore, cell-cycle inhibition is unlikely responsible for the increased survival rate associated to p21-positive HNSCC patients. It is known that p21 is involved in multiple other cellular processes, including apoptosis, differentiation and stem cell quiescence that could explain the association of p21 with good clinical prognosis in HNSCC patients.^1^,^2^.

Interestingly, patients with p21/P-S6-double-positive tumours presented lower rates of recurrence (including local recurrences, regional recurrences and distant metastasis) and lower incidence of distant metastases (Fig. 6e and Supplementary Tables 6,7). A similar trend was observed when the analysis was restricted to the N0 patients (Supplementary Tables 8, 9). As recurrence is the main determinant factor for survival, these observations can explain the improved disease-specific survival associated to patients with p21/P-S6-double-positive HNSCC tumours.

## Discussion

In this paper, we describe a p53-independent mechanism of regulation of p21 via the mTORC1 kinase. Our results indicate that the mTORC1 substrate 4E-BP1 interacts with p21 in its non-phosphorylated state and promotes p21 destabilization. Accordingly, mTORC1-mediated phosphorylation of 4E-BP1 abrogates its binding to p21 resulting in p21 stabilization. Previous studies have reported that p21 levels are affected by mTORC1 through increased general mRNA translation^33^ and specific increased translation of *p53* (ref. 27) and *p21* (ref. 34) mRNAs. In addition, activation of mTORC1 inhibits MDM2 through S6K1-mediated phosphorylation^35^ and nucleolar sequestration^36^, leading to p53 activation and p21 upregulation. Here, we identify another layer of regulation by showing that mTORC1 can regulate p21 protein levels independently of p53, eIF4E and the 5′UTR sequence of *p21* mRNA, via phosphorylation of 4E-BP1 which disables the destabilization of p21 by unphosphorylated 4E-BP1.

In primary cells, the upregulation of p21 by mTORC1 results in senescence only in cells with active p53, but not in cells lacking p53. The induction of p21-dependent senescence could function as a barrier to prevent malignant progression of cells with a hyperactive mTORC1. This protective function, however, is abrogated in HNSCC probably because they carry multiple alterations that bypass or cancel senescence, such as inactivation of p53, overexpression of cyclin D1 or CCNE1 (cyclin E), or loss of p16, which are all common events in HNSCC^11^,^32^. Nonetheless, p21 is known to regulate a variety of cellular processes beyond the cell cycle^1^,^2^, and these other processes could be of clinical relevance.

In agreement with previous studies on HNSCC^12^,^13^,^14^,^15^, using a large series of 274 HNSCC tissue specimens, we found that a considerable proportion of tumours (67%) showed p21 expression irrespective of p53 status. In contrast, there was a strong significant correlation between p21 and P-S6 protein expression, thereby supporting a causal connection between mTORC1 activity and p21 levels in HNSCC. More importantly, when correlated with clinical data, we found that p21, P-S6 or the combination of both proteins significantly associated with disease-specific survival in our cohort of patients with orolaryngeal, laryngeal and hypopharyngeal HNSCC. This association was particularly strong in the case of HNSCC patients with no lymph node affectation at the time of diagnosis, which could be of clinical value. In line with our observations, other types of cancers, such as non-small cell lung cancer^37^,^38^ and luminal breast cancer^39^, also show a positive association between hyperactivation of mTOR and good clinical prognosis. In contrast, a negative association has been reported in other tumour types, such as oesophageal squamous cell carcinoma^40^ and nasopharyngeal carcinoma^41^.

In summary, our results demonstrate that the mTORC1/4E-BP1/p21 regulatory axis is relevant in HNSCC, and more importantly, it favourably affects clinical outcome. These observations are not in conflict with the oncogenic role of mTORC1 in these cancers, or with the potential therapeutic value of mTOR inhibitors for HNSCC. Future studies should uncover why HNSCC with a hyperactive mTORC1/4E-BP1/p21 pathway have a better prognosis than tumours with less active mTORC1.

## Methods

### Cells

Cell lines U2OS (human osteosarcoma), HCT116 (human colon carcinoma) and were obtained from American Type Culture Collection and were authenticated after completion of this work. Cell line authentication was performed by analysing a total of ten microsatellites at the authentication service (Genomic Unit) of the Institute of Biomedical Research ‘Alberto Sols' in Madrid. 293T cells were purchased from Invitrogen. Dr Reidar Grenman provided the HNSCC cell lines derived from primary lesions: UT-SCC-42B, UT-SCC-2 and UT-SCC-38. These cells have been previously described^42^,^43^,^44^,^45^. The 4E-BP1/2 KO MEFs were a gift from Dr Nahum Sonenberg. All cell lines were tested periodically for mycoplasma contamination using the mycoplasma tissue culture NI (MTC-NI) Rapid Detection System from GEN-PROBE.

To generate mutant 4E-BP1 inducible cells, HCT116 cells were infected with lentiviral tet-on plasmid pCW57.1 encoding the 4E-BP1 mutants 4E-BP1-4A and 4E-BP1-4A-LM-AA. The cells were selected with 2 μg ml^−1^ puromycin for 4 days and growth in selection thereafter. The expression of 4E-BP1 mutants was induced by adding 1 μg ml^−1^ doxycycline to the culture medium for 24–48 h.

All the cell lines were maintained in DMEM supplemented with 10% fetal bovine serum and antibiotic-antimicotic (all from Gibco), and incubated in 20% O_2_ and 5% CO_2_ at 37 °C. To grow HNSCC cell lines, the medium was also supplemented with non-essential amino acids (Sigma).

### Reagents

pLESIP 4E-BP1-4A mutant lentiviral plasmid in which Thr37, Thr46, Ser65 and Thr70 were replaced by Ala was a kind gift from Dr Silvio Gutkind. The mutant 4E-BP1-4A+LM-AA was generated by site-directed mutagenesis using QuikChange II XL Site-Directed Mutagenesis kit from Agilent Technologies (#200521). The pCW57.1-4E-BP1-4A Tet-On inducible lentiviral plasmid was generated in the laboratory of Dr David Sabatini and was obtained from Addgene (#38240). pBabe-puro-human-p21 was generated in the laboratory of Gordon Peters and pBabe-puro-mouse-p21 was kindly donated by Mariano Barbacid. The pMT5-Flag-p21 (human) plasmid was acquired from Addgene (#16240). The pLJM1-EGFP-Rheb64L plasmid was kindly donated by David Sabatini.

The low-molecular-weight compound CNIO-PI3Ki is covered by patent WO2010/119264 (files available at the World Intellectual Property Organization, http://www.wipo.int/pctdb/en/wo.jsp?WO=2010119264). This is a potent inhibitor of PI3K isoforms p110α (Ki=2.4 nM) and p110δ (Ki=9.8 nM; inhibition of the other PI3K isoforms, p110β and p110γ, has values of Ki>100 nM, and inhibition of a total of 282 additional kinases including mTOR and DNAPK requires concentrations of IC_50_>1 μM). The small PI3K inhibitor GDC-0941 was obtained from Shanghai Haoyuan Chemexpress, and LY-294002 was obtained from CalBiochem. Torin-1 was obtained from Tocris. Rapamycin, nutlin3, cyclohexamide (CHX) and MG132 (proteasome inhibitor) were obtained from Sigma. Doxorubicin was purchased to Pharmacia and Upjohn.

### Antibodies

The following antibodies were used for western blot and immunoprecipitation: anti-p16 (M-156) from Santa Cruz, anti-human p53 (DO-1) from Santa Cruz, anti-p53 (1C12) from Cell Signaling, anti-phospho-p53 (Ser15) from Cell Signaling (#9284), anti-p19/ARF (5-C3-1) from Santa Cruz, anti-γH2A.X (Ser139) (clone JBW30) from Millipore, rabbit and goat anti-p21 (C-19) from Santa Cruz, anti-Tuberin/TSC2 from Cell Signaling (#3612), anti-4E-PB1 (53H11) from Cell Signaling, anti-phospho-4E-BP1 (37/46) (236B4) from Cell Signaling, anti-eIF4E (clone #299910) from R&D Systems, anti-phospho-S6 (Ser240/244) from Cell Signaling (#2215), anti-S6 (5G10) from Cell Signaling, anti-phospho-AKT (Ser473) (193H12), anti-phospho-AKT (Thr308) from Cell Signaling (#9275), anti-AKT from Millipore (07-416), anti-phospho-FOXO1(T24)/3a(T32), anti-FOXO1 (C29H4) from Cell Signaling, anti-flag M2 from Agilent anti-phospho-RB1 (Ser807/811) (D20B12) from Cell Signaling (#8516), anti-RHEB (C-19) from Santa Cruz.

For IHC, the following antibodies were used: rabbit monoclonal Phospho-S6 Ribosomal Protein (Ser240/244; D68F8 XP; Cell Signaling # 5364) at 1:200 dilution; Novocastra Liquid mouse monoclonal antibody p21WAF1 Protein (Clone 4D10; Leica Biosystems NCL-L-WAF-1) at 1:10 dilution; mouse monoclonal anti-human p53 Protein (Clone DO-7; Dako # M 7001) and Ki67 (Dako, clon MIB-1, prediluted).

### Senescence assay

MEFs were infected with scramble or TSC2 shRNA lentivirus and selected with puromycin for 4 days. 10–14 days after infection, β-galactosidase activity was assessed with a β-Galactosidase Staining Kit (Cell Signaling #9860). In each experiment, β-Galactosidase-positive and -negative cells were counted in at least ten different fields. Statistical significance was analysed by a Student's two-side *t*-test. Data represent the mean±s.d. of independent experiments.

### RNA interference

Silencing of endogenous proteins was performed using pLKO.1 lentiviral plasmids encoding specific shRNA sequences. All the lentiviral shRNA plasmids were purchased from Sigma: mouse TSC2-1 shRNA (TRCN0000042723), mouse TSC2-1 shRNA (TRCN0000042724), human TSC2 shRNA (TRCN0000040178), mouse eIF4E-1 shRNA (TRCN0000077475), mouse eIF4E-2 shRNA (TRCN0000077477). Control plasmid pLKO.1 scramble shRNA was acquired from Addgene (plasmid 1864). Infective lentiviral particles were produced according to the manufacturer's instructions. Briefly, HEK293FT cells (Invitrogen) were co-transfected with shRNA vectors and the lentivirus packaging plasmids PLP1, PLP2 and PLP-VSVG. The lentiviral supernatants were collected 48 h after transfection, filtered and used to infect the cell lines of interest. Following infection, the cells were selected for 3–4 days with 2 μg ml^−1^ puromycin.

### Protein analyses

The western blotting procedure has been previously described^46^. Primary antibodies (at a 1:1,000 dilution) were detected with secondary antibodies labelled with Alexa Fluor 680 (Molecular Probes; at a 1:5,000 dilution) and were visualized using a LI-COR Odyssey scanner. When Trueblot horseradish peroxidase-conjugated secondary antibodies (Rockland) were used (at a 1:1,000 dilution), chemiluminescence was detected with a Molecular Imager Gel Doc XR System (Bio-Rad). Proteins with similar or identical molecular weight (for example, phosphorylated and non-phosphorylated) were blotted in independent and equivalent gels. For protein half-life assays, cells were treated with 10 μM CHX during the indicated times.

For immunoprecipitation, cells were treated with 20 μM MG132 for 4–6 h and then lysed in lysis buffer (50 mM Tris pH8, 1 mM EDTA, 120 mM NaCl and 1% NP40) containing phosphatase inhibitors (0.25 mM Na_3_VO_4_, 10 mM NaF, 1 mM phenylmethylsulphonyl fluoride) and supplemented with a protease inhibitor cocktail (Roche). Cell lysates (2–4 mg) were incubated with the immunoprecipitating antibody (1 μg) along with either Trueblot anti-rabbit IgG IP beads (Rockland) or protein-G Sepharose beads (GE Helthcare) at 4 °C overnight. Beads were washed four times with lysis buffer, resuspended in 2 × Laemmli sample buffer and resolved by western blotting. For immunoprecipitation, the following antibodies were used: goat anti-p21 antibody (C-19 Santa Cruz), rabbit anti-4E-BP1 (53H11, Cell Signaling) and rabbit anti-eIF4E antibody (clone #299910, R&D Systems). To prevent hindrance by the immunoprecipitated IgG light-chain proteins, IgG Trueblot secondary antibodies (Rockland) were used. In addition, as p21 and 4E-BP1 proteins exhibit similar molecular weights and in order to avoid interference of both signals, these co-immunoprecipitated proteins were resolved in two independent western blots. Uncropped images of all the inmunoblots can be found in Supplementary Note 1.

### Quantitative real-time PCR

Total RNA was isolated with Trizol reagent (Invitrogen). cDNA synthesis was performed with 5 mg total RNA using Ready-To-Go You-Prime First-Strand Beads (GE Healthcare). Quantitative real-time PCR was carried out with SybrGreen Master Mix (Applied Byosystems) in a 7500 Fast Real-Time PCR System (Applied Byosystems). Reactions were performed in triplicate and normalized by β-acting expression. Primer sequences are depicted below:

CDKN1A forward: 5′-GTGGGTCTGACTCCAGCCC-3′;

CDKN1A reverse: 5′-CCTTCTCGTGAGACGCTTAC-3′;

β-actin forward: 5′-GGCACCACACCTTCTACAATG-3′;

β-actin reverse: 5′-GTGGTGGTGAAGCTGTAGCC-3′.

### Patients and tissue specimens

Surgical tissue specimens from 274 patients with HNSCC who underwent resection of their tumours at the ‘Hospital Universitario Central de Asturias' between 2000 and 2009 were collected, in accordance to approved institutional review board guidelines. All experimental protocols were approved by the Institutional Ethics Committee of the Hospital Universitario Central de Asturias and by the Regional CEIC from Principado de Asturias for the project PI13/00259. Written informed consent was obtained from all patients. Representative tissue sections were obtained from archival paraffin-embedded blocks and an experienced pathologist confirmed the histological diagnosis. All patients had a single primary tumour, microscopically clear surgical margins and received no treatment before surgery. Only 12 patients were women, and the mean age was 59 years (range 36–86 years; see Supplementary Table 1). All but 10 patients were habitual tobacco smokers, 164 moderate (1–50 pack-year) and 100 heavy (>50 pack-year), and 241 were alcohol drinkers. The stage of the tumours was classified according to 7th edition of the Tumour-nodes-metastasis system of the Union for International Cancer Control: 18 tumours were stage I, 14 were stage II, 42 were stage III and 200 were stage IV. According to Broders' classification, the series included 100 well, 110 moderately and 64 poorly differentiated tumours. None of the patients presented distant metastasis at the time of diagnosis. After surgery, and in function of the tumour stage, 164 (60%) patients received postoperative radiotherapy. HPV status was available for the whole series^47^,^48^. To improve sample uniformity, all HPV-positive cases (n=10) were excluded from the analysis.

### Tissue arrays

Morphologically representative areas were selected from each individual tumour paraffin block: three 1 mm cylinders were taken to construct tissue array blocks as described previously^49^ containing a total of 274 HNSCC (141 oropharyngeal, 65 hypopharyngeal, 68 laryngeal carcinomas). Briefly, the original archived haematoxylin-and-eosin-stained slides were reviewed by a pathologist and 1 mm diameter tissue cores from donor blocks were transferred to a recipient ‘Master' block in a grid-like manner using a manual tissue array instrument. Three tissue cores were taken from the donor tissue blocks to fully represent each case. In addition, each tissue array included three cores of normal epithelium as an internal negative control. To check the histopathological diagnosis and the adequacy of tissue sampling, a section from each tissue array was stained with haematoxylin and eosin and examined using light microscopy.

### Immunohistochemistry

The formalin-fixed, paraffin-embedded tissues HNSCC TMAs were cut into 3-μm sections and dried on Flex IHC microscope slides (Dako). The sections were de-paraffinized with standard xylene and hydrated through graded alcohols into water. Antigen retrieval was performed using Envision Flex Target Retrieval solution, high pH (Dako). Staining was done at room temperature on an automatic staining workstation (Dako Autostainer Plus) using the Dako EnVision Flex+Visualization System (Dako Autostainer) and the antibodies previously described. Immunostaining was scored blinded to clinical data by two independent observers. Staining data were dichotomized as negative expression (0–10% stained cells) versus positive expression (>10% stained cells).

### Genomic p53 sequencing

Primers and protocols used to sequence genomic p53 from HNSCC cell lines were found at the IARC TP53 Database: http://p53.iarc.fr.

### Statistical analysis

Survival curves were analyzed using the Kaplan–Meier product limit estimate. Differences between survival times were analysed by the log-rank method. Fisher's test was used to analyse two-by-two tables. For continuous variables, we used the Student's t test. Cox proportional hazard models were used to examine the relative impact of those statistically significant variables in univariate analysis. *P* values of ≤0.05 were considered to be statistically significant.

## Additional information

**How to cite this article:** Llanos, S. *et al.* Stabilization of p21 by mTORC1/4E-BP1 predicts clinical outcome of head and neck cancers. *Nat. Commun.* 7:10438 doi: 10.1038/ncomms10438 (2016).

## Supplementary Material

Supplementary Information Supplementary Figures 1-6, Supplementary Tables 1-9 and Supplementary Note 1.
