# Supplementary material for: Stabilization of p21 by mTORC1/4E-BP1 predicts clinical outcome of head and neck cancers
Source: Nat Commun. 2016 Feb 2;7:10438. doi: 10.1038/ncomms10438 (PMC4740818; doi:10.1038/ncomms10438)
Supplement: Supplementary Information — Supplementary Figures 1-6, Supplementary Tables 1-9 and Supplementary Note 1. [file ncomms10438-s1.pdf]

Supplementary Fig. 1

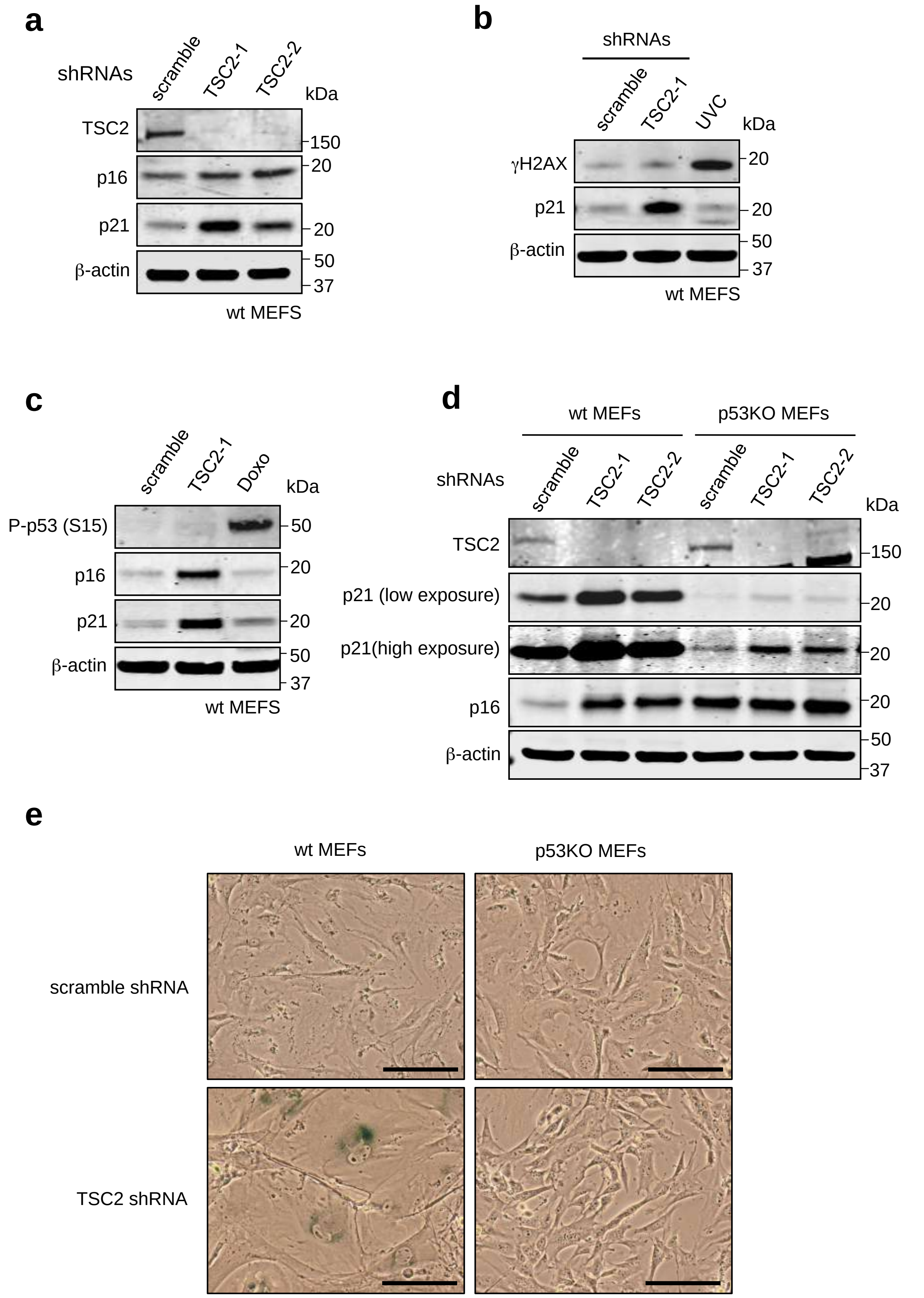

Supplementary Figure 1

(a) Western blot showing protein levels in wt MEFs infected with lentiviruses encoding either scramble or two different TSC2 shRNA sequences (TSC2-1 and TSC2-2).

(b) Western blot analysis of protein levels in wt MEFs following UVC irradiation (1J / cm^2) or TSC2-depletion by lentiviral infection. UVC-irradiated cells were lysed 30 min after irradiation.

(c) Western blot analysis of protein levels in wt MEFs treated with Doxorubicin (1 μM/ 6 hours) or depleted of TSC2 by lentiviral infection.

(d) Western blot depicting the impact of TSC2 depletion on p21 and p16 protein levels in both wt and p53KO MEFs. Two different TSC2 shRNA sequences (TSC2-1 and TSC2-2) were used to knock-down the endogenous protein.

(e) Representative images illustrating β-galactosidase activity in MEFs infected with either scramble or TSC2 shRNA-encoding lentiviruses. Scale bar 100 μm.

For each panel, all the Western blots correspond to samples from the same experiment; in some cases, samples were distributed in several electrophoretic gels run in parallel.

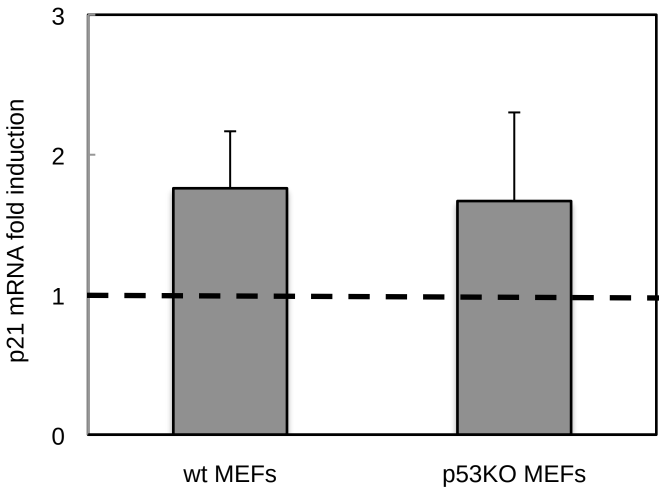

Supplementary Figure 2

qRT-PCR measurement of *p21* mRNA levels in wt and p53KO MEFs depleted of TSC2 by infection with TSC2 shRNAs lentiviruses. Fold changes in *p21* mRNA are relative to scramble shRNA-infected cells. The graph represents the average of four independent infections ± SD (n=4). No significance was found by statistical t-test analysis.

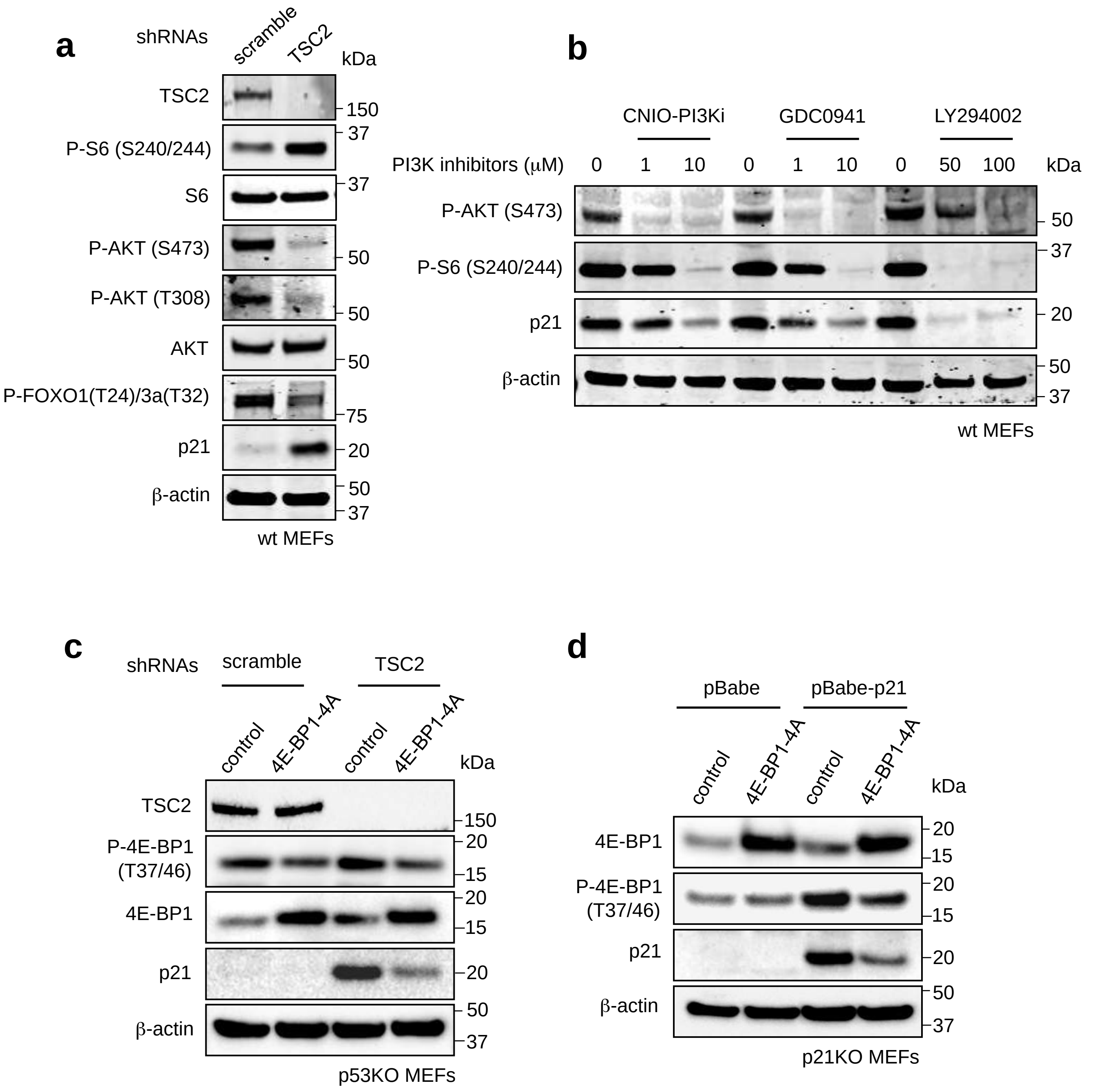

a
shRNAs
scramble
TSC2
kDa
TSC2
P-S6 (S240/244)
S6
P-AKT (S473)
P-AKT (T308)
AKT
P-FOXO1(T24)/3a(T32)
p21
β-actin
150
37
37
50
50
50
75
20
50
37
wt MEFs
b
CNIO-PI3Ki
GDC0941
LY294002
PI3K inhibitors (μM)
0 1 10 0 1 10 0 50 100
kDa
P-AKT (S473)
P-S6 (S240/244)
p21
β-actin
50
37
20
50
37
wt MEFs
c
shRNAs
scramble
TSC2
control
4E-BP1-4A
control
4E-BP1-4A
kDa
TSC2
P-4E-BP1 (T37/46)
4E-BP1
p21
β-actin
150
20
15
20
15
20
50
37
p53KO MEFs
d
pBabe
pBabe-p21
control
4E-BP1-4A
control
4E-BP1-4A
kDa
4E-BP1
P-4E-BP1 (T37/46)
p21
β-actin
20
15
20
15
20
50
37
p21KO MEFs

e

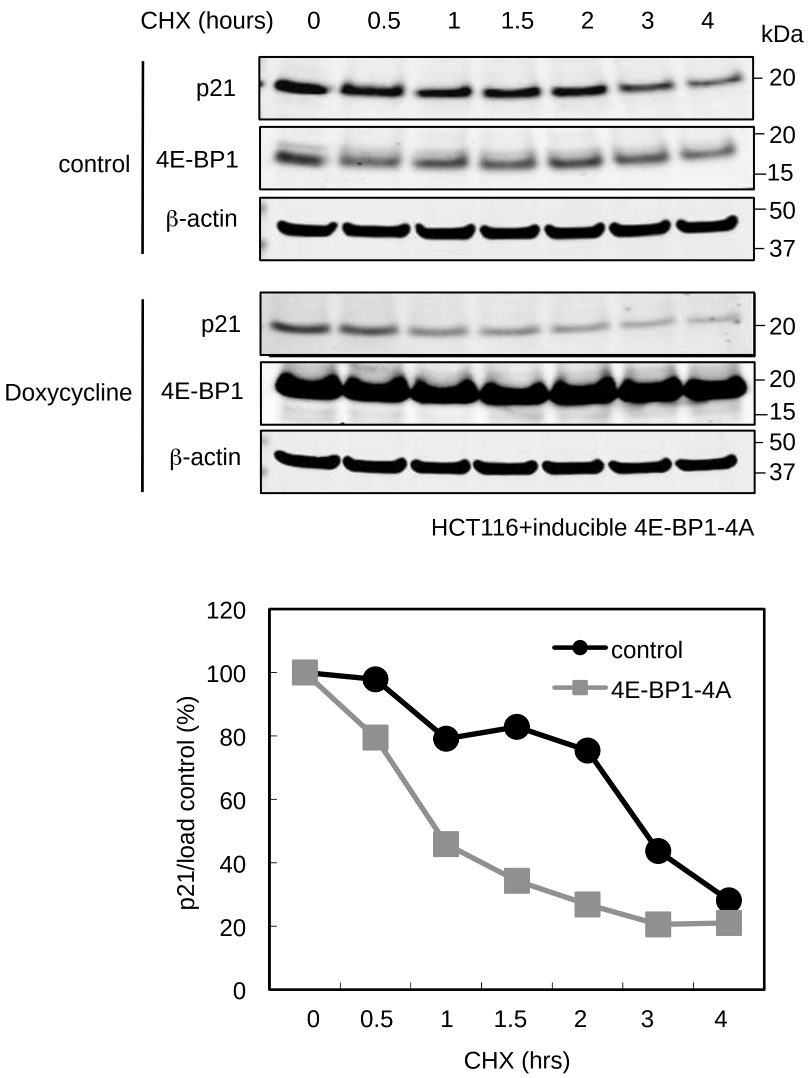
CHX (hours)
0
0.5
1
1.5
2
3
4
kDa
control
p21
4E-BP1
β-actin
Doxycycline
p21
4E-BP1
β-actin
20
20
15
50
37
20
20
15
50
37
HCT116+inducible 4E-BP1-4A
120
100
80
60
40
20
0
p21/load control (%)
control
4E-BP1-4A
0
0.5
1
1.5
2
3
4
CHX (hrs)

f

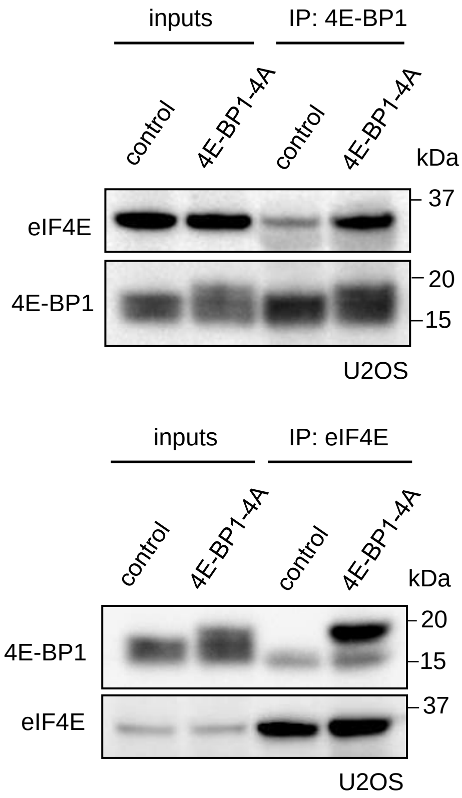
inputs
IP: 4E-BP1
control
4E-BP1-4A
control
4E-BP1-4A
kDa
eIF4E
4E-BP1
37
20
15
U2OS
inputs
IP: eIF4E
control
4E-BP1-4A
control
4E-BP1-4A
kDa
4E-BP1
eIF4E
20
15
37
U2OS

Supplementary Figure 3

(a) Western blot showing the phosphorylation status of protein kinases belonging to the PI3K/AKT/mTORC1 pathway in MEFs infected with either control or TSC2 shRNA-encoding lentiviruses.

(b) Western blot illustrating the effect of PI3K inhibitors on p21 protein levels in MEFs. Cells were treated for 24 hours with the indicated concentrations of the different drugs.

(c) p53KO MEFs were simultaneously infected with either scramble or TSC2 shRNAs and either control or 4E-BP1-4A-encoding lentiviruses. After selection, protein levels were analyzed by western blot.

(d) p21KO MEFs were infected with mouse p21, 4E-BP1-4A or their respectively control-encoding viruses. After selection protein levels were analyzed by western blot.

(e) Control and doxycycline-treated (24 hours) 4E-BP-4A-inducible HCT116 cells were incubated with 10 μM cyclohexamide (CHX) for the indicated times and, subsequently, p21 and 4E-BP1 levels were analyzed by Western blot (top). The densitometric quantification of p21 levels corrected by loading control is shown (bottom).

(f) U2OS cells were transfected with control or 4E-BP1-4A-expressing plasmid. After 48 hours, cell lysates were immunoprecipitated with either anti-4E-BP1 (top) or anti-eIF4E antibodies (bottom). Inputs and co-immunoprecipitated proteins were analyzed by Western blot.

For each panel, all the Western blots correspond to samples from the same experiment; in some cases, samples were distributed in several electrophoretic gels run in parallel.

Supplementary Fig. 4 (part 1 out of 2)

a

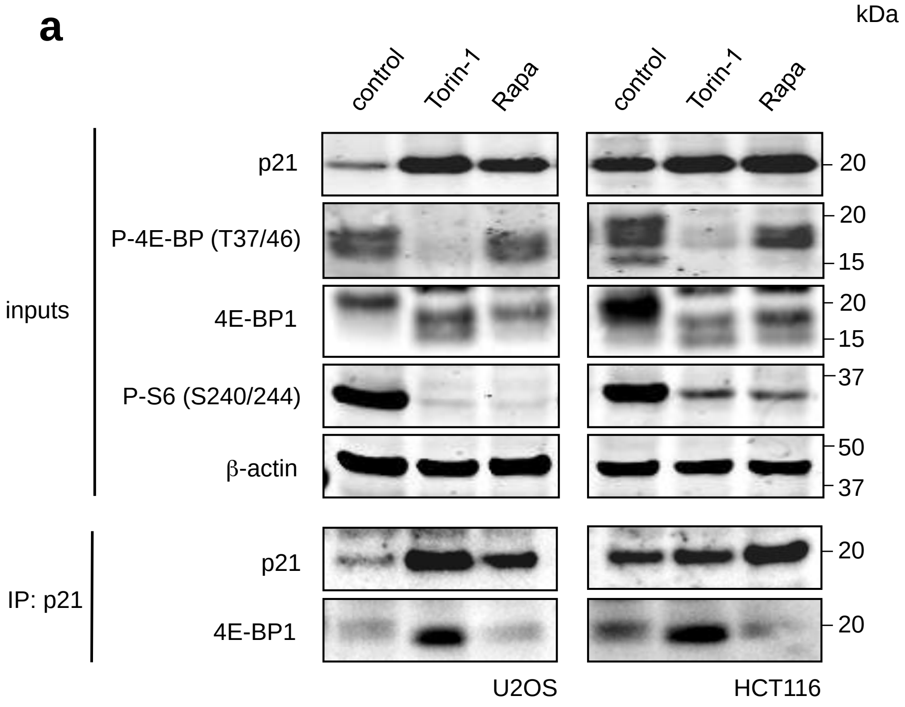

b

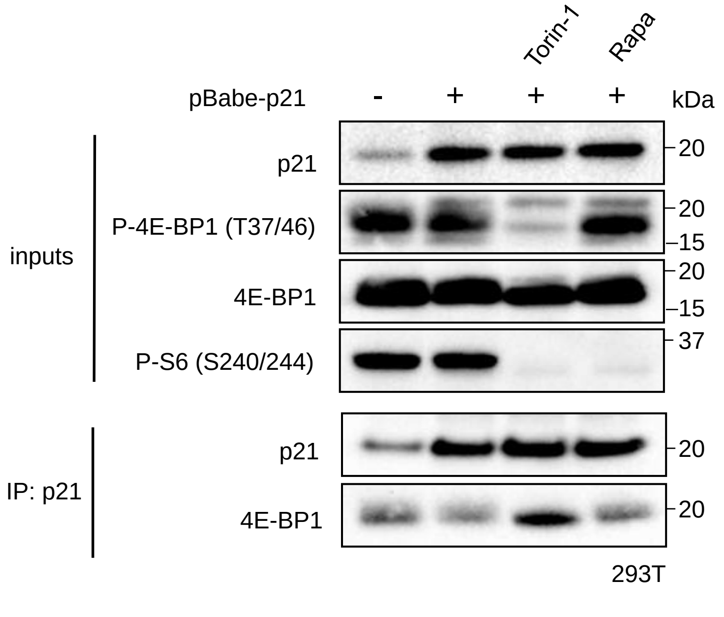

Supplementary Fig. 4 (part 2 out of 2)

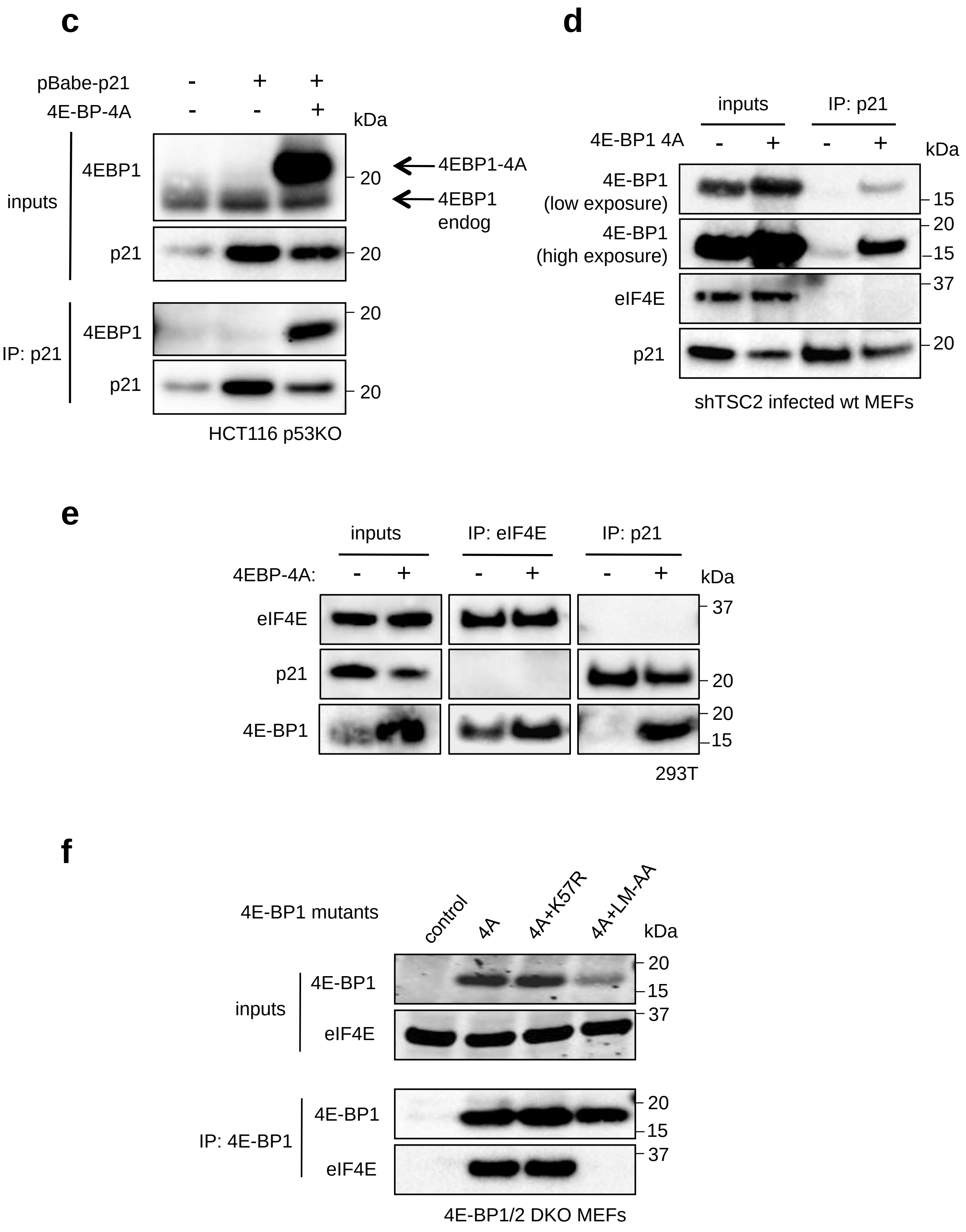

Supplementary Figure 4

(a) U2OS and HCT116 cells were treated with Torin-1 (250 nM) or Rapamycin (Rapa; 100 nM) for 5 hours in the presence of the proteasomal inhibitor MG132. Cell lysates were immunoprecipitated with anti-p21 antibody and resolved by Western blot.

(b) 293T cells were transfected with either control or pBabe-p21 (human p21) plasmids and, 24 hours later, incubated with either torin-1 or rapamycin (Rapa) for 5 hours in the presence of the proteasomal inhibitor MG132. Following p21 immunoprecipitation, inputs and interacting proteins were analyzed by Western blot.

(c) p53KO HCT116 cells were co-transfected with human p21 and 4E-BP1-4A-expressing plasmids. Following 5 hours incubation with the proteasomal inhibitor MG132, p21 was immunoprecipitated and, subsequently, inputs and interacting proteins were analyzed by Western blot.

(d) MEFs were infected with TSC2 shRNAs together with either control or 4E-BP1-4A-encoding lentiviruses. Cells were incubated with the proteasomal inhibitor MG132 for 5 hours prior protein extraction.

(e) 293T cells were transfected with control or 4E-BP1-4A-encoding plasmids. Cell lysates were immunoprecipitated with either anti-eIF4E or anti-p21 antibodies and interacting proteins were resolved by Western blot.

(f) 4E-BP1/1 DKO MEFs were infected with the indicated 4E-BP1 mutant-encoding lentiviruses. Cell lysates were immunoprecipitated with anti-4E-BP1 antibodies and analyzed by Western blot.

For each panel, all the Western blots correspond to samples from the same experiment; in some cases, samples were distributed in several electrophoretic gels run in parallel.

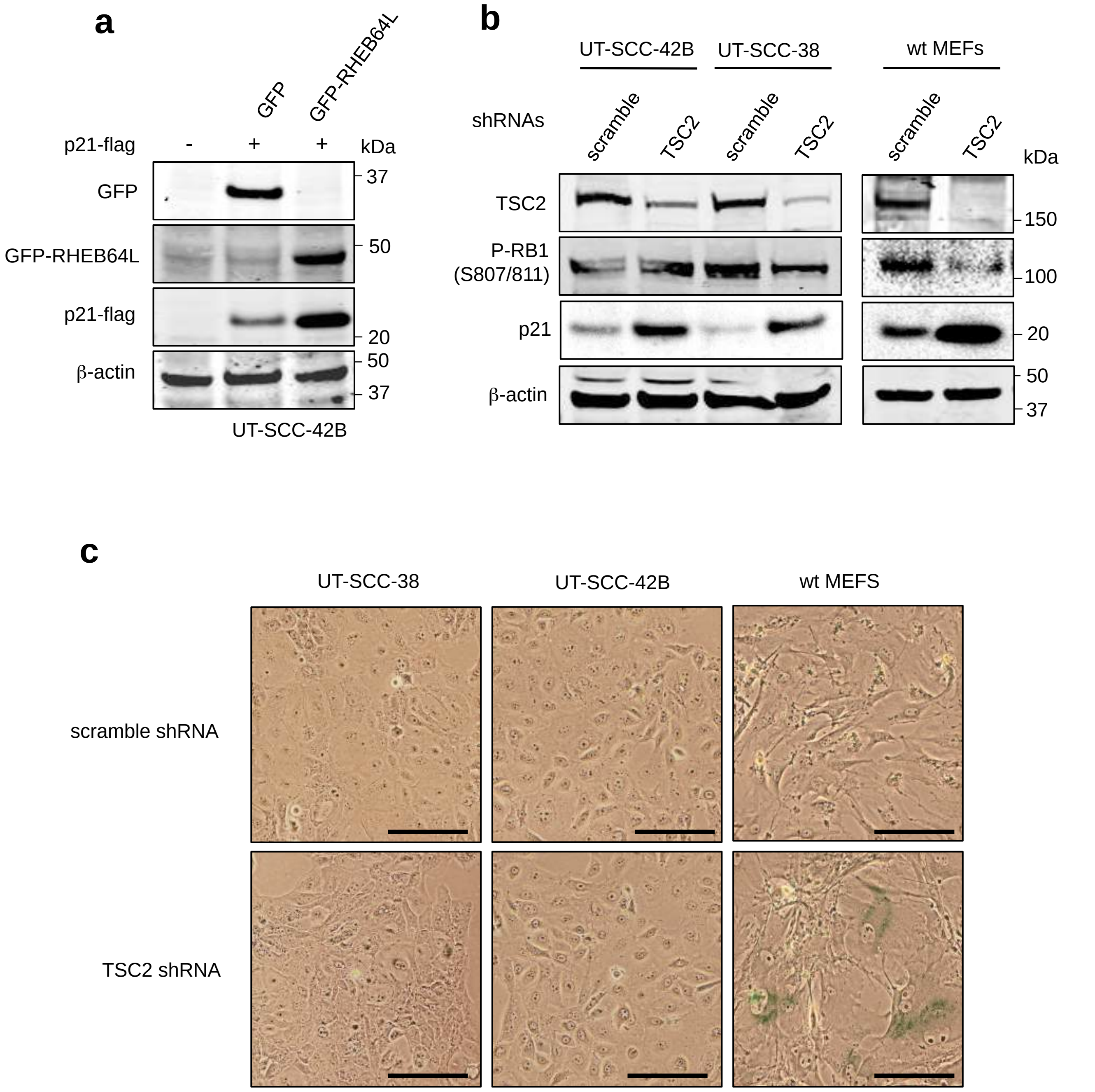
a
GFP
GFP-RHEB64L
p21-flag
-
+
+
kDa
GFP
37
GFP-RHEB64L
50
p21-flag
20
β-actin
50
37
UT-SCC-42B
b
UT-SCC-42B
UT-SCC-38
wt MEFs
shRNAs
scramble
TSC2
scramble
TSC2
scramble
TSC2
kDa
TSC2
150
P-RB1
(S807/811)
100
p21
20
β-actin
50
37
c
UT-SCC-38
UT-SCC-42B
wt MEFS
scramble shRNA
TSC2 shRNA

Supplementary Fig. 5 (part 2 out of 2)

d

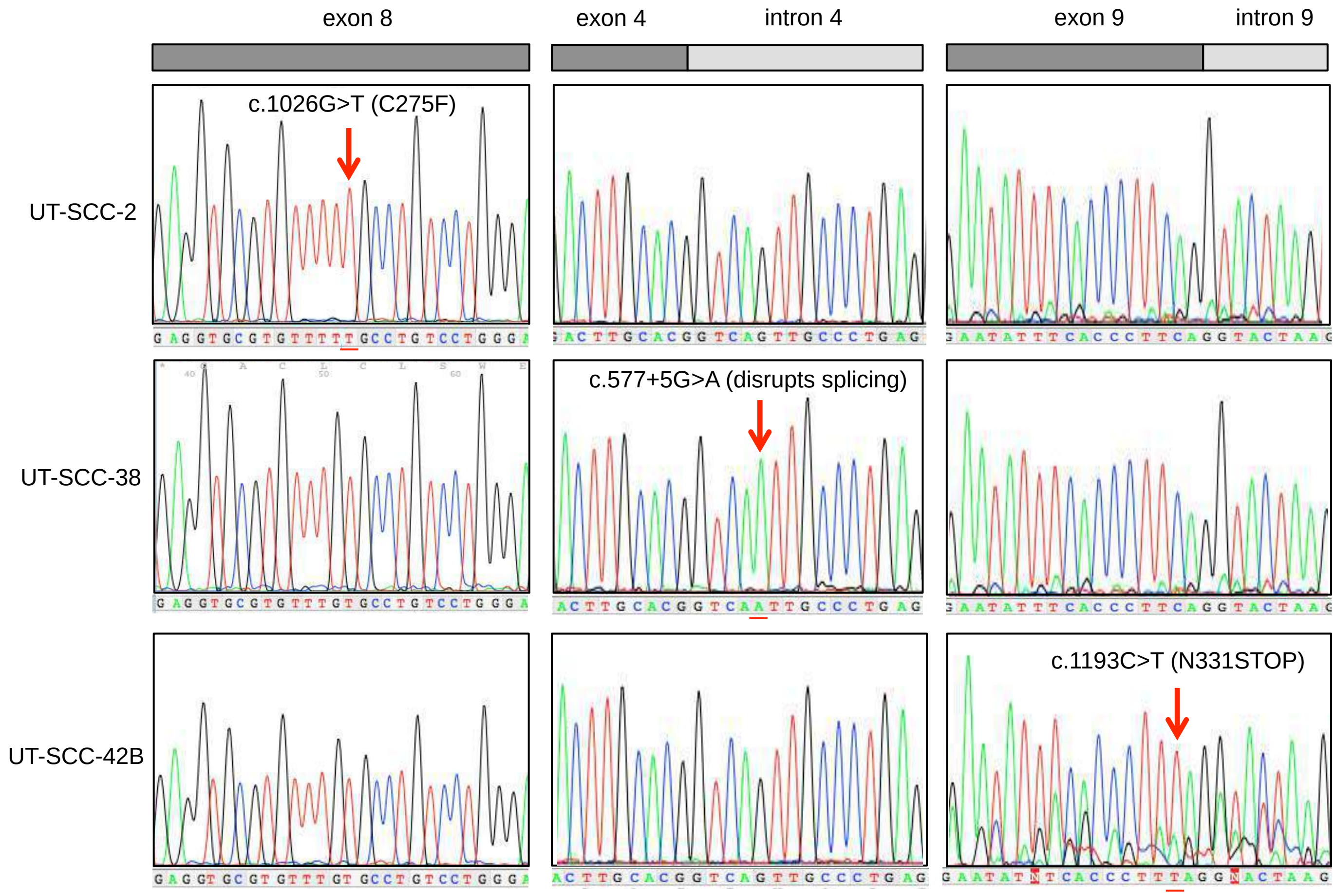

e

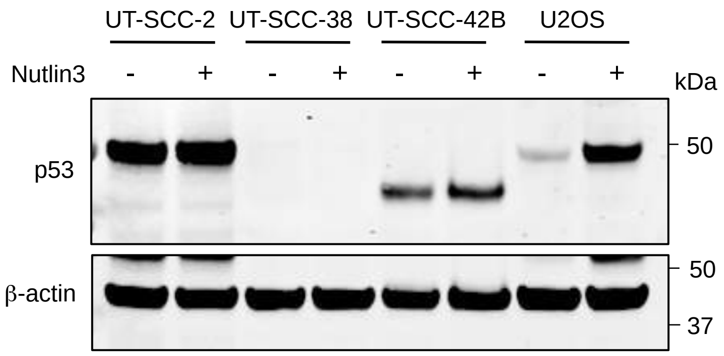

Supplementary Figure 5

(a) UT-SCC-42B cells were co-transfected with human p21-flag and either control, GFP or GFP-Rheb64L-encoding plasmids. Protein expression was analyzed by Western blot.

(b) HNSCC cell lines (UT-SCC-38 and UT-SCC-42B) and wt MEFs were infected with either scramble or TSC2 shRNA-encoding lentiviruses. The phosphorylation levels of RB1 protein was analyzed by Western blot.

(c) Representative images illustrating β-galactosidase activity in HNSCC cell lines infected with either scramble or TSC2 shRNA-encoding lentiviruses. Scale bar 100 μm.

(d) Graphic depiction of the mutations found in the genomic sequences of p53 in the HNSCC cell lines. UT-SCC-2 has point mutation in codon 275 which results in the change Cys>Phe. This is a known deleterious mutation (see IARC TP53 Database at http://p53.iarc.fr). UT-SCC-38 has a point mutation in the conserved splice donor site (intron position +5) of intron 4. Mutations in this sequence are known to result in a variety of alternatively spliced mRNAs encoding non-functional truncated proteins. UT-SCC-42B has a point mutation in codon 331, which results in a stop codon.

(e) Western blot illustrating the effect of the Mdm2 inhibitor Nutlin3 on the levels of p21 and p53 proteins in different HNSCC cell lines. The human osteosarcoma cell line U2OS (wt p53) was included as a positive control.

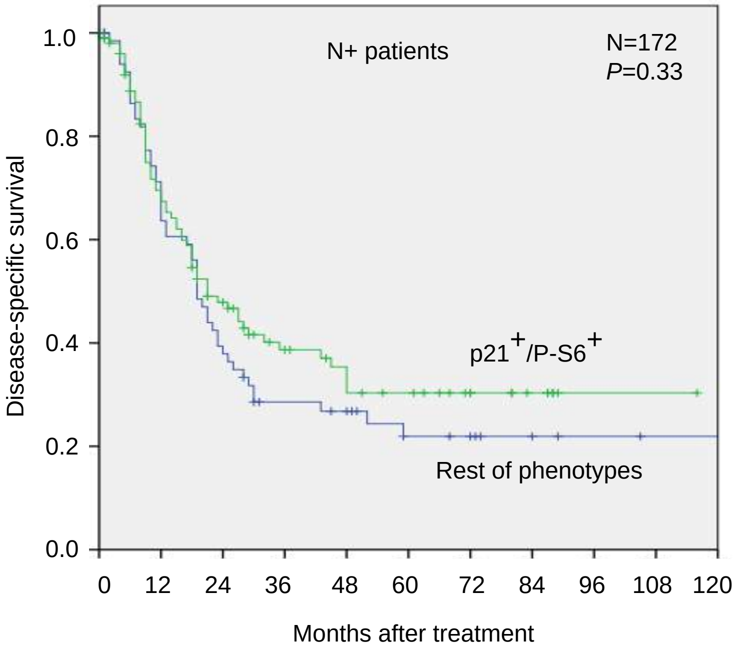

Supplementary Figure 6

Kaplan-Meier disease-specific survival curves of patients with regional lymph node involvement at the time of diagnosis (N+) categorized by p21 and P-S6 (240/244) (n=172; *P*=0.33; log-rank test).

Supplementary Table 1. Cohort characteristics

Characteristics	Case no. (n=274)	%
**Tumor localization**		
Oropharynx	141	51
Larynx	65	24
Hypopharynx	68	25
**Age**		
>60	123	45
<60	151	55
**Sex**		
Male	262	96
Female	12	4
**Tobacco smokers**	264	96
Moderate	164	60
Heavy	100	37
**Alcohol consumption**	241	88
**TNM stage**		
I	18	7
II	14	5
III	42	15
IV	200	73
**Differentiation**		
W/D	100	37
M/D	110	40
P/D	64	23
**pT classification**		
T1	32	12
T2	48	17
T3	90	33
T4	104	38
**LN metastasis**		
Negative (N0)	56	20
Positive (N1-3)	172	63
**HPV infection**		
Negative	274	100
Positive	0	0

Supplementary Table 2. p53 and p21 expression in HNSCC

	**p53 positive**	**p53 negative**	***P***[†]
**p21 positive**	100	55	0.294
**p21 negative**	43	32	

[†]Fisher Exact test (two sided), P=0.312

Supplementary Table 3. P-S6 and p21 expression in HNSCC

	**P-S6 positive**	**P-S6 negative**	***P***[†]
**p21 positive**	143	11	<0.001
**p21 negative**	21	53	

[†]Fisher Exact test (two sided), P<0.001

Supplementary Table 4. Cox regression table

**Parameter**	**HR (95% CI)**	***P***
**Localization (larynx)**	0.86 (0.54-1-37)	0.52
**T classification (T3-T4)**	1.2 (0.79-1.81)	0.37
**N classification (N+)**	2.78 (1.7-4.56)	<0.001
**Pathological grade (G3)**	1.1 (0.87-1.4)	0.41
**p21/P-S6 phenotype (+/+)**	0.74 (0.52-1.05)	0.95

Supplementary Table 5. p21 and Ki67 expression in HNSCC

Ki67	0-25%	25-50%	51-75%	>75%	total
p21 positive	14	17	26	8	65
p21 negative	6	9	10	4	29
total	20	26	36	12	94

†Fisher Exact test (two sided), P=0.946

Supplementary Table 6. p21$^+$/P-S6$^+$ expression and tumour recurrence in HNSCC

	p21+/P-S6+	Rest of patients	*P*†
Recurrence	80	62	0.011
No-recurrence	63	23	

†Fisher Exact test (two sided), P=0.011

Supplementary Table 7. p21$^+$/P-S6$^+$ expression and distant metastasis

	p21+/P-S6+	Rest of patients	*P*†
Distant metastasis	46	43	<0.01
Absence of distant metastasis	97	42	

†Fisher Exact test (two sided), P=0.008

Supplementary Table 8. p21$^+$/P-S6$^+$ expression and tumor recurrence in N0 patients

	p21+/P-S6+	Rest of patients	$P^†$
Recurrence	16	10	0.15
No-recurrence	24	6	

^{T}Fisher Exact test (two sided), P=0.149

Supplementary Table 9. p21$^+$/P-S6$^+$ expression and distant metastasis in N0 patients

	p21+/P-S6+	Rest of patients	$P^†$
Distant metastasis	8	6	0.19
Absence of distant metastasis	32	10	

^{T}Fisher Exact test (two sided), P=0.189

Supplementary Note 1

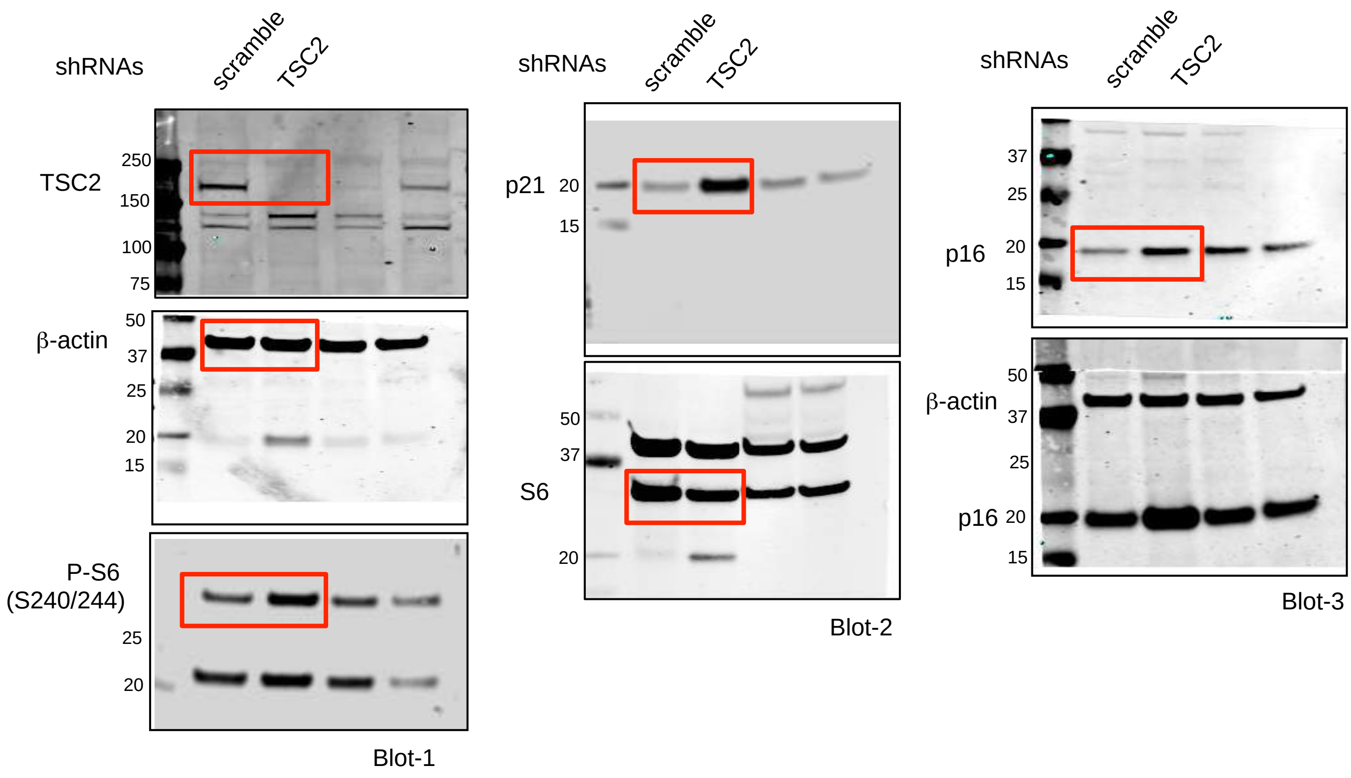

Uncropped membranes for Figure 1a

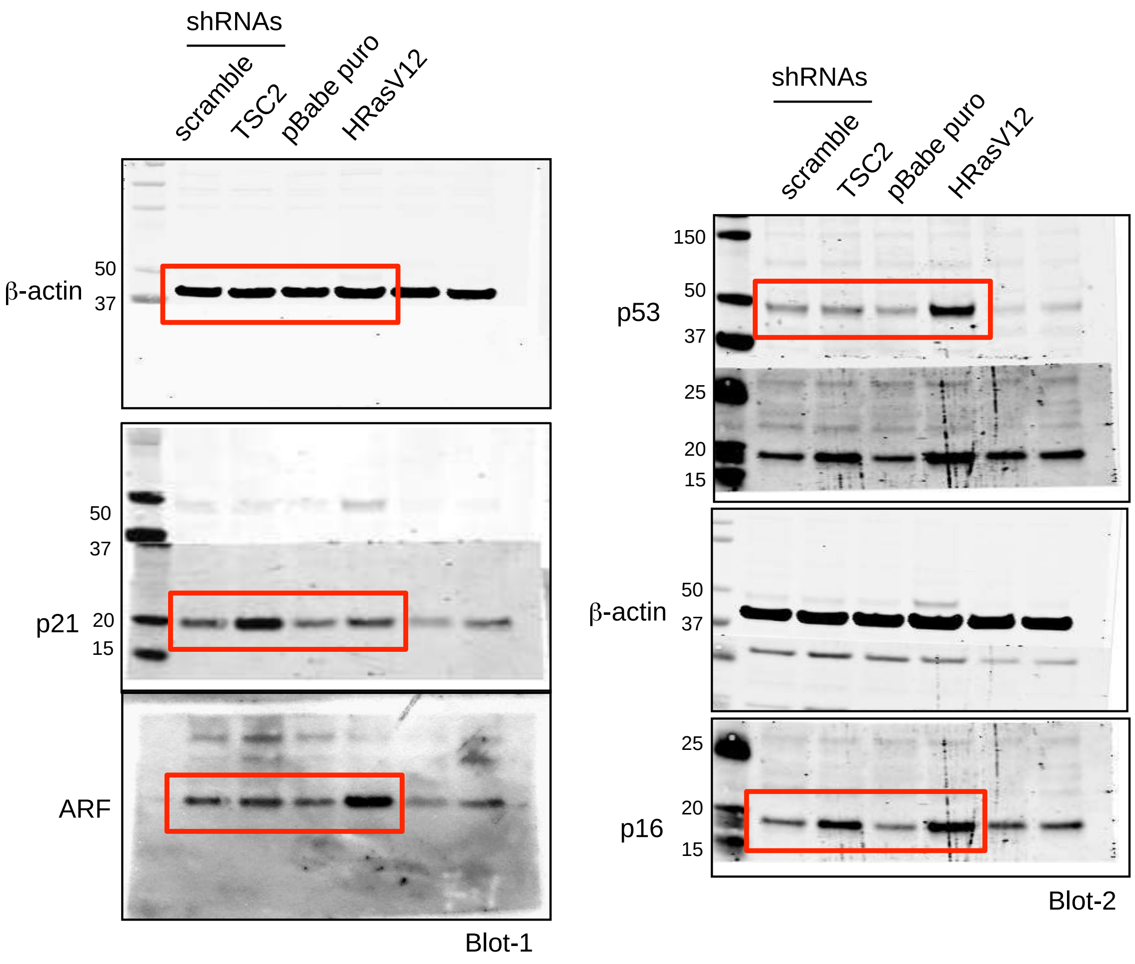

Uncropped membranes for Figure 1b

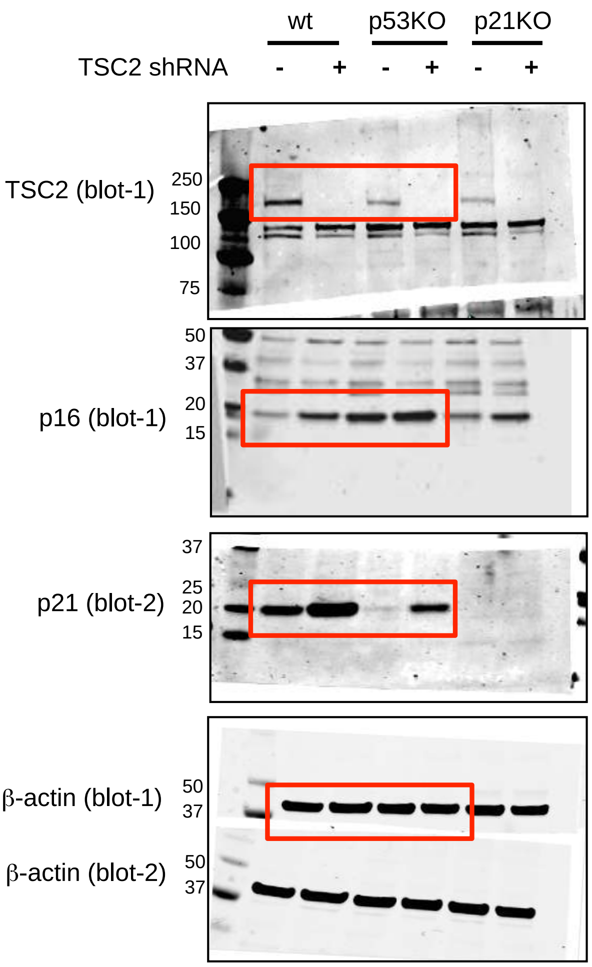

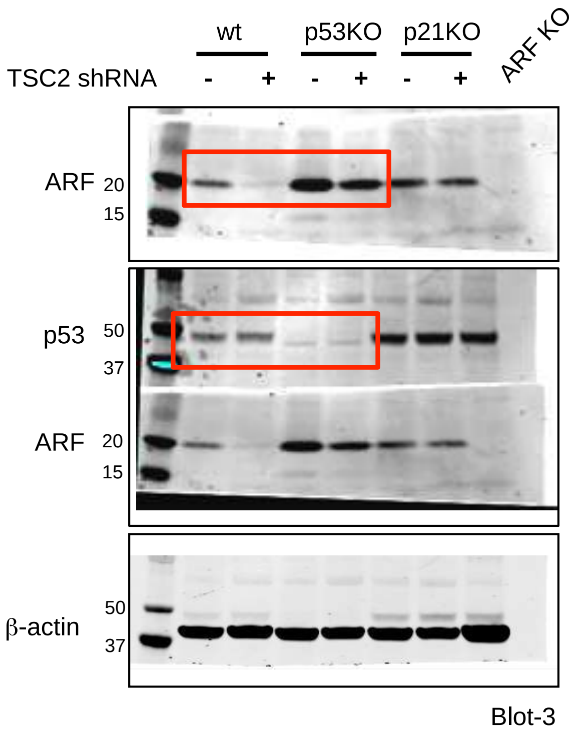

Uncropped membranes for Figure 1c

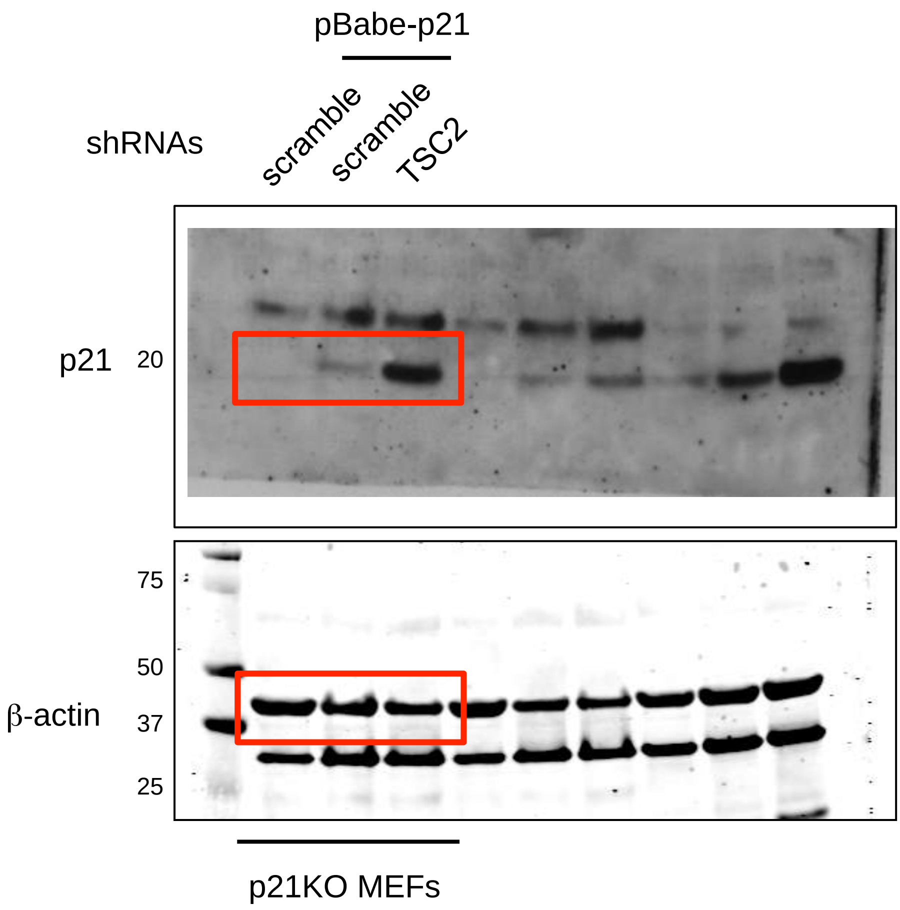

Uncropped membranes for Figure 2a

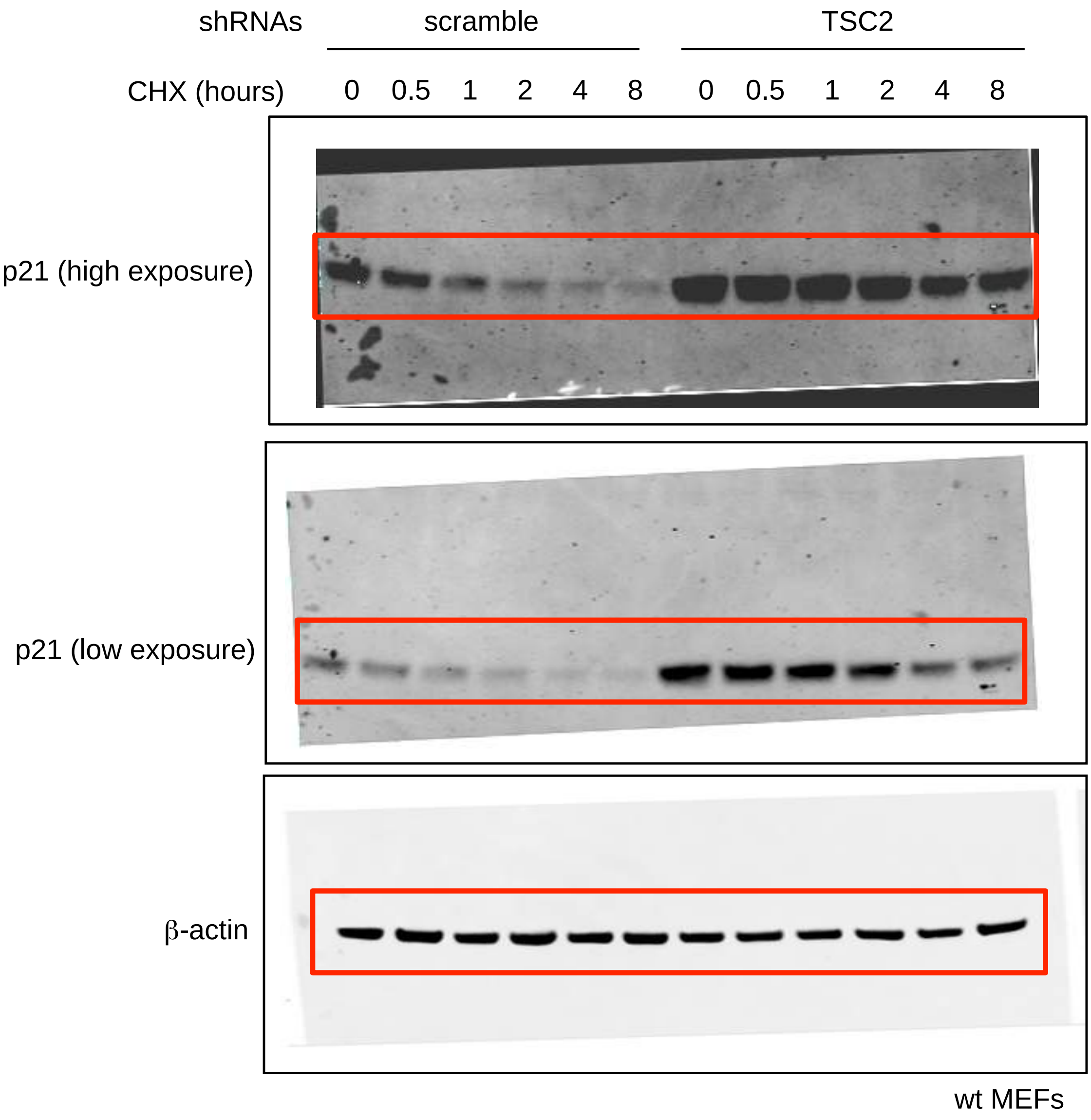

Uncropped membranes for Figure 1b

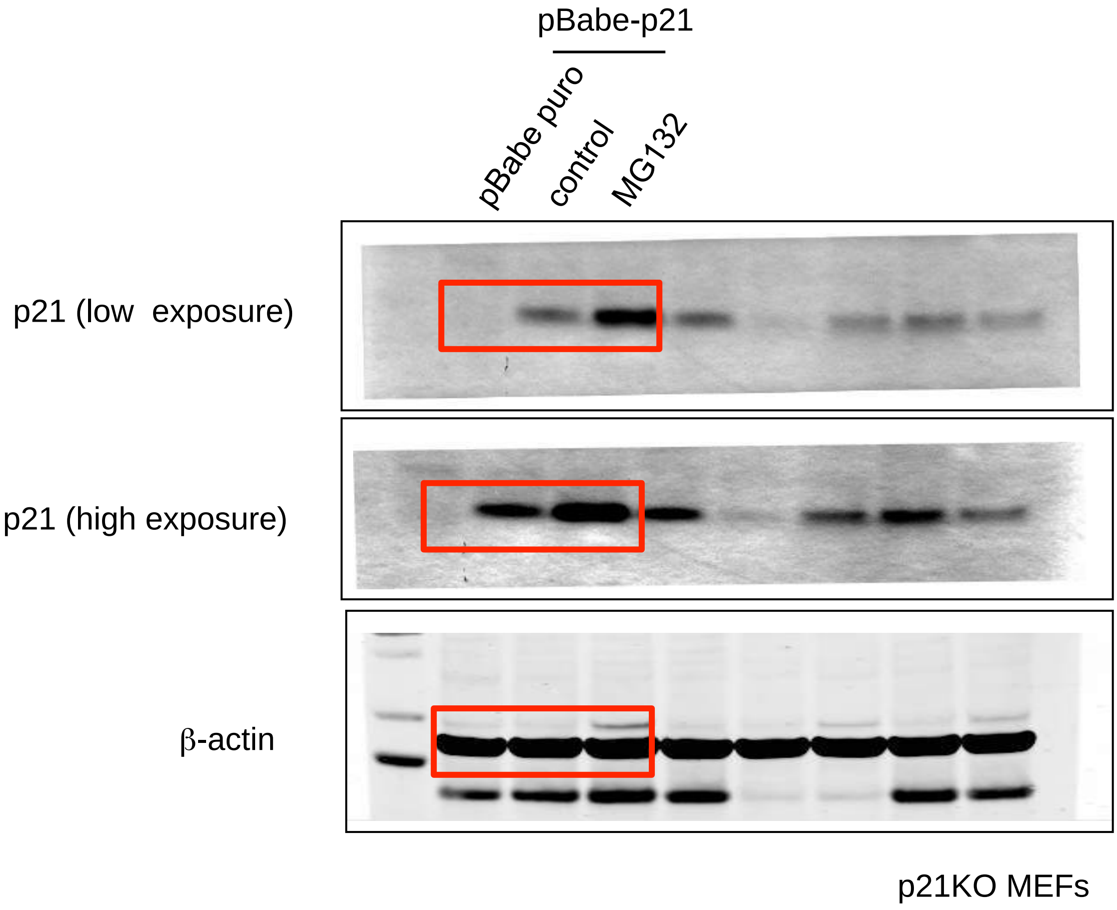

Uncropped membranes for Figure 2c

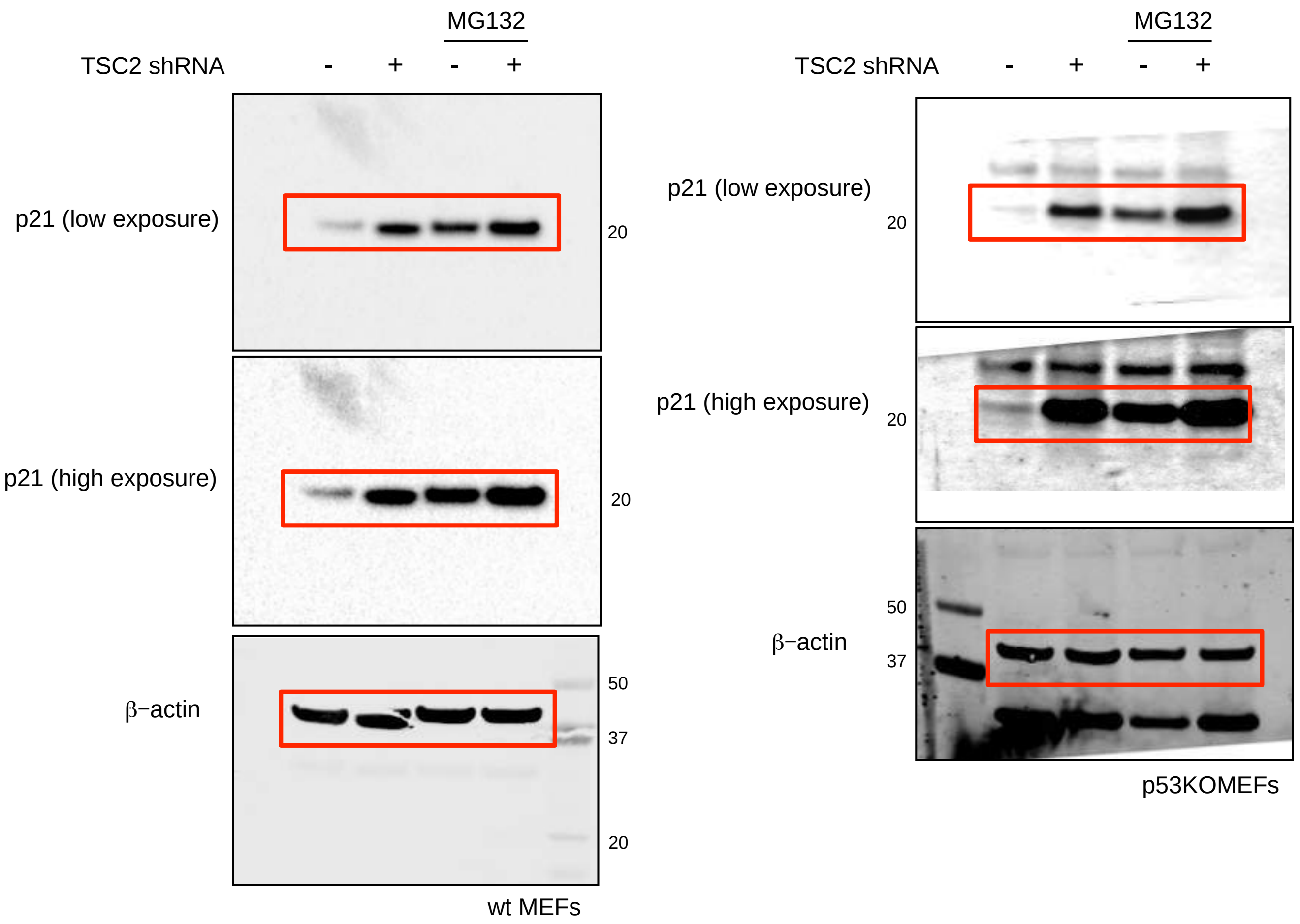

Uncropped membranes for Figure 2d

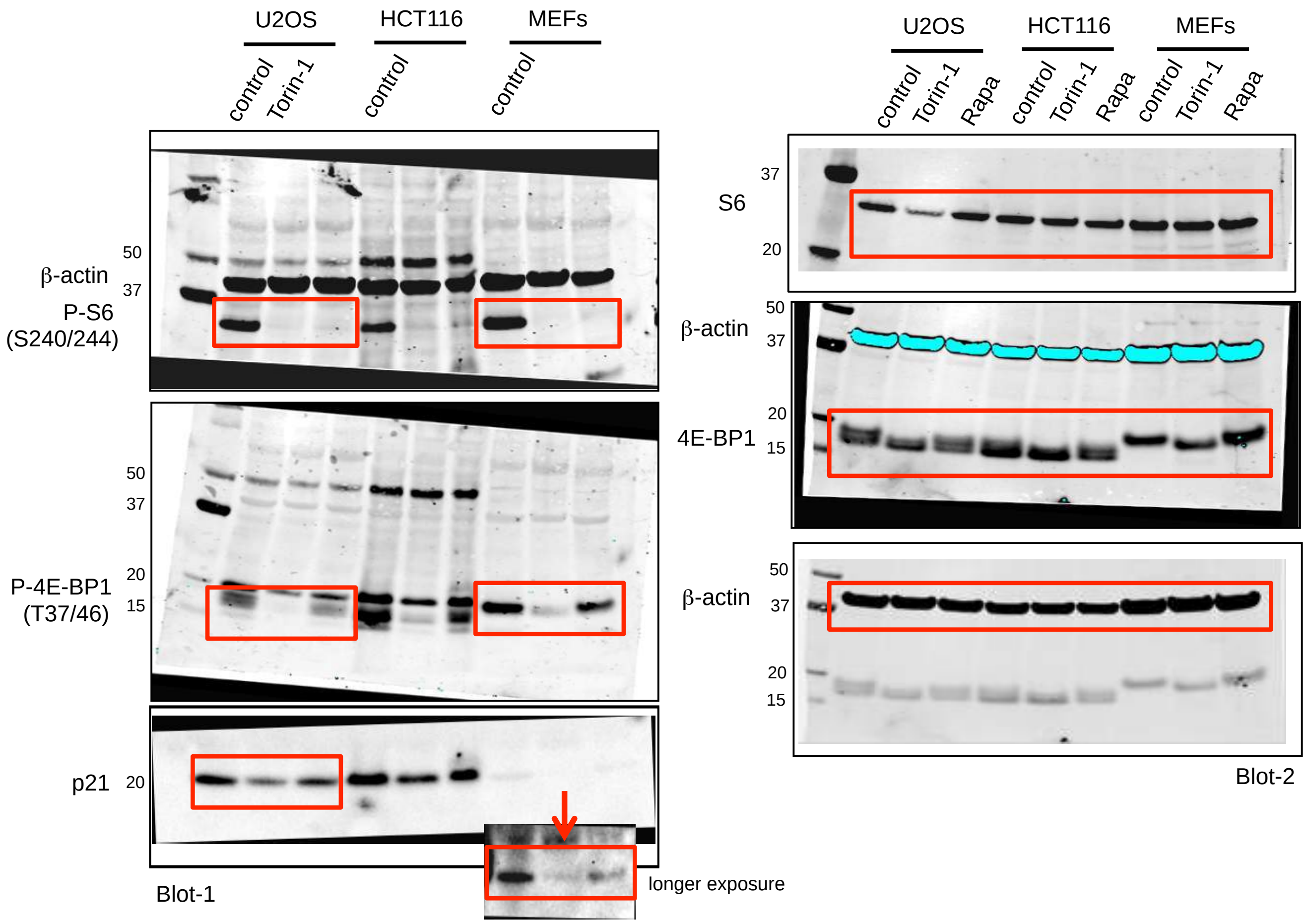

Uncropped membranes for Figure 3a (1)

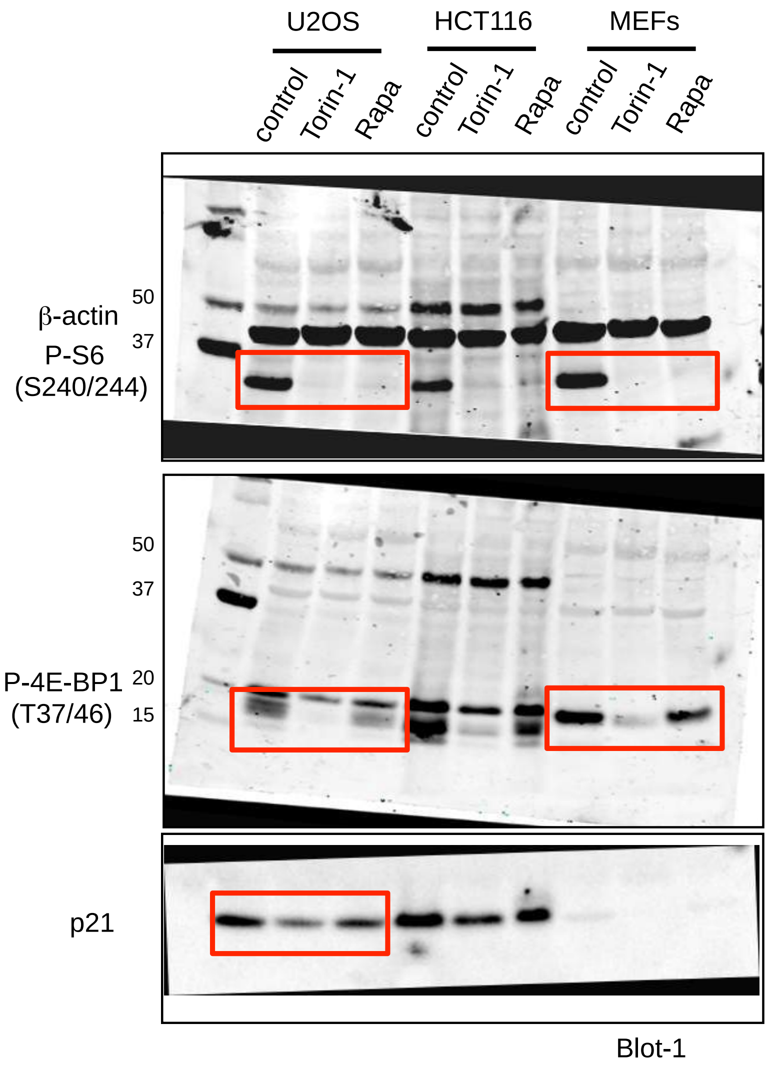

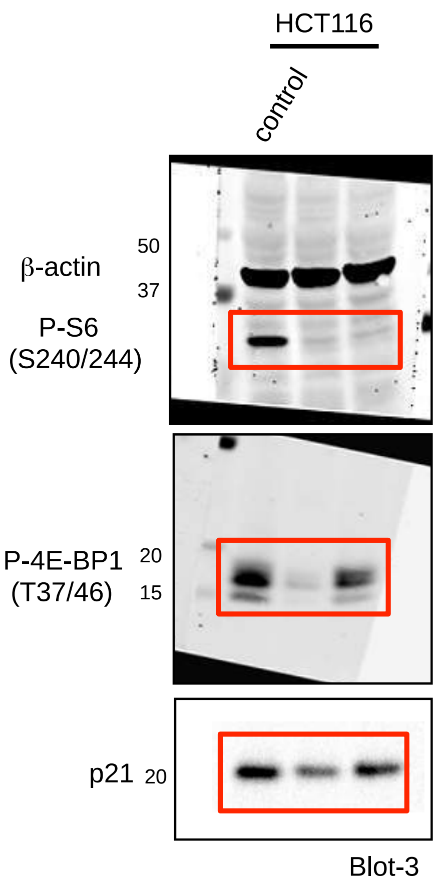

Uncropped membranes for Figure 3a (2)

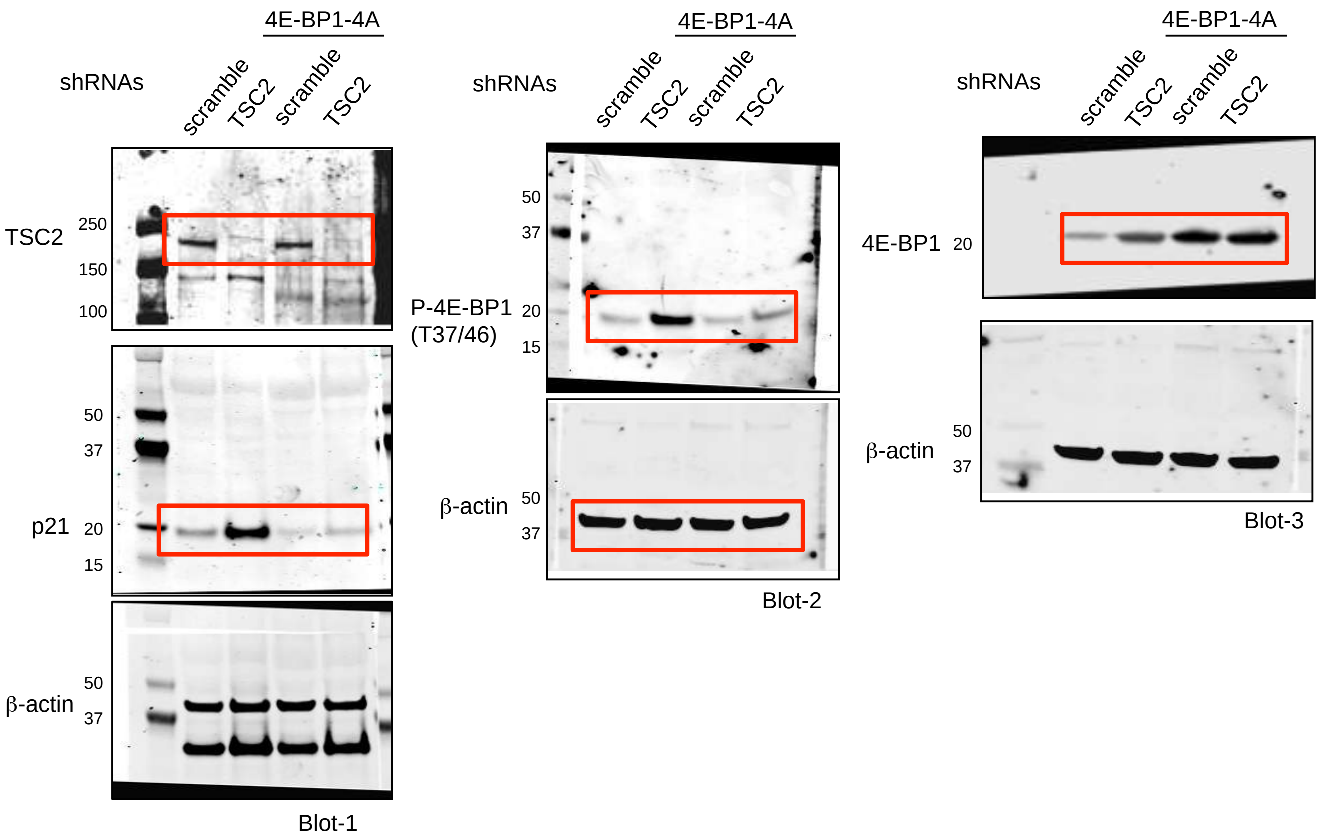

Uncropped membranes for Figure 3b

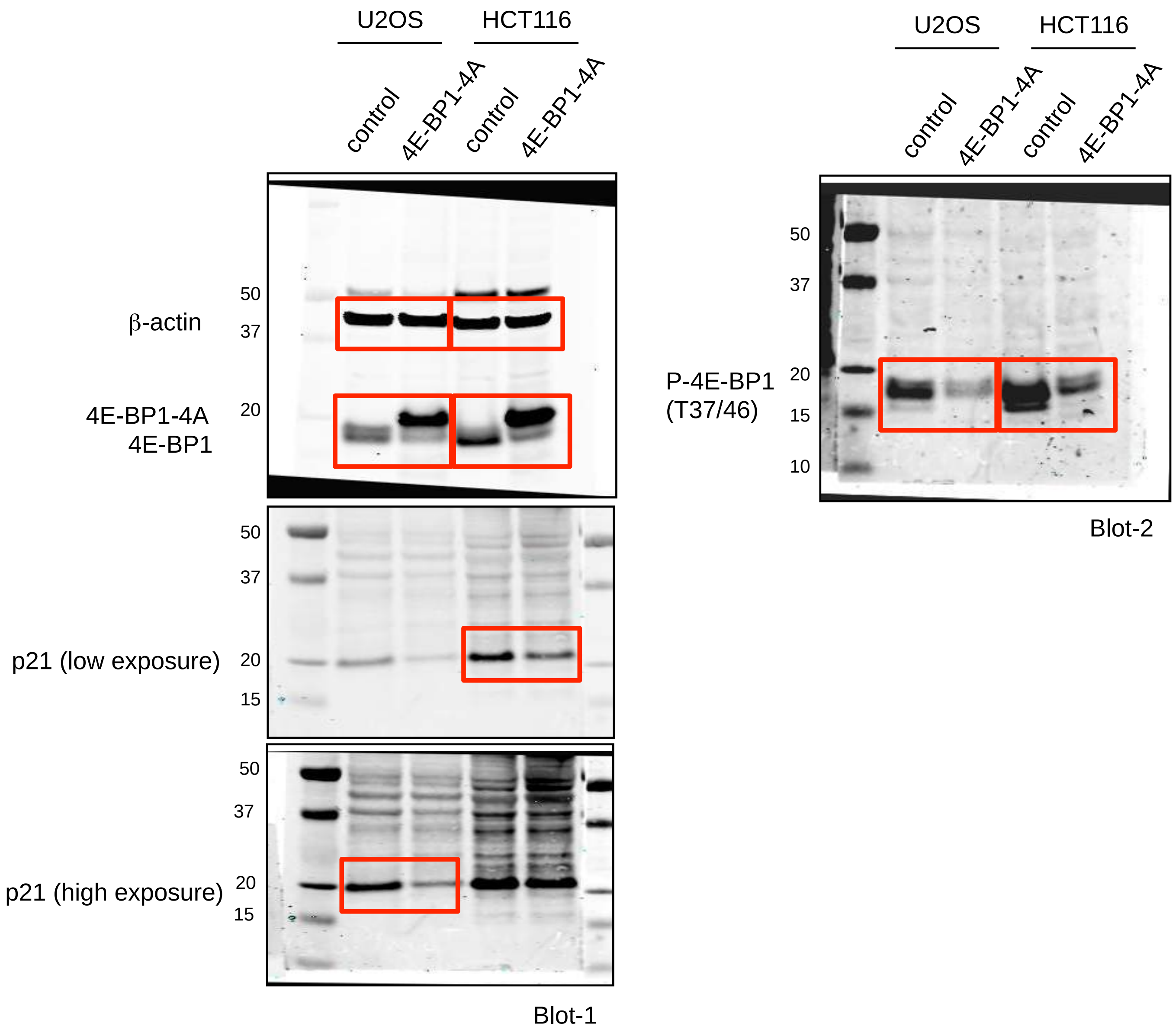

Uncropped membranes for Figure 3c (1)

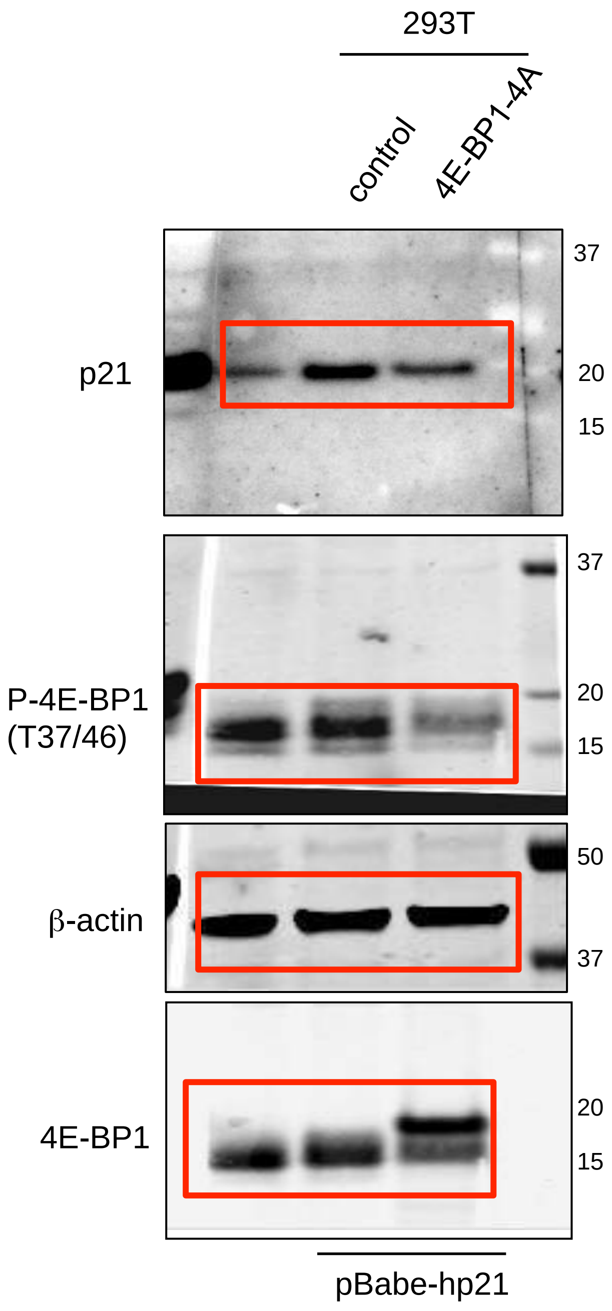

Uncropped membranes for Figure 3c (2)

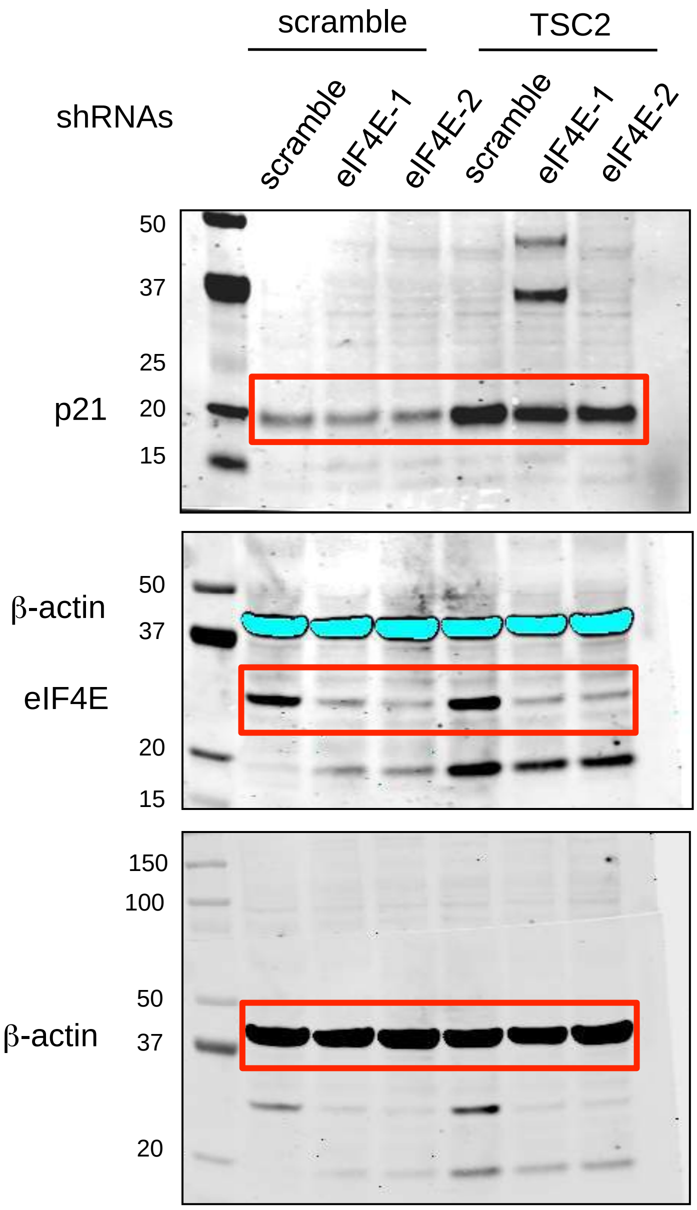

Uncropped membranes for Figure 3d

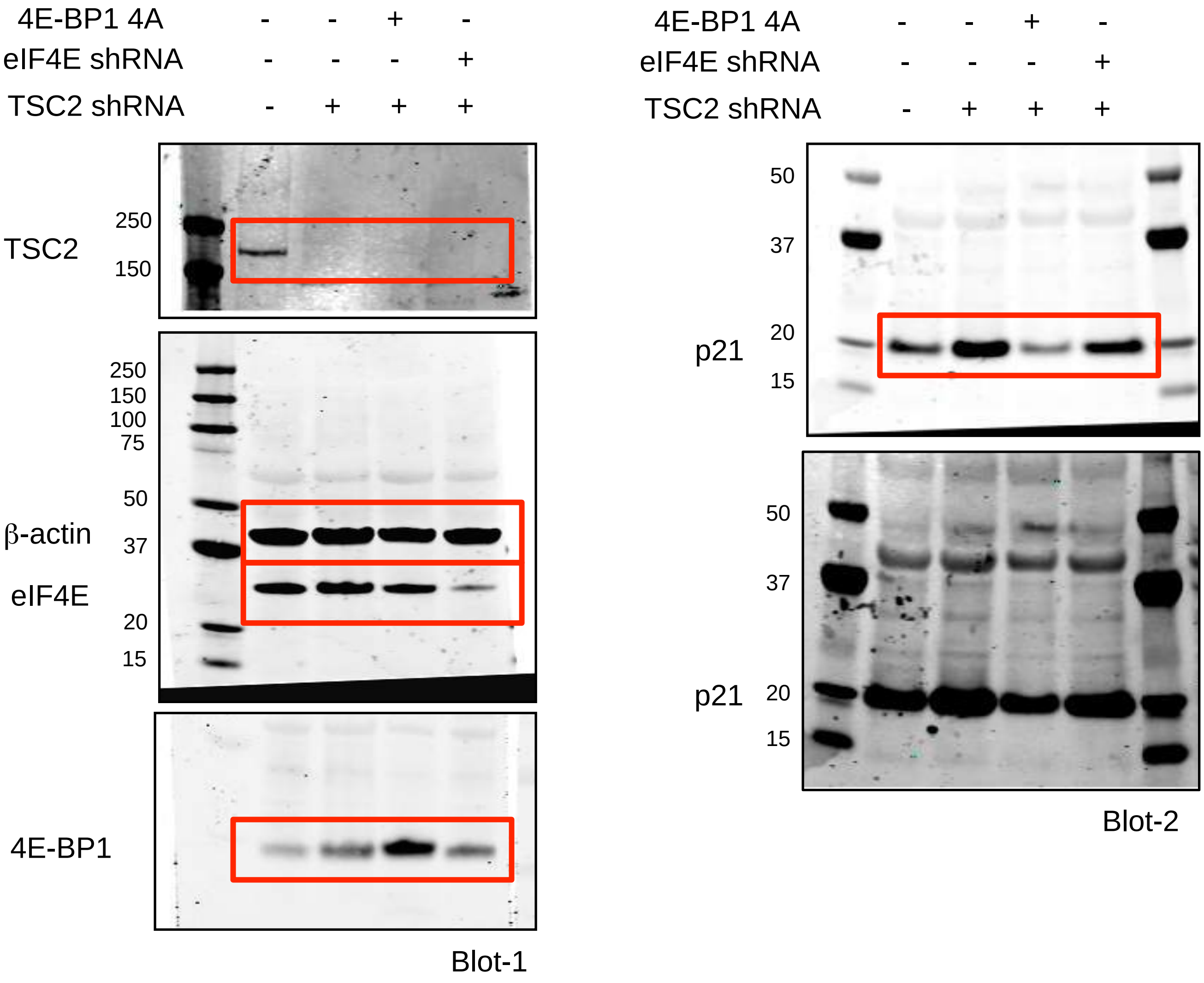

Uncropped membranes for Figure 3e

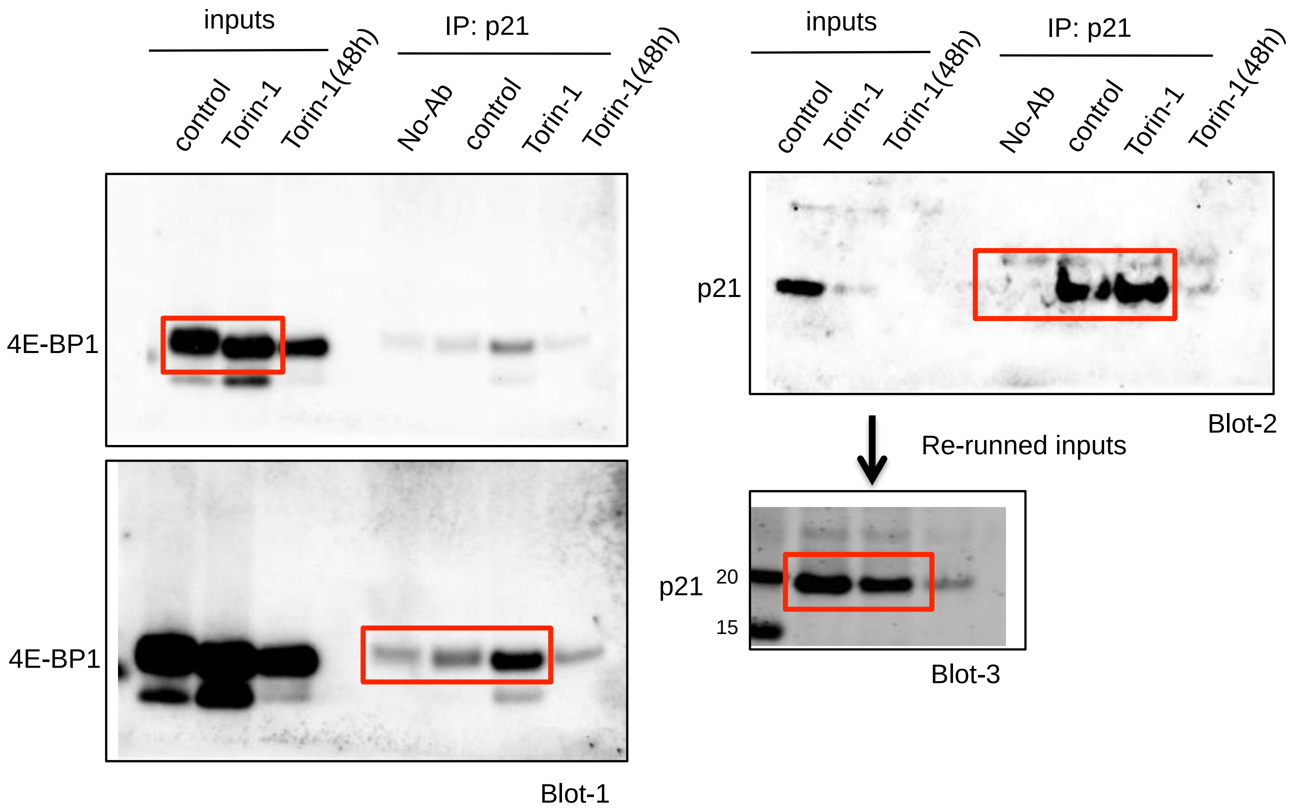

Uncropped membranes for Figure 4a

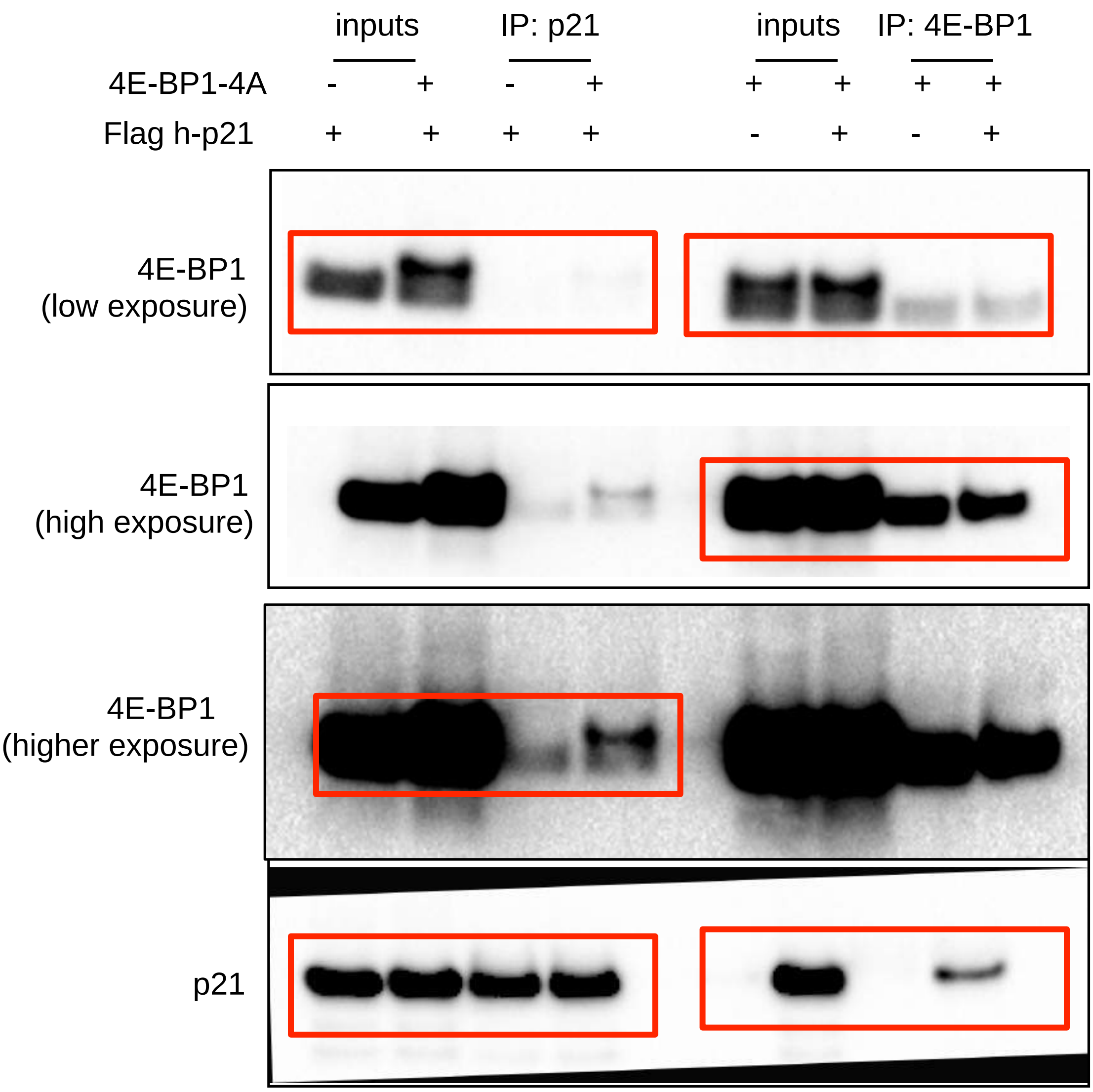

Uncropped membranes for Figure 4b

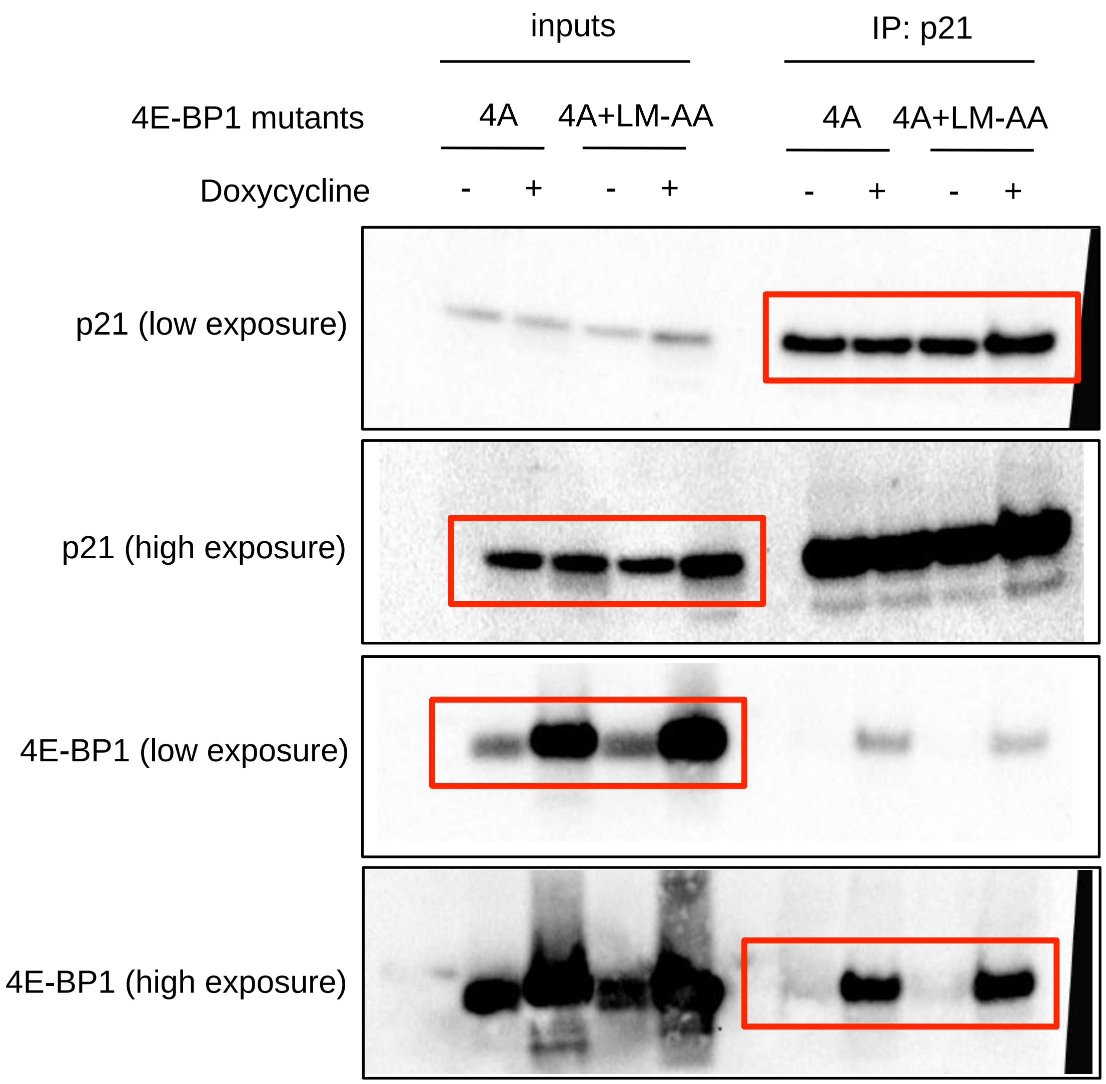

Uncropp membranes for Figure 4c

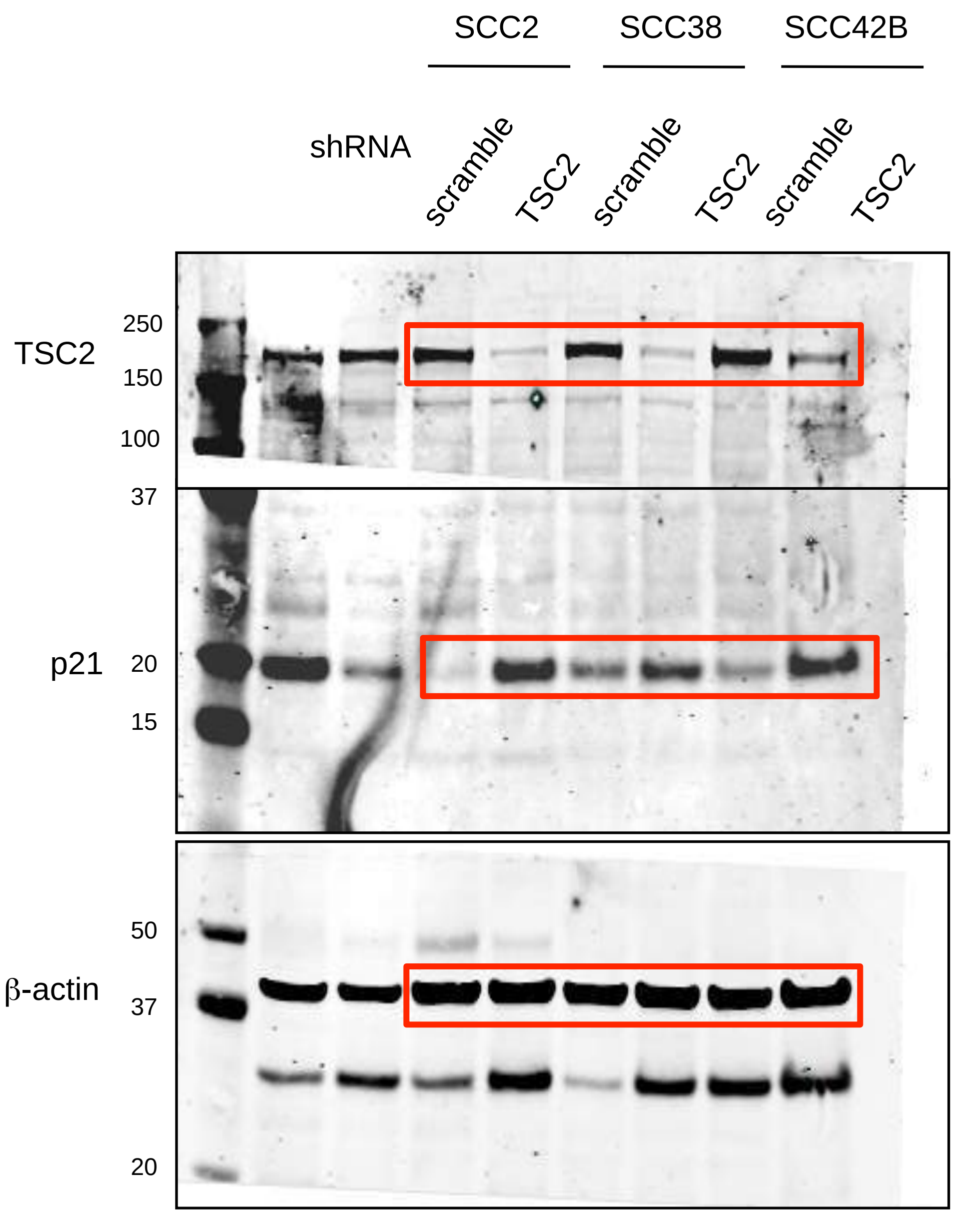

Uncropped membranes for Figure 5c

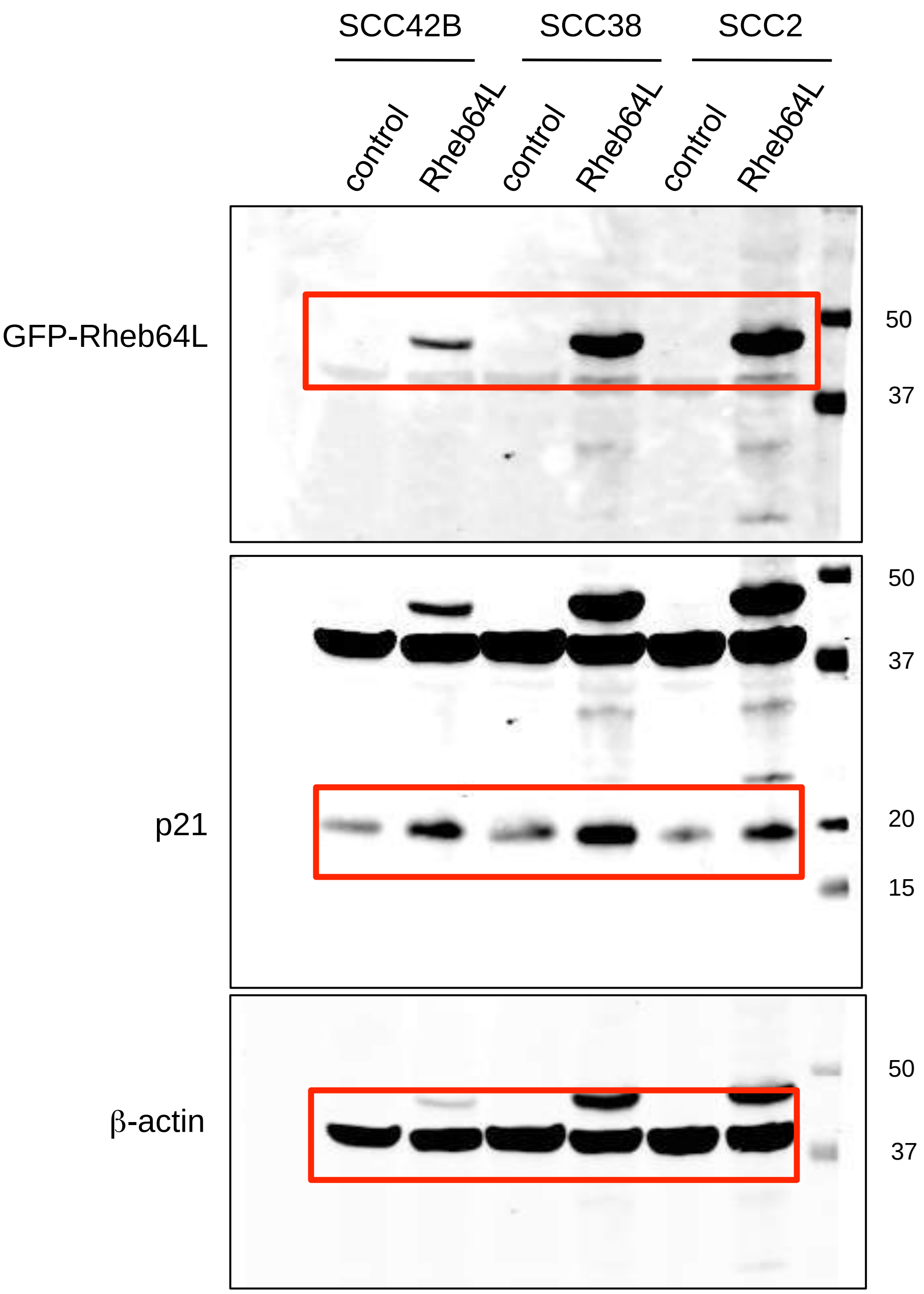

Uncropped membranes for Figure 5d

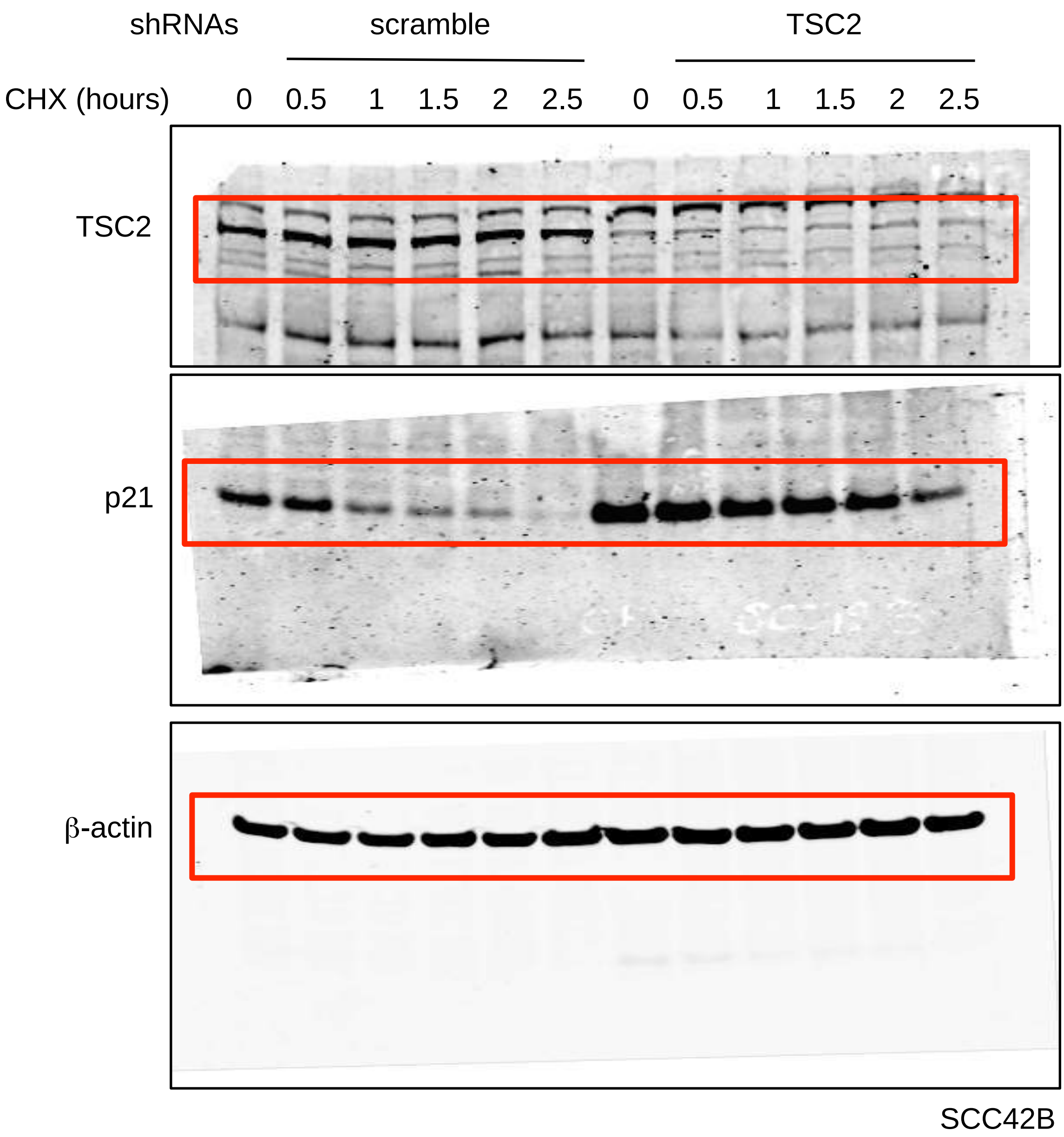

Uncropped membranes for Figure 5e

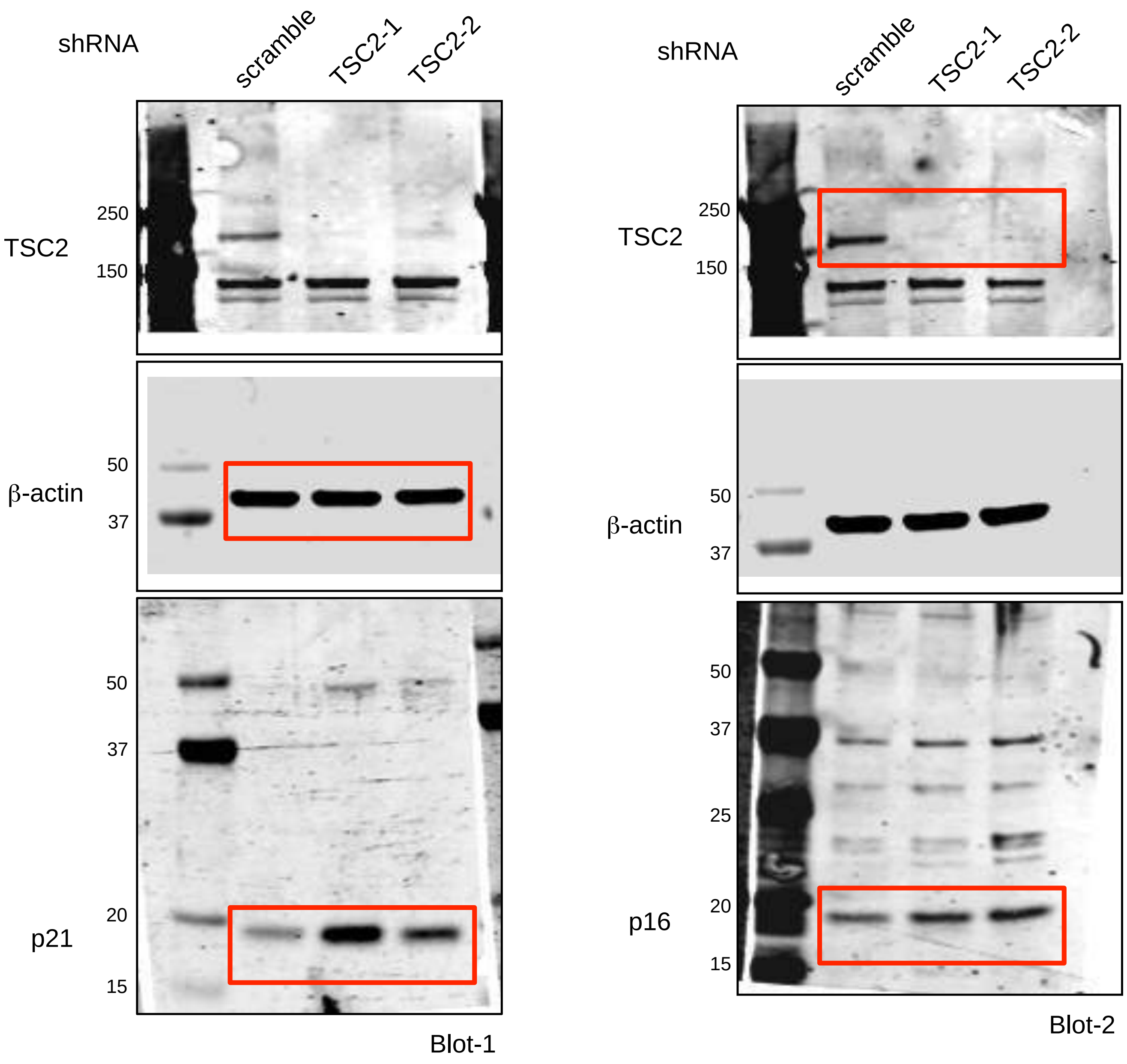

Uncropped membranes for Supplementary Fig. 1a

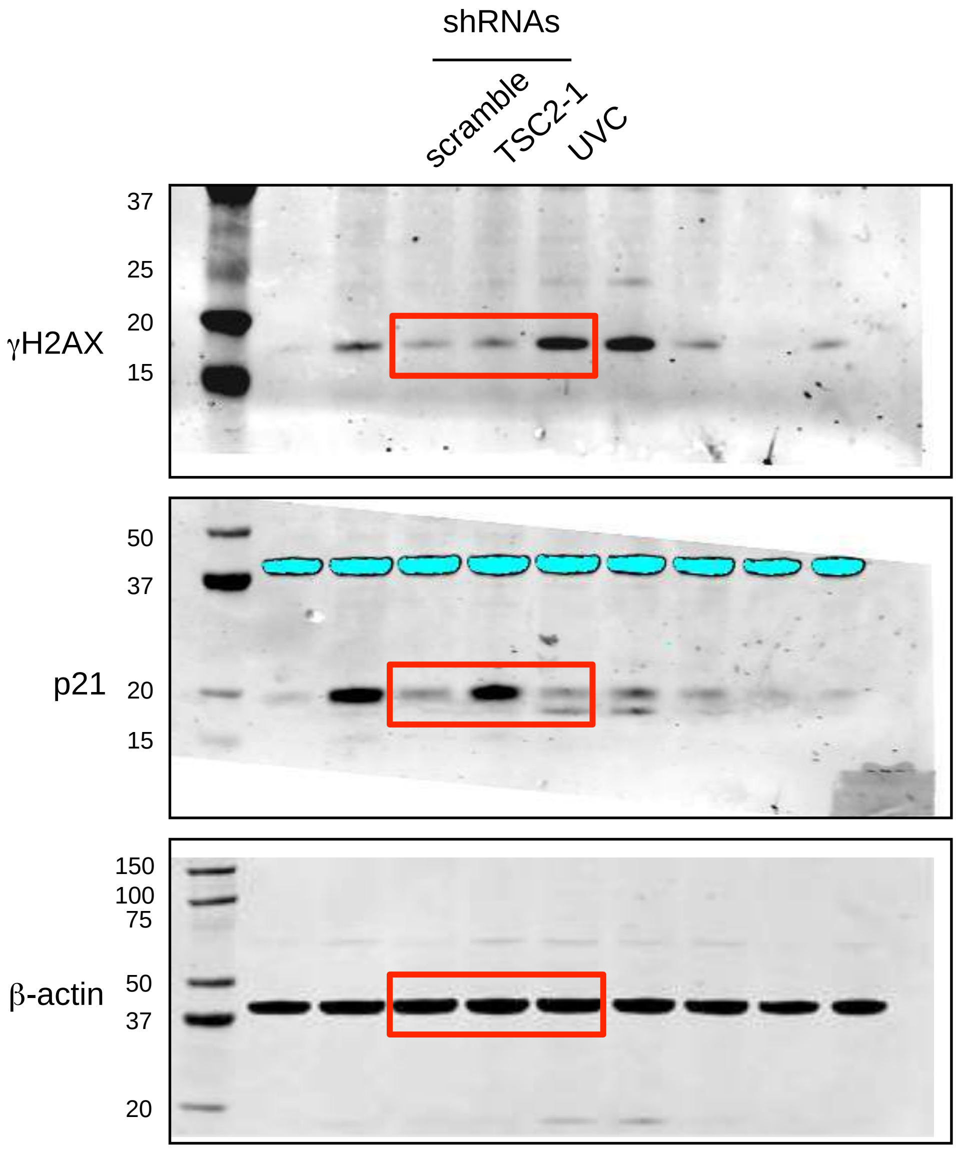

Uncropped membranes for Supplementary Fig. 1b

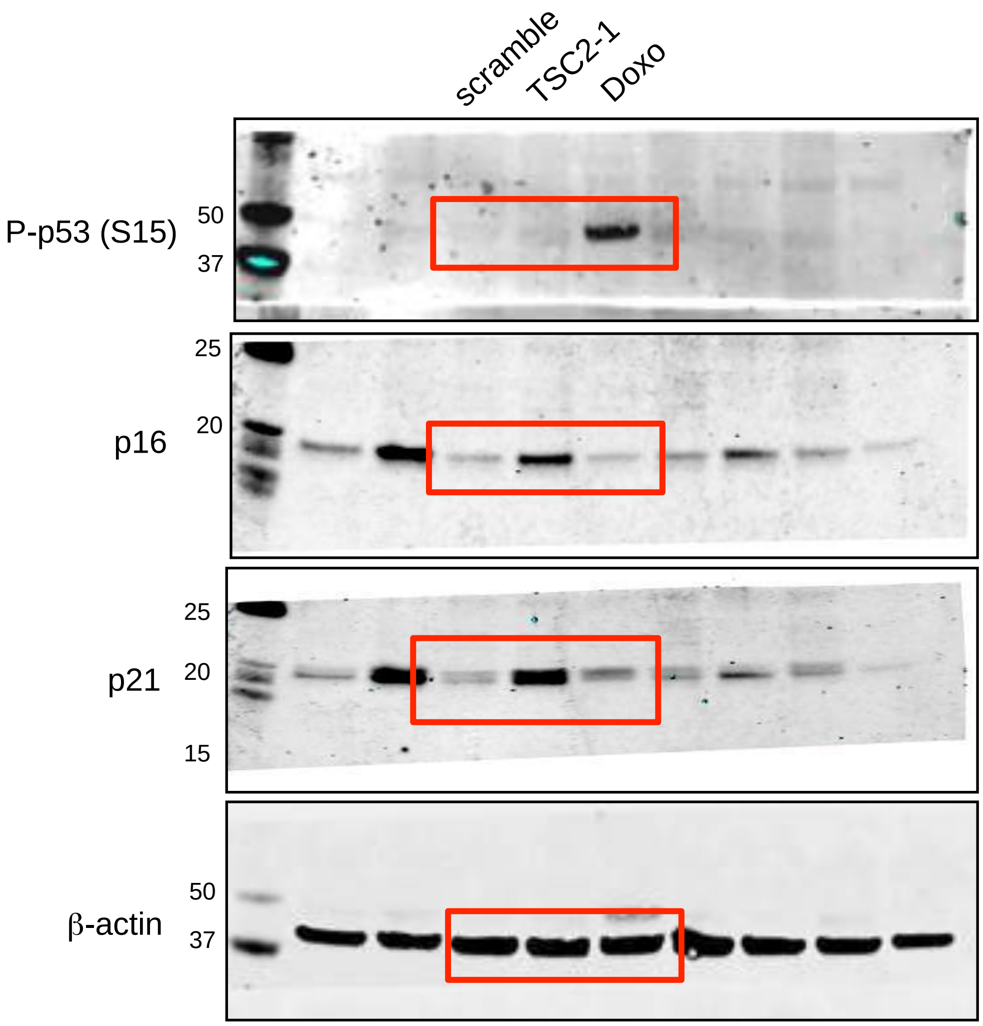

Uncropped membranes for Supplementary Fig. 1c

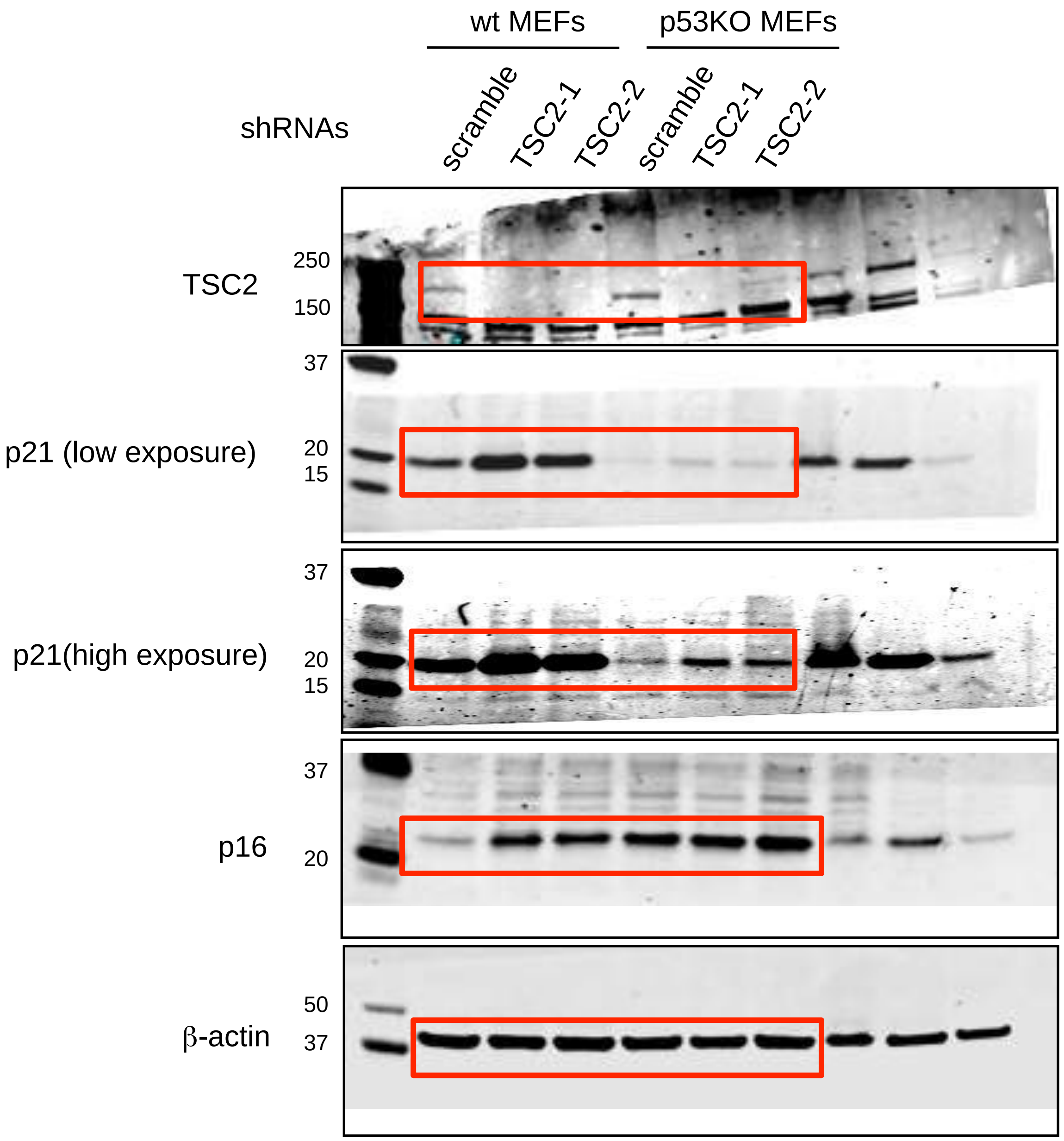

Uncropped membranes for Supplementary Fig. 1d

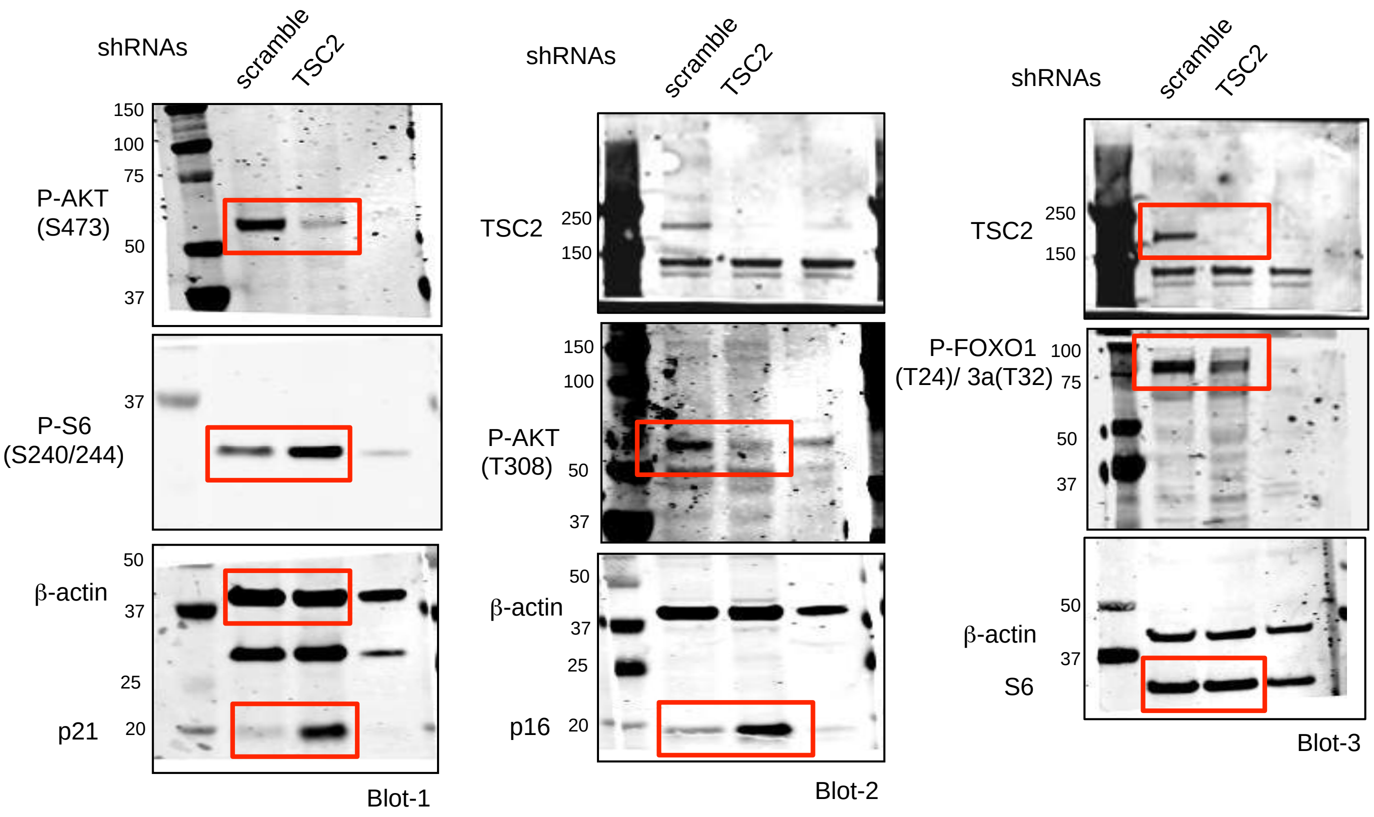

Uncropped membranes for Supplementary Fig. 3a

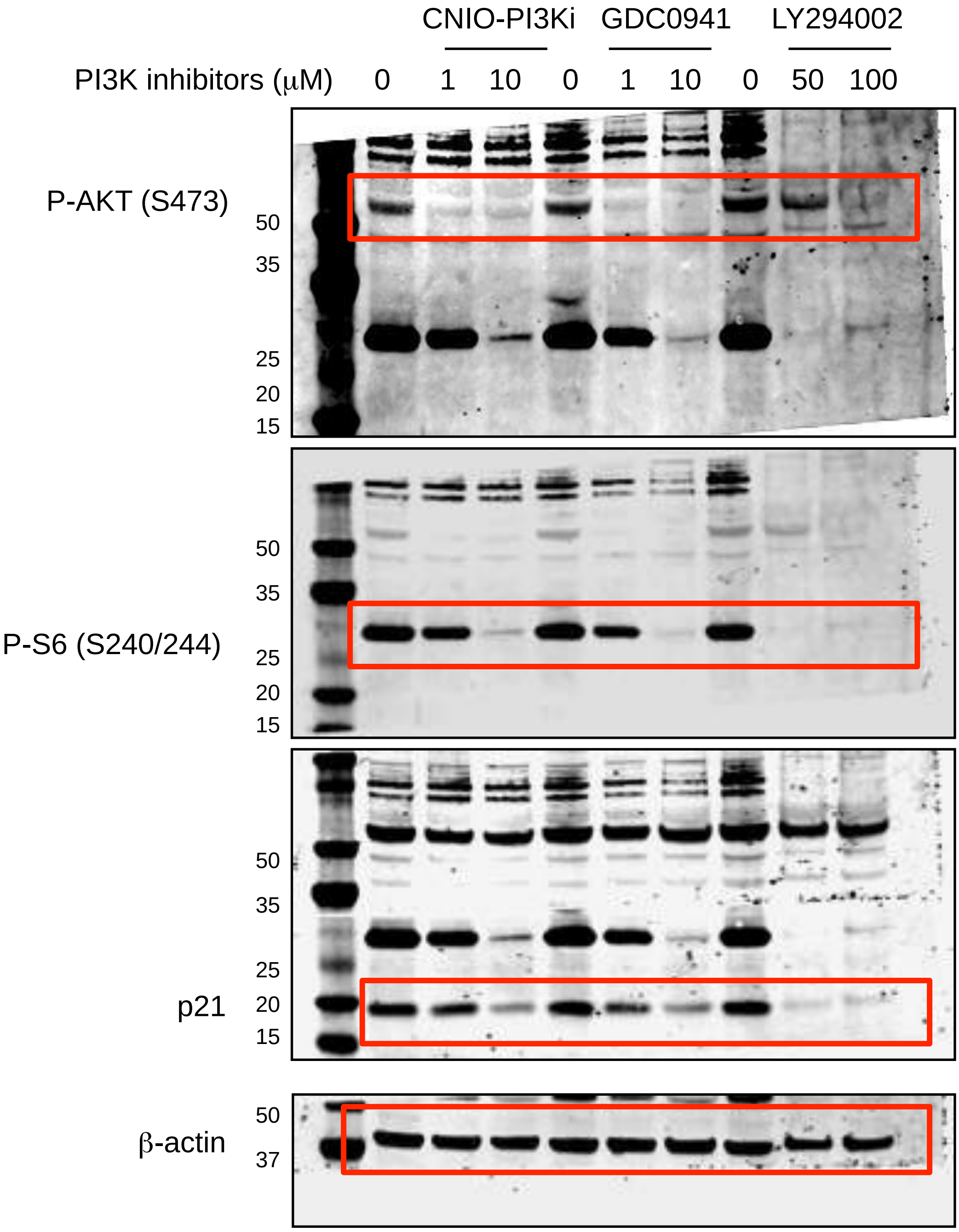

Uncropped membranes for Supplementary Fig. 3b

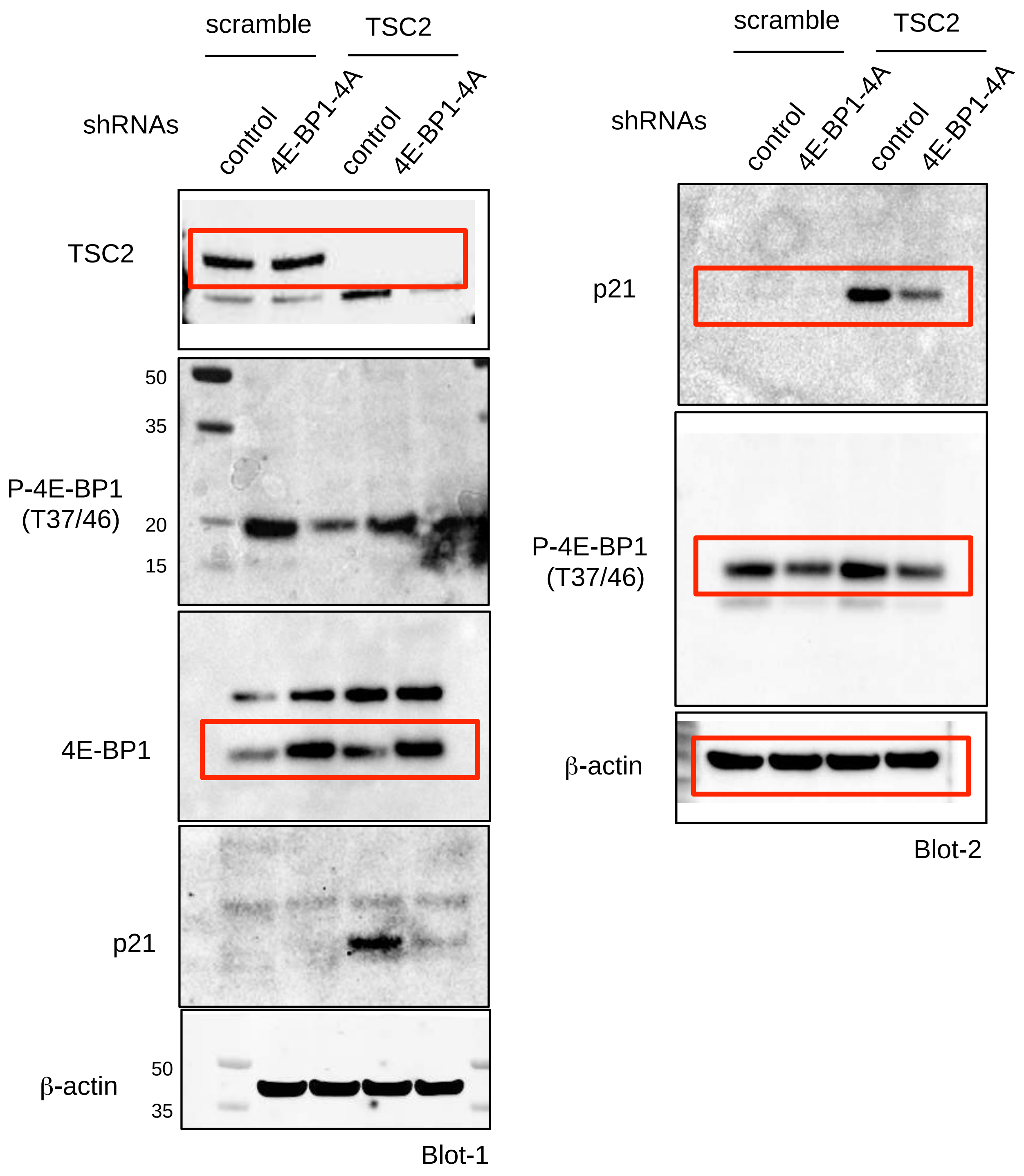

Uncropped membranes for Supplementary Fig. 3c

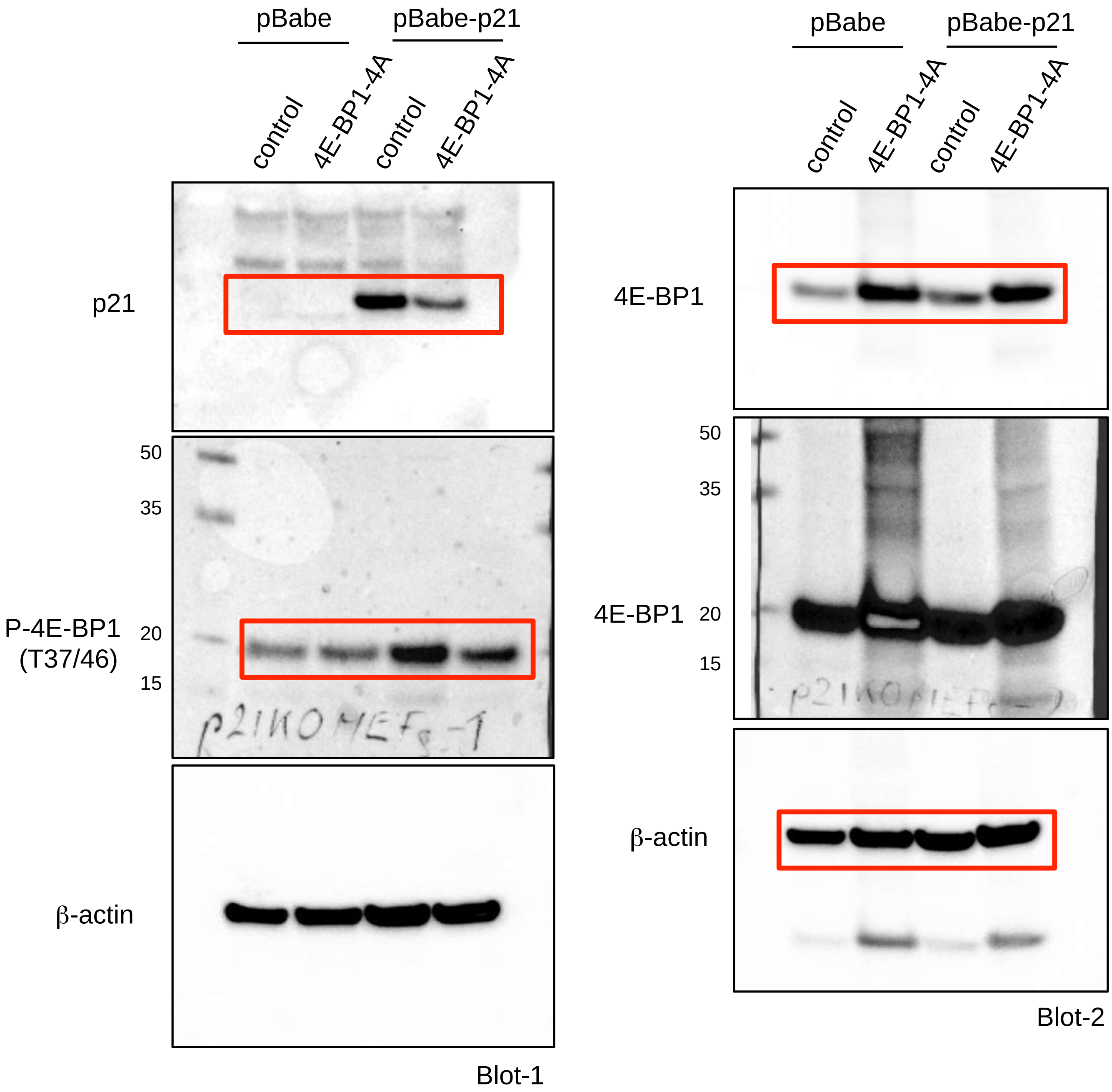

Uncropped membranes for Supplementary Fig. 3d

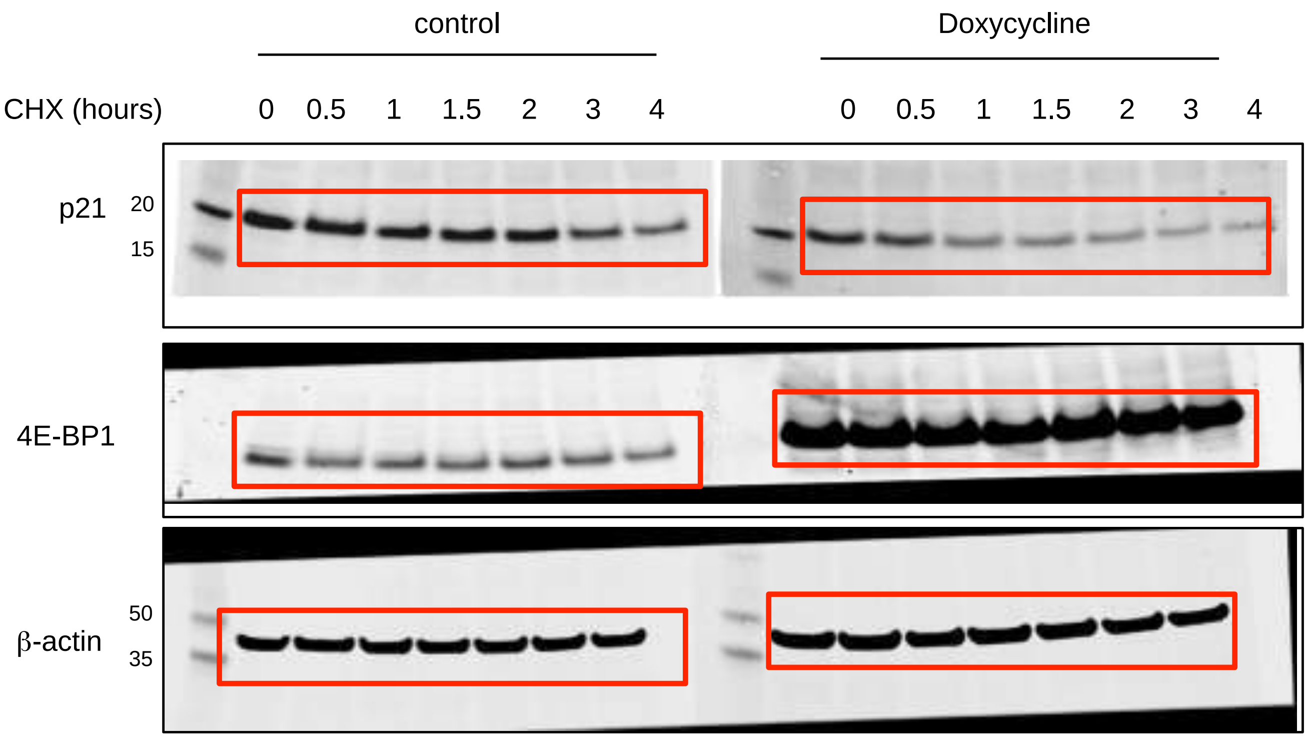

Uncropped membranes for Supplementary Fig. 3e

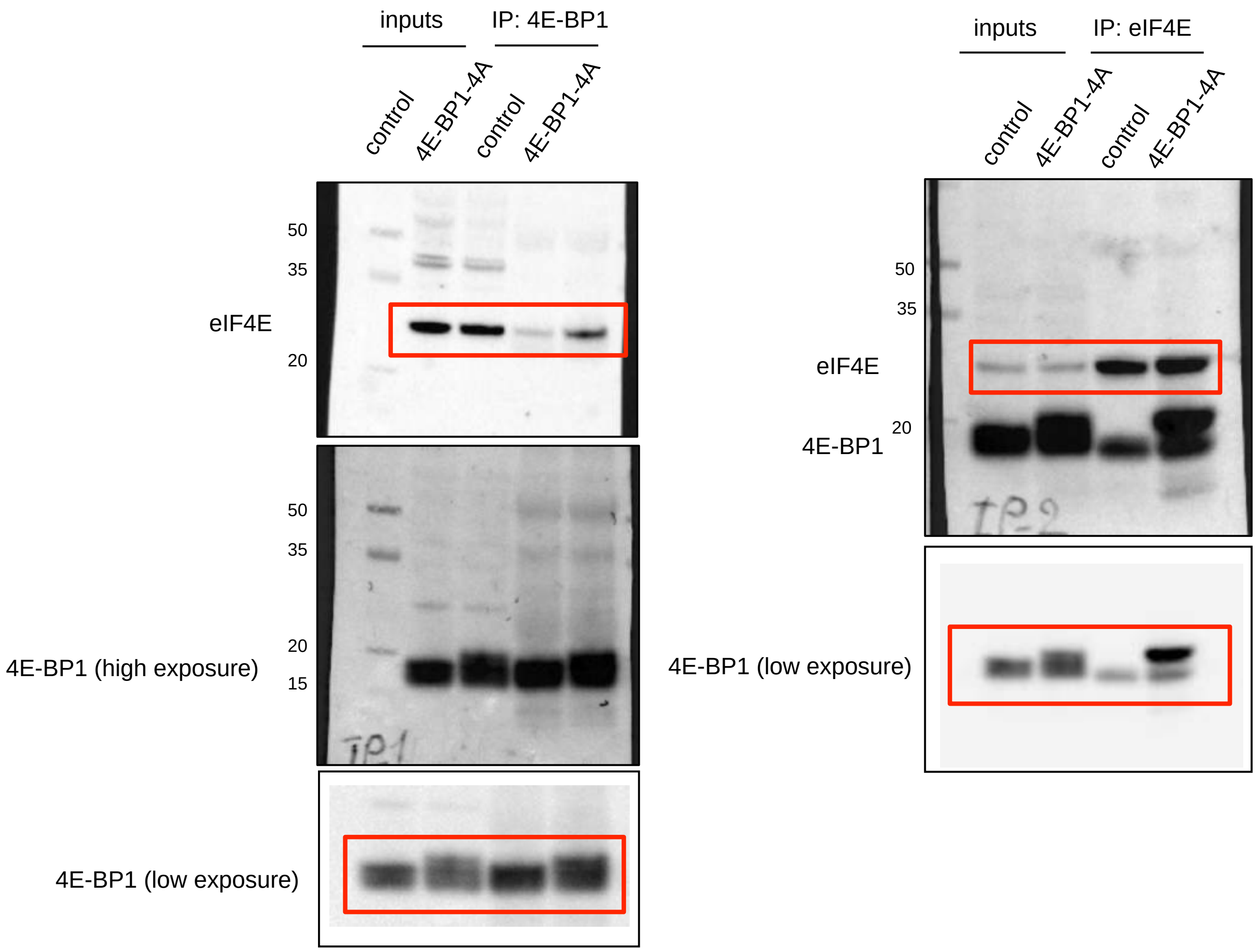

Uncropped membranes for Supplementary Fig. 3f

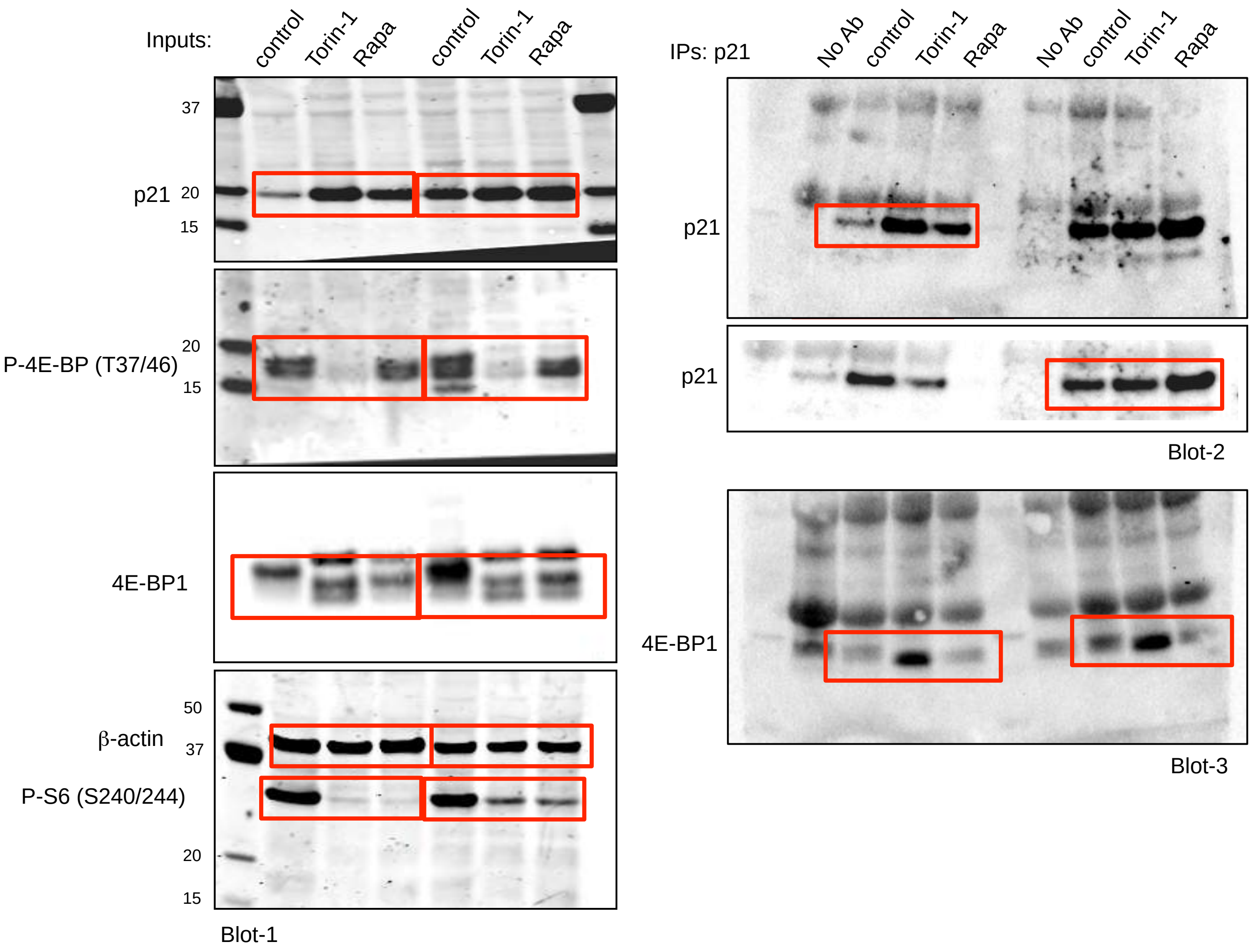

Uncropped membranes for Supplementary Fig. 4a

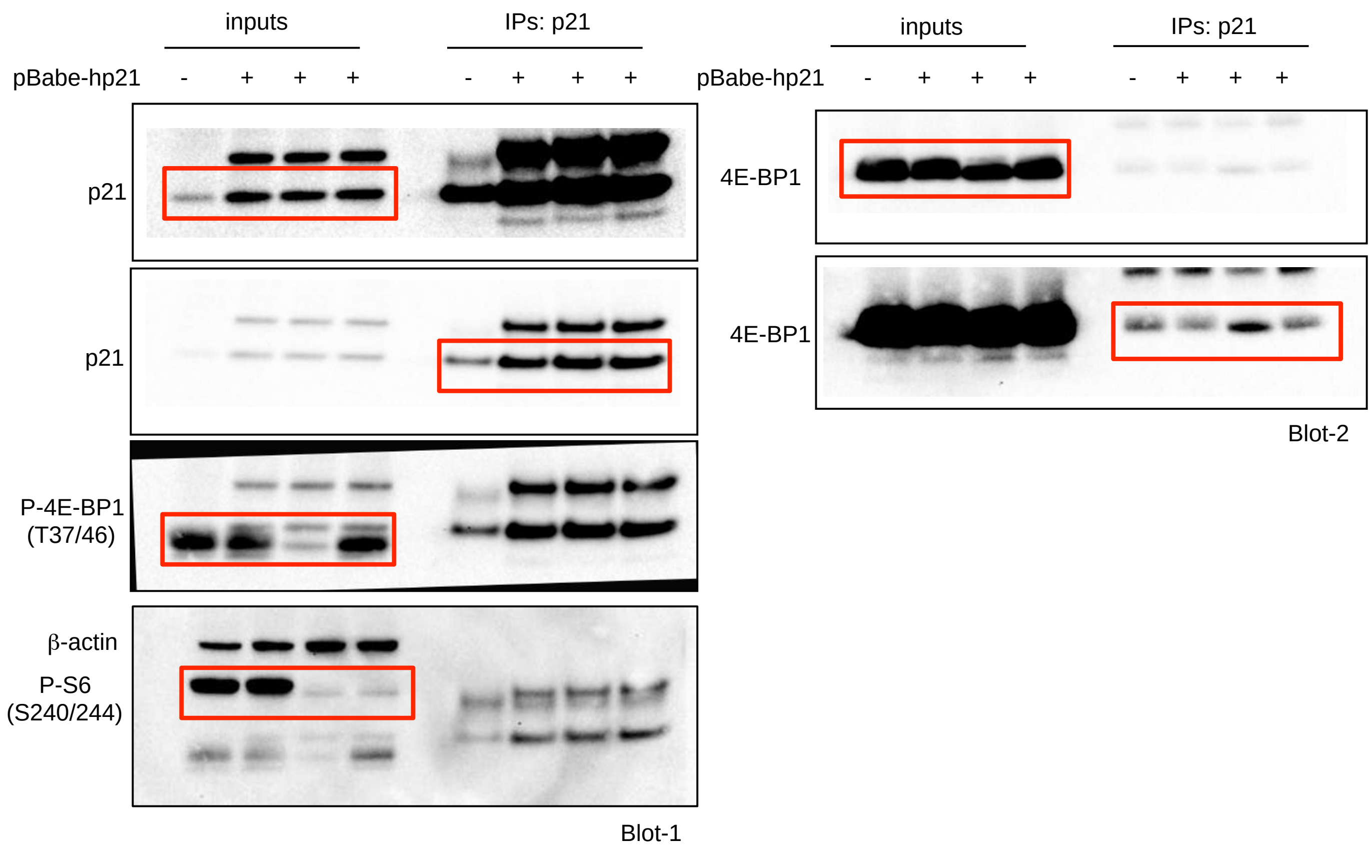

Uncropped membranes for Supplementary Fig. 4b

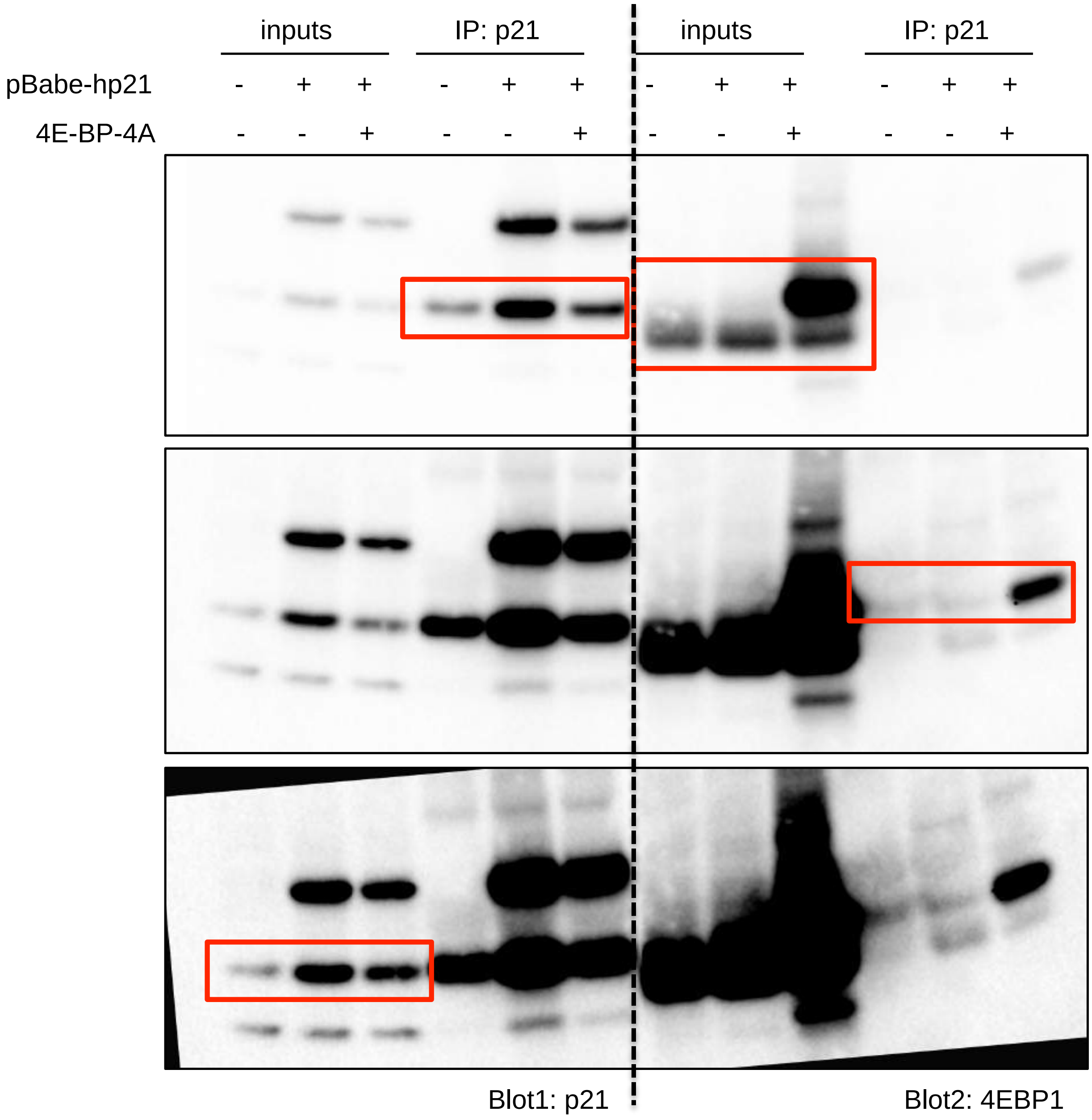

Uncropped membranes for Supplementary Fig. 4c

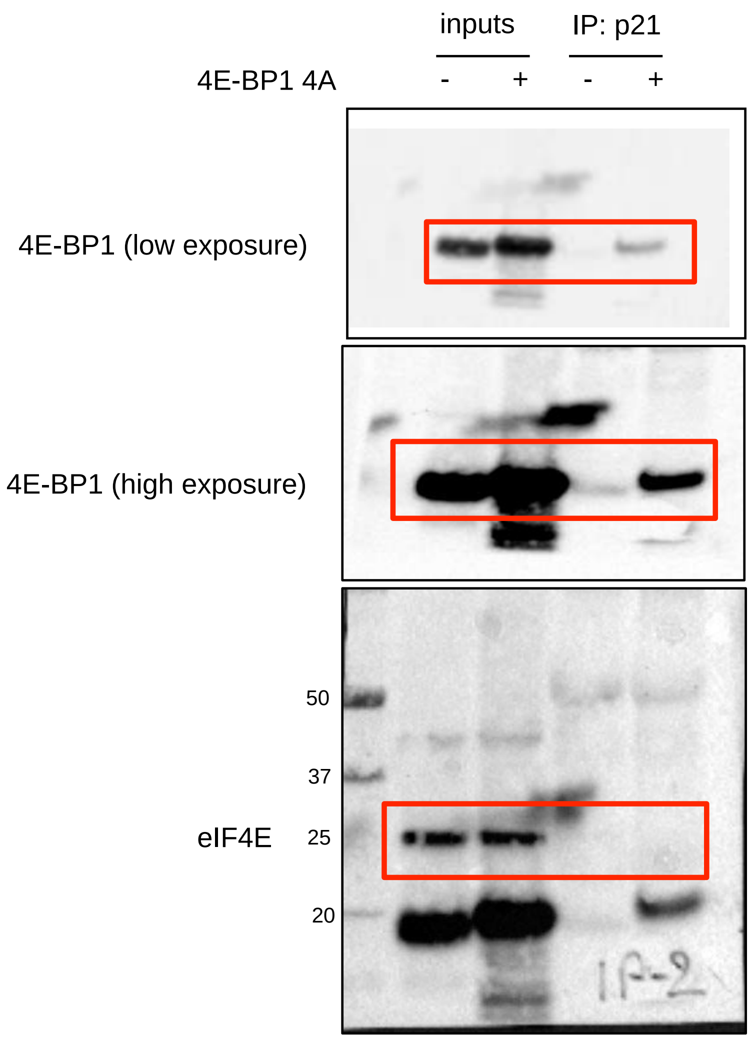

Blot-1

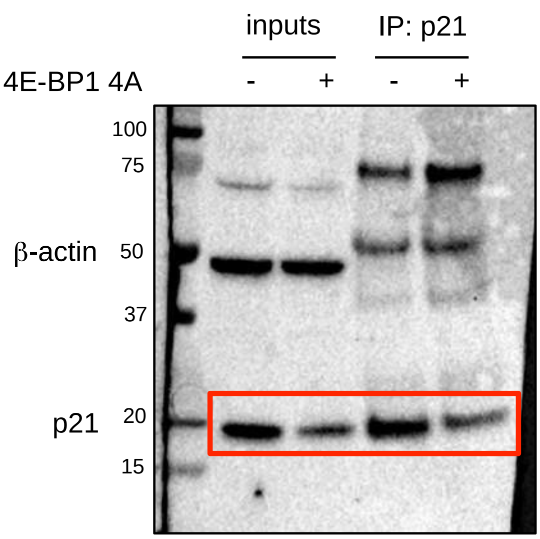

Blot-2

Uncropped membranes for Supplementary Fig. 4d

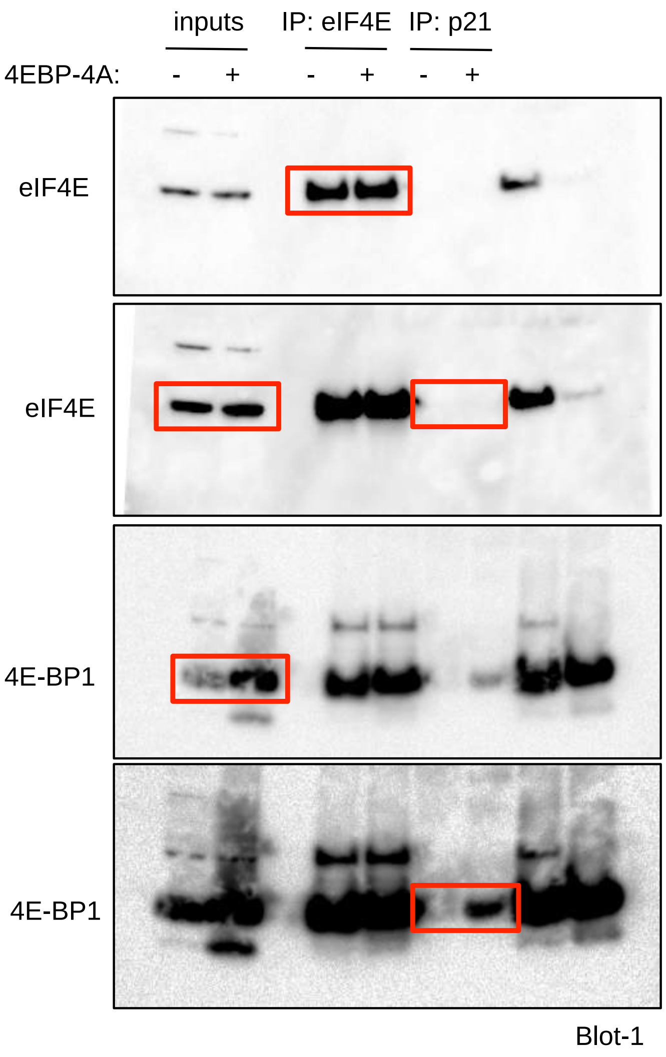

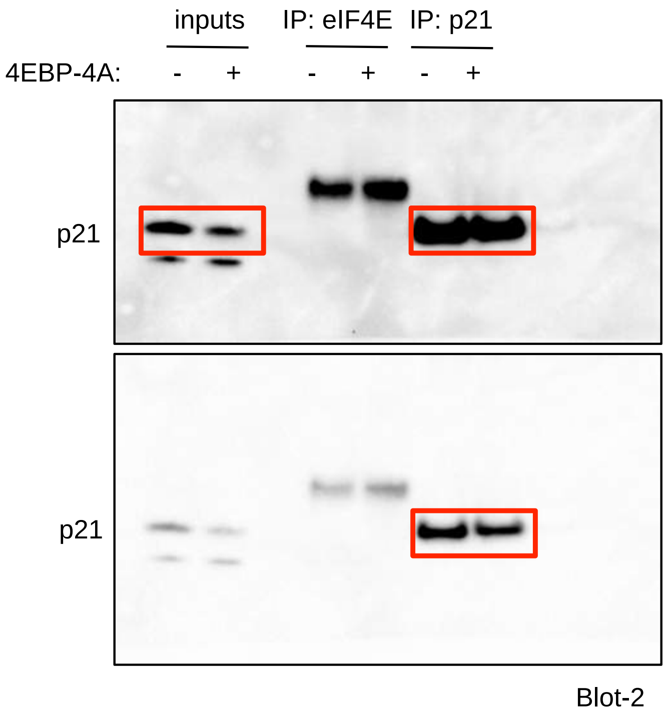

Uncropped membranes for Supplementary Fig. 4e

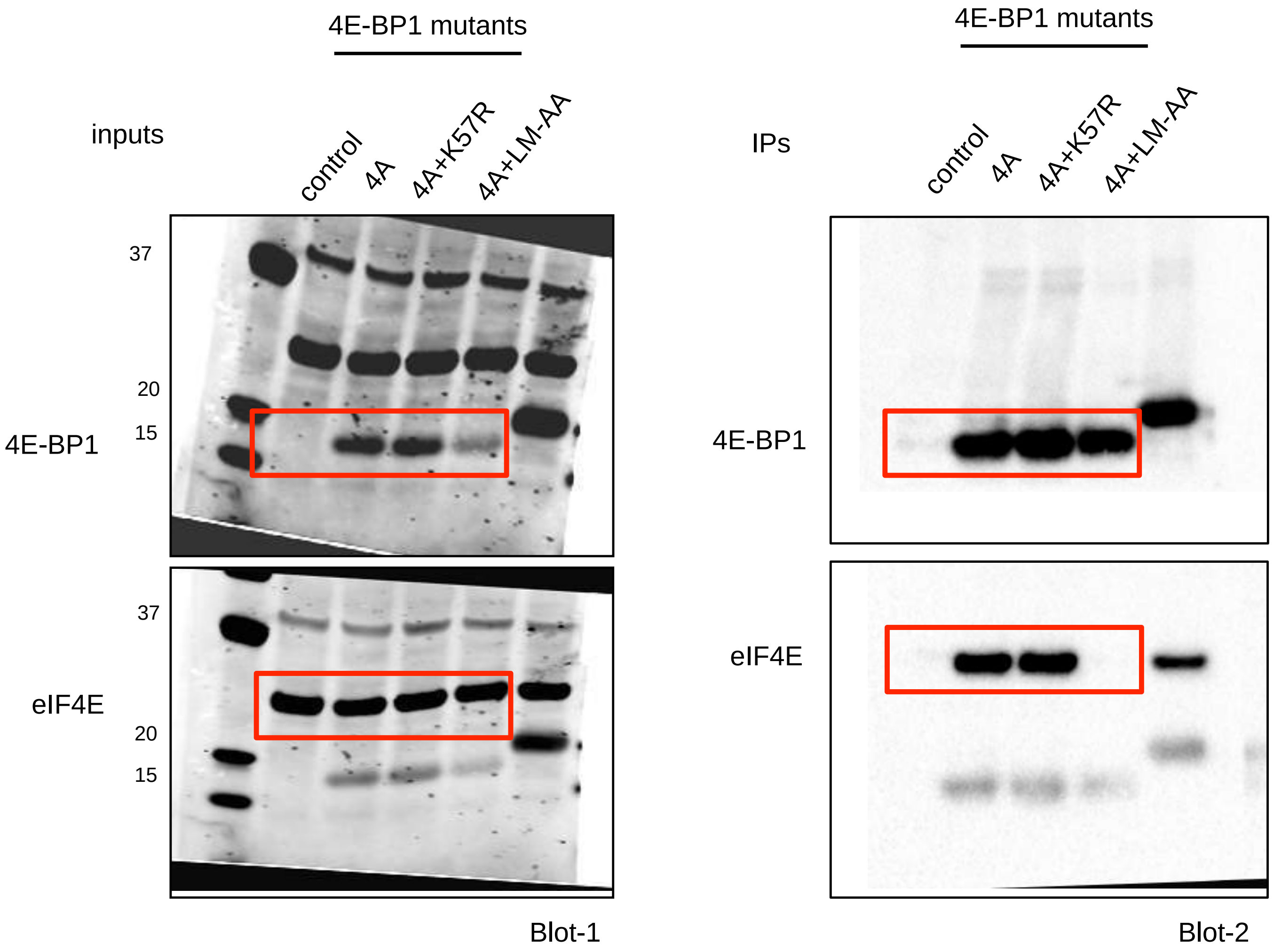

Uncropped membranes for Supplementary Fig. 4f

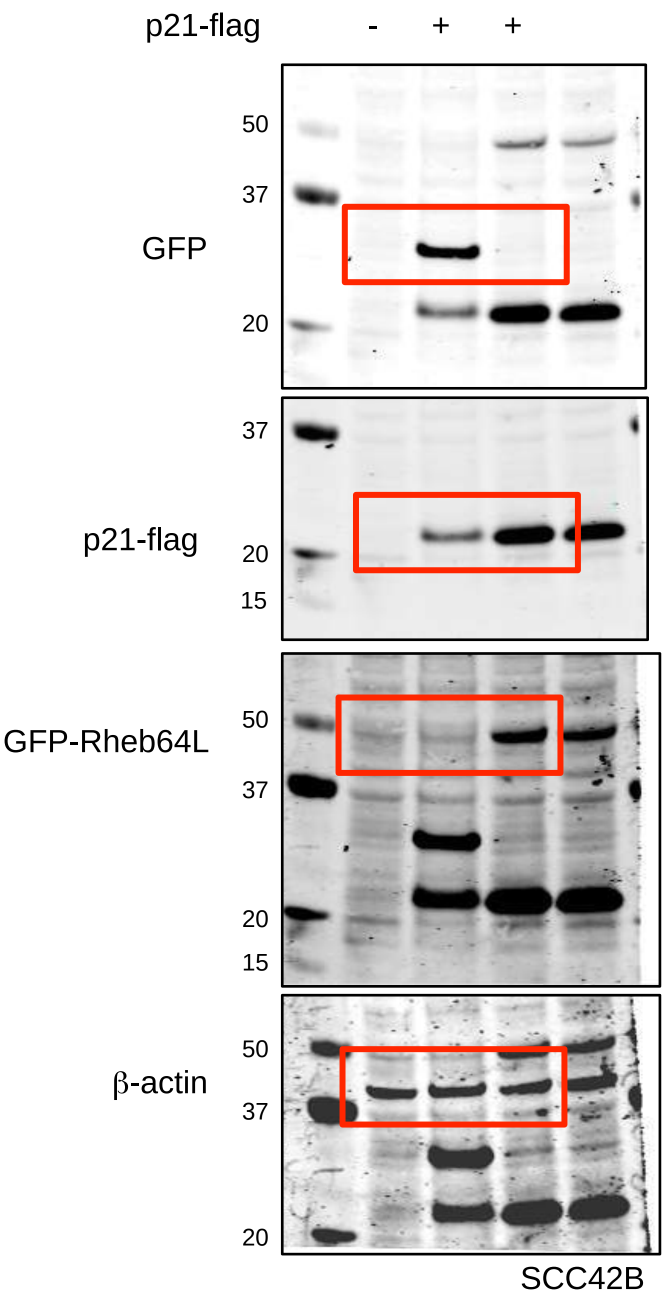

Uncropped membranes for Supplementary Fig. 5a

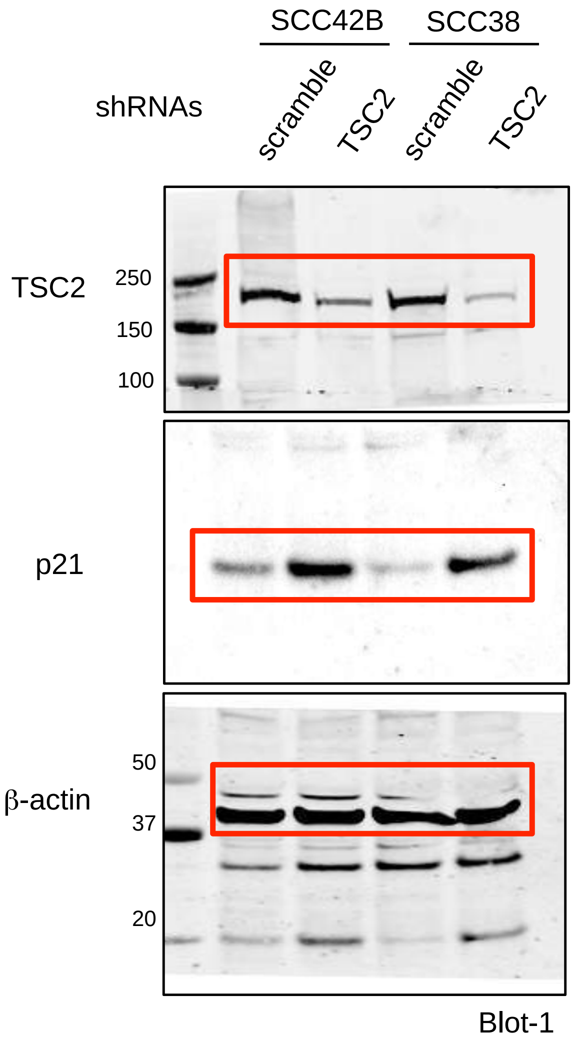

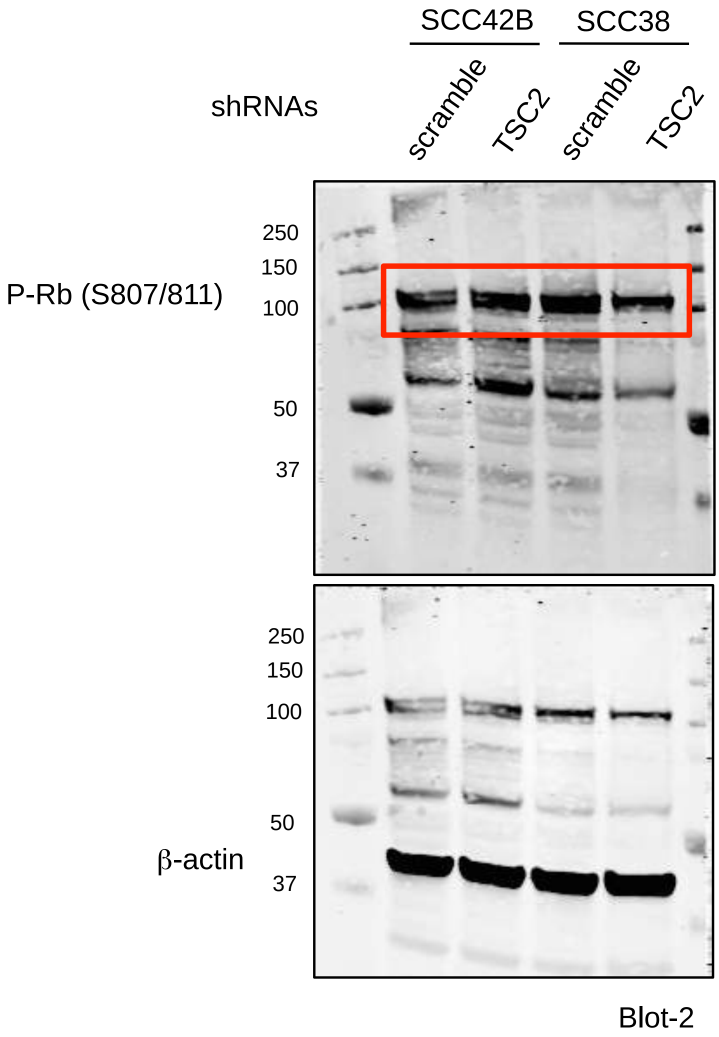

Uncropped membranes for Supplementary Fig. 5b (1)

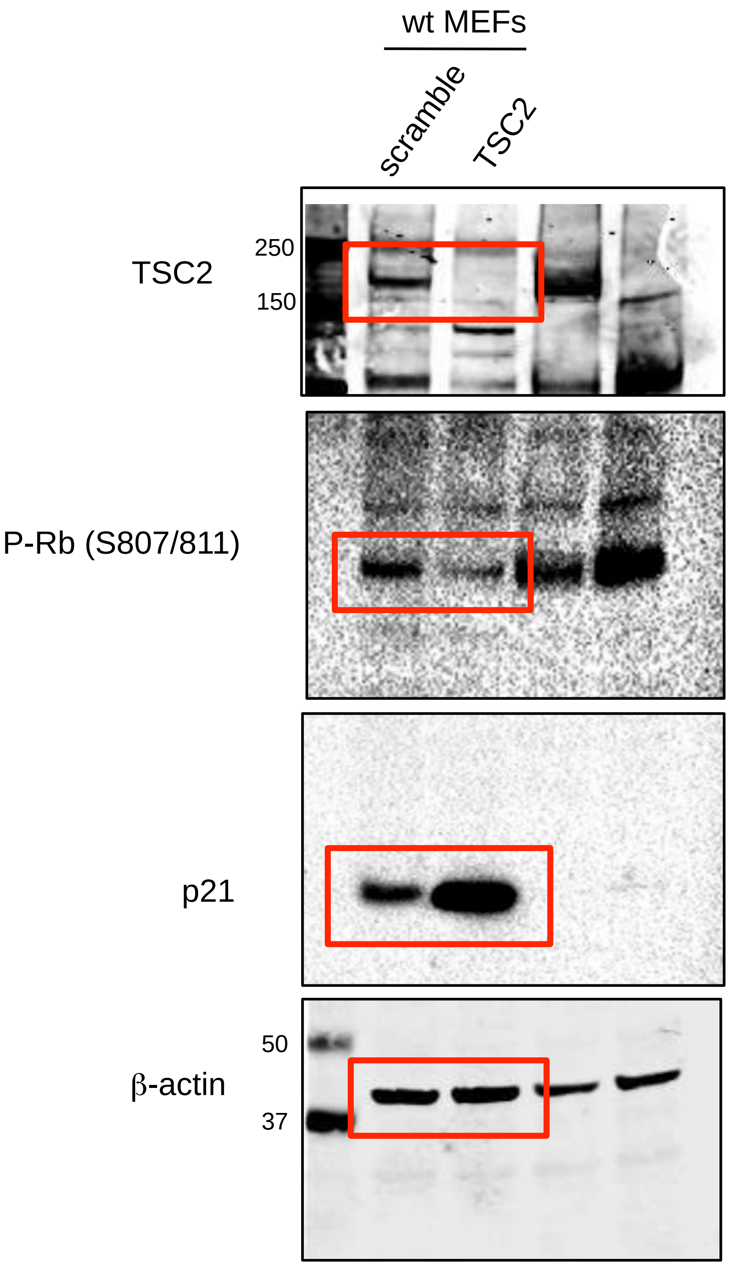

Uncropped membranes for Supplementary Fig. 5b (2)

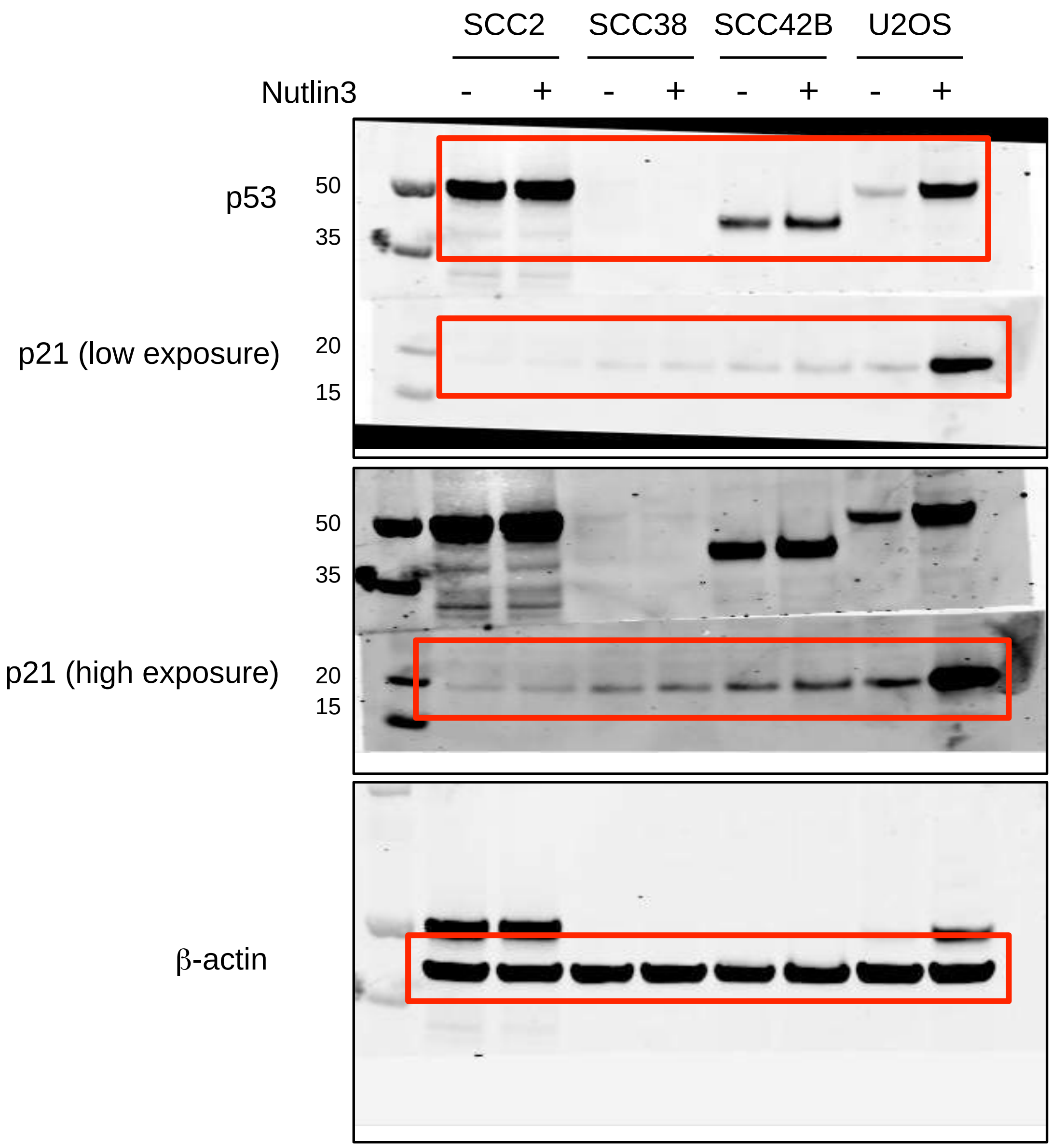

Uncropped membranes for Supplementary Fig. 5d
